# Synaptamide activates the adhesion GPCR GPR110 (ADGRF1) through GAIN domain binding

**DOI:** 10.1038/s42003-020-0831-6

**Published:** 2020-03-06

**Authors:** Bill X. Huang, Xin Hu, Heung-Sun Kwon, Cheng Fu, Ji-Won Lee, Noel Southall, Juan Marugan, Hee-Yong Kim

**Affiliations:** 1 Laboratory of Molecular Signaling, National Institute on Alcohol Abuse and Alcoholism, NIH, 5625 Fishers Lane, Rockville, MD 20852 USA; 2 Division of Preclinical Innovation, National Center for Advancing Translational Sciences, NIH, 9800 Medical Center Dr., Rockville, MD 20892 USA

**Keywords:** Molecular modelling, G protein-coupled receptors, Mass spectrometry

## Abstract

Adhesion G protein-coupled receptors (aGPCR) are characterized by a large extracellular region containing a conserved GPCR-autoproteolysis-inducing (GAIN) domain. Despite their relevance to several disease conditions, we do not understand the molecular mechanism by which aGPCRs are physiologically activated. GPR110 (ADGRF1) was recently deorphanized as the functional receptor of *N*-docosahexaenoylethanolamine (synaptamide), a potent synaptogenic metabolite of docosahexaenoic acid. Thus far, synaptamide is the first and only small-molecule endogenous ligand of an aGPCR. Here, we demonstrate the molecular basis of synaptamide-induced activation of GPR110 in living cells. Using in-cell chemical cross-linking/mass spectrometry, computational modeling and mutagenesis-assisted functional assays, we discover that synaptamide specifically binds to the interface of GPR110 GAIN subdomains through interactions with residues Q511, N512 and Y513, causing an intracellular conformational change near TM6 that triggers downstream signaling. This ligand-induced GAIN-targeted activation mechanism provides a framework for understanding the physiological function of aGPCRs and therapeutic targeting in the GAIN domain.

## Abstract (web summary)

Huang et al clarify the molecular mechanism of activation of adhesion G protein-coupled receptor GPR110 by synaptamide, the only small-molecule endogenous ligand known for this class of GPCR. They find through chemical cross-linking mass spectrometry, modeling and mutagenesis that synaptamide binds to residues in the GAIN domain and induces a conformational change triggering downstream signaling.

## Introduction

Adhesion G-protein-coupled receptors (aGPCR or ADGR) represent the second-largest GPCR family with 33 members. These receptors have a large extracellular region (ECR) containing a conserved GPCR-autoproteolysis inducing (GAIN) domain (~320 amino acids) as well as adhesion-related domains^1,2^. Studies have revealed that in the most C-terminal region of the GAIN domain there exists a “Stachel” or “stalk” sequence located proximal to the seven-transmembrane domain (7TM)^3,4^. aGPCRs have been shown to regulate diverse physiological processes and to be associated with various human disease conditions such as bilateral frontoparietal polymicrogyria and usher syndrome, thus presenting a potential for drug discovery^5–11^. At present, no aGPCR-targeting drugs have been reported, in part because most aGPCRs remain orphan and the molecular mechanism of aGPCRs activation is poorly understood^12^. aGPCR signaling has been proposed to involve GAIN-mediated autoproteolysis and Stachel agonism^3,4^. Cleavage at the GPCR proteolytic site within the GAIN domain results in an extracellular *N*-terminal fragment (NTF) and a *C*-terminal fragment (CTF) containing the “Stachel” sequence, which is thought to activate signaling activity of aGPCRs through interaction with the 7TM^4,13^. Stachel-dependent signaling effects can occur independent of autoproteolysis of the GAIN domain of individual aGPCRs^14^, which was also demonstrated in vivo for latrophilin (ADGRL) in a genetically modified animal model^15^. In addition, a mechanism independent of either the autoproteolysis or Stachel agonism has been suggested for activation of aGPCRs in vitro^16,17^ and recently in vivo^18^. In this model, the binding of a ligand to the ECR is thought to modulate G protein signaling through conformational changes at the 7TM^17^. Of note, the conserved GAIN domain is not directly involved in the ligand binding^17,18^.

G-protein-coupled receptor 110 (GPR110, or ADGRF1) is an orphan receptor that belongs to the aGPCR subfamily VI and was initially recognized as an oncogene implicated in lung and prostate cancers^19^. Recently, GPR110 was discovered to be a functional receptor for *N*-docosahexaenoylethanolamine (synaptamide), an endogenous metabolite of docosahexaenoic acid (DHA, 22:6n-3, an omega-3 fatty acid), a lipid highly enriched in the brain^20^. The binding of synaptamide to GPR110 triggers cAMP-dependent signal transduction, promoting neurogenesis, neurite growth and synaptogenesis in developing neurons. Interestingly, a double mutation at the GPCR proteolytic site (H565A/T567A) of GPR110 that prevents autocleavage does not alter ligand binding or synaptamide-induced cAMP production^20^. Considering that T567 is highly conserved in all Stachel regions across the entire aGPCR family and the deletion of this residue has been shown to impair or abolish the Stachel-dependent signaling both in vitro and in vivo^3,4,15^, the GPR110 activation by synaptamide most likely signals via a Stachel-independent mechanism. Co-immunoprecipitation indicates that synaptamide interacts with the *N*-terminal fragment but not with the *C*-terminal fragment. In addition, application of recombinant *C*-terminal fragment, which contains the exposed Stachel sequence, fails to potentiate or alter cAMP production in response to synaptamide^20^. These findings further support that GPR110 activation by synaptamide is governed in an autoproteolysis- or Stachel-independent manner. Thus, a detailed molecular understanding of GPR110 activation remains elusive.

Chemical cross-linking combined with mass spectrometry has proven to be a useful tool for probing the three-dimensional (3D) structure of proteins, supplementing conventional approaches such as X-ray crystallography and nuclear magnetic resonance (NMR) spectroscopy^21–25^. A cross-linker is used to capture reactive amino acid residues by covalently binding them together. Based on the sites of cross-linking identified by mass spectrometry (MS) and the distance constraint imposed by the cross-linker, the spatial distance or the 3D structural information of the protein is deduced^26^. One distinct feature of cross-linking approaches is their ability to monitor the conformational dynamics of a protein under physiologically-relevant conditions^27^.

In this study, we unveil a physiologically-relevant molecular mechanism for GPR110 activation using 3D structural probing, computational modeling, site-directed mutagenesis and biological activity assays. We probe GPR110 structure in living cells using in-cell chemical cross-linking coupled with mass spectrometric analysis. With the help of computational modeling and site-directed mutagenesis, we demonstrate that the small-molecule ligand synaptamide binds to the GPR110 GAIN domain and causes an intracellular conformational change in living cells, revealing a previously unknown molecular mechanism of aGPCR activation through ligand-GAIN domain interaction.

## Results

### Probing 3D structure of GPR110 in living cells

**Fig. 1:**
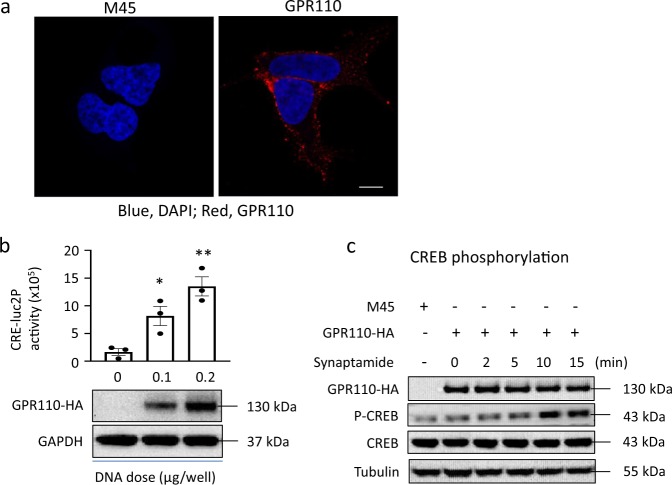
Expression and bioactivity of GPR110-HA. **a** Detection of overexpressed GPR110-HA in the plasma membrane. Scale bar: 5 μM. **b** GPR110 bioactivity induced by synaptamide. Synaptamide (10 nM) increased the cAMP production depending on the expression of GPR110. Data are means ± SEM of biological triplicates. Statistical analysis was performed using Student’s *t*-test. *p < 0.05; **p < 0.01 (*n* = 3 independent experiments). **c** Time-dependent phosphorylation of CREB in GPR110-transfected HEK cells.

**Fig. 2:**
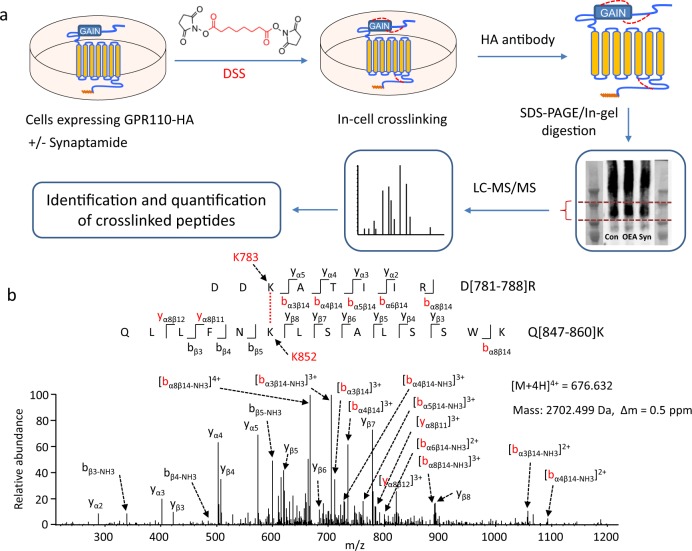
Probing GPR110 conformation in living cells by chemical cross-linking and mass spectrometry. **a** Experimental scheme. HEK293 cells overexpressing GPR110-HA were incubation with disuccinimidyl suberate (DSS). Cells were lysed and GPR110 was pulled down with HA antibody and eluted with HA peptide. After SDS-PAGE and Coomassie staining, the monomeric protein band was excised and subjected to reduction/alkylation, tryptic digestion and mass spectrometric analysis. DSS-modified peptides were identified by xQuest software with manual validation of MS/MS data and quantitated by Progenesis QI for Proteomics. The HA-tag in GPR110 structure is marked with orange and the cross-linking by DSS is depicted with red dotted lines. **b** Representative MS/MS analysis of cross-linked peptides. The MS/MS data revealed that the peptide with mass of 2702.499 Da reconstructed from quadruply charged ion of *m/z* 676.632, originated from D[781-788]R of TM6 and Q[847-860]K of the *C*-terminus with K783 linked to K852. Fragment ions involving both peptide segments (designated as α and β, respectively) are marked in red. The cross-link was identified in both synaptamide-treated and non-treated control samples.

Human GPR110 tagged with HA at the *C*-terminal (GPR110-HA) was overexpressed in HEK cells, and the expression of GPR110-HA in the plasma membrane was verified by immunocytochemistry (Fig. 1a). Synaptamide-induced GPR110 activity was confirmed by gene-dose-dependent cAMP production detected in CRE-luc2P HEK 293 cells, which contain a luciferase gene (luc2P) as the cAMP sensor (Fig. 1b, Supplementary Data 1) as well as phosphorylation of downstream cyclic AMP response element binding protein (CREB)^20^ (Fig. 1c, Supplementary Fig. 1). Cross-linking of expressed GPR110 was carried out in cells with or without synaptamide treatment using disuccinimidyl suberate (DSS), a lysine-specific cross-linker with the arm length of 11.6 Å (Fig. 2a). The DSS-modified GPR110-HA was pulled down with HA antibody and the DSS-modified monomeric protein was separated by SDS-PAGE and subjected to liquid chromatography-mass spectrometric analysis after in-gel tryptic digestion (Fig. 2a, Supplementary Fig. 2). Tryptic peptides from GPR110 were identified by MS, covering around 60% of the sequence from the *N-* and *C*-terminal regions, and ~5% of the sequence from the 7TM domain (Supplementary Fig. 3). The MS/MS analysis unambiguously revealed 25 intramolecular cross-linked peptides including twelve through-space cross-linked pairs (Fig. 2b, Supplementary Figs. 4, 5, Supplementary Table 1) in both synaptamide-treated and non-treated control samples. Among those through-space cross-linked pairs, 11 involved two peptide segments in the ECR, including K29-K38 in the *N*-terminal region (Nter, AA 1-145), K151-K187, K151-K254, K187-K240, and K240-K254 in the SEA domain (AA ~148-256) and K398-K427, K398-K438, K398-442, K427-K438, and K427-K442 in the GAIN domain (AA ~251–580), as well as K151-K442, which represents the cross-linking between the SEA and GAIN domains. As shown by the MS/MS spectrum in Fig. 2b, a through-space cross-linking was identified between K852 and K783. These residues are located in the *C*-terminal region and the cytoplasmic end of TM6, respectively, based on PSI-blast-based secondary structure prediction (PSIPRED)^28^. This inter-domain cross-linking revealed the spatial proximity between TM6 helix and the *C*-terminal region where G proteins are known to interact with GPCRs^29^. In addition, 9 and 4 loop-links within single peptide segments in the ECR and the *C*-terminal regions were detected, respectively. The Cα–Cα distance between each cross-linked lysine pair is estimated to be within ~24 Å, providing further spatial distance information of GPR110 molecular structure.

### Ligand-induced conformational changes of GPR110 in cells

Monitoring the observed cross-links allowed us to probe the conformational changes of GPR110 at different activation stages. Based on the activity assay shown in Fig. 1, GPR110-overexpressing cells were stimulated with synaptamide for 10 min for GPR110 activation, while DMSO (vehicle) or oleoylethanolamine (OEA), an inactive structural analog of synaptamide, was used in parallel as an unstimulated control^20^. The ligand-induced changes in GPR110 conformation were deduced from comparative analysis of the cross-linked peptides and monolinks using label-free quantitation^30,31^ (Table 1, Supplementary Tables 2, 3). A total of 13 monolinks were detected, including K398 and K438 in the GAIN domain, and K783 at TM6. None of the monolinks were significantly altered after ligand binding (Supplementary Table 3), indicating the accessibility of these lysine residues did not change. Monolinks were not detected for 12 lysine residues such as K852 in the *C*-terminal region that participated in cross-linking (Table 1). However, based on the unaltered cross-linking profile upon ligand binding shown in Table 1, major changes in the accessibility is unlikely for these lysine residues. Among a total of 25 cross-links across the entire GPR110 structure, only the cross-linking of K398-K438 in the GAIN domain, and the inter-domain cross-linking of K783-K852 in the intracellular regions, increased significantly after stimulation with synaptamide compared to the case with the OEA- or DMSO-treated control (fold-change ≥ 1.5 and *p* ≤ 0.05)^30,31^. As the accessibility of K398, K438, K783, and presumably K852 remained unchanged, changes in the cross-linking profile for K398-K438 and K783-K852 are most likely due to changes in proximity between the cross-linked pairs. In other words, residues K398 and K438 and residues K852 and K783 became closer to each other after synaptamide treatment, facilitating improved cross-linking reactions^32^. The change of proximity between K398 and K438 indicates that a local conformational change took place in the GAIN domain upon binding of synaptamide to the receptor. None of the cross-links in other ECR regions, including the SEA domain and the *N*-terminal region, were altered indicating that synaptamide binds specifically to the GAIN domain. The synaptamide-induced change in the cross-linking of K783-K852 indicates an alteration in the intracellular configuration of the receptor involving TM6 and the *C*-terminal regions where G-protein interaction is presumed to occur. These data suggest that synaptamide binding to the GAIN domain may impact the interaction of GPR110 with G-proteins hence the downstream signaling.

**Table 1:** Mass spectrometric quantitation of cross-linked peptides.

Domains	Cross-linked lysine pairs	Synaptamide/OEA		Synaptamide/DMSO	
		Intensity ratio	Student’s *t*-test p (*n* = 3)	Intensity ratio	Student’s *t*-test p (*n* = 3)
N-terminus	K29-K38	0.86	0.18	0.76	0.06
	K31-K32	1.03	0.43	0.89	0.11
	K38-K39	0.96	0.42	0.82	0.10
	K39-K40	1.26	0.19	0.94	0.40
SEA	K151-K157	1.09	0.36	0.80	0.11
	K151-K187	1.03	0.42	0.92	0.33
	K151-K254	1.13	0.22	0.81	0.22
	K186-K187	1.22	0.27	1.00	0.49
	K187-K240	1.09	0.16	0.90	0.12
	K235-K240	0.88	0.05	0.88	0.02
	K240-K254	1.02	0.47	0.71	0.22
SEA-GAIN	K151-K442	0.89	0.31	0.54	0.07
GAIN	K398-K427	0.86	0.29	0.80	0.28
	K398-K438	1.53	0.05	1.90	0.03
	K398-K442	1.02	0.47	0.78	0.19
	K427-K432	1.03	0.37	1.08	0.20
	K427-K438	0.99	0.45	0.90	0.21
	K427-K442	1.08	0.08	0.90	0.05
	K432-K438	0.96	0.35	0.93	0.26
	K438-K442	1.15	0.03	1.02	0.41
TM6-C-terminus	K783-K852	1.71	0.04	1.77	0.01
C-terminus	K852-K860	1.16	0.31	0.83	0.26
	K860-K864	1.17	0.20	0.91	0.23
	K864-K873	1.33	0.07	1.17	0.12
	K875-K878	1.06	0.37	1.18	0.12

### Modeling of the GAIN domain of GPR110

**Fig. 3:**
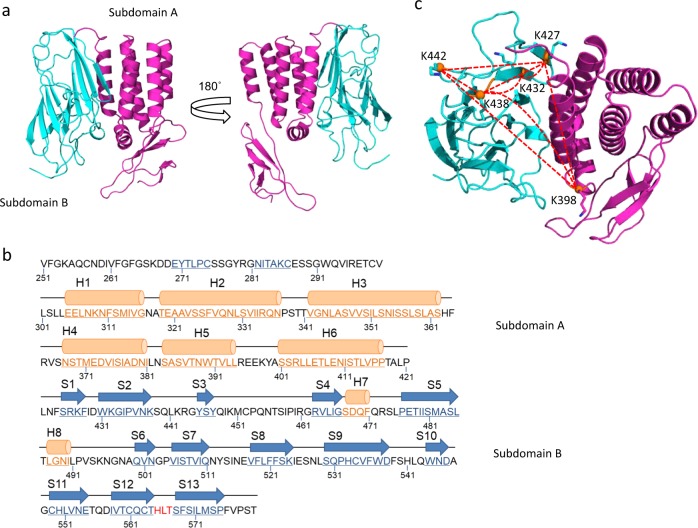
Modeling of GPR110 GAIN domain and the cross-linking profile. **a** Two views of the GAIN domain comprising subdomains A (magenta) and B (cyan). **b** Annotation of the primary sequence of GPR110 GAIN domain with the secondary structure elements predicted in the model. The α-helices (orange colored) and β-strands (blue colored) are labeled as H and S, respectively. Conserved residues in the GPS motif are colored with red. The GAIN domain was modeled with the crystal structure of BAI3 (ADGRB3, pdb 4DLO). **c** Cross-linking profile observed by in-cell chemical cross-linking and mass spectrometry. Eight cross-linked lysine pairs (depicted with red dotted lines) were detected in the GAIN domain with or without ligand stimulation.

To understand the conformational changes inferred by our cross-linking data, we constructed a 3D structure model for the GPR110 GAIN (AA ~251–580) domain based on the crystal structure of brain angiogenesis inhibitor (BAI3 or ADGRB3, pdb 4DLO). GPR110 and BAI3 belong to two separate subfamilies (VI and VII, respectively) of aGPCRs and their GAIN domains share a sequence homology of ~30%. The model reveals a characteristic GAIN domain structure that consists of a subdomain A with 6 α-helices followed by a subdomain B comprising 13 twisted β-strands and two small α-helices (Fig. 3a, b, Supplementary Data 2). Among the five lysine residues participating in the cross-linking in the GAIN domain, K398 is located at the short loop between helix 5 and helix 6 and near the beginning of helix 6 in subdomain A, while K427 (β-strand 1), K432 (β-strand 2), K438 (the end of the β-strand 2), and K442 (the loop between β-strands 2 and 3) are located at the beginning of subdomain B (Fig. 3b, c). Due to conformation dynamics and model inaccuracies, computational C_α_–C_α_ distances of up to 30–35 Å are considered reasonable for DSS cross-linked residues in a model^33–35^. The C_α_–C_α_ distance of seven out of eight cross-links identified in the GAIN domain, including the inter-subdomain link of K398-K438, are within this maximum cross-linking distance of 35 Å (Supplementary Table 4). The structure model is generally consistent with the cross-linking data with an exception of the K398-K442 cross-link, where the C_α_–C_α_ distance was predicted to be 40.4 Å. This specific discrepancy may be explained by uncertainties in the location of K442, which is positioned at the turning point of the β2-β3 loop making it too flexible to be accurately modeled.

### Modeling the interaction of the GAIN domain with synaptamide

**Fig. 4:**
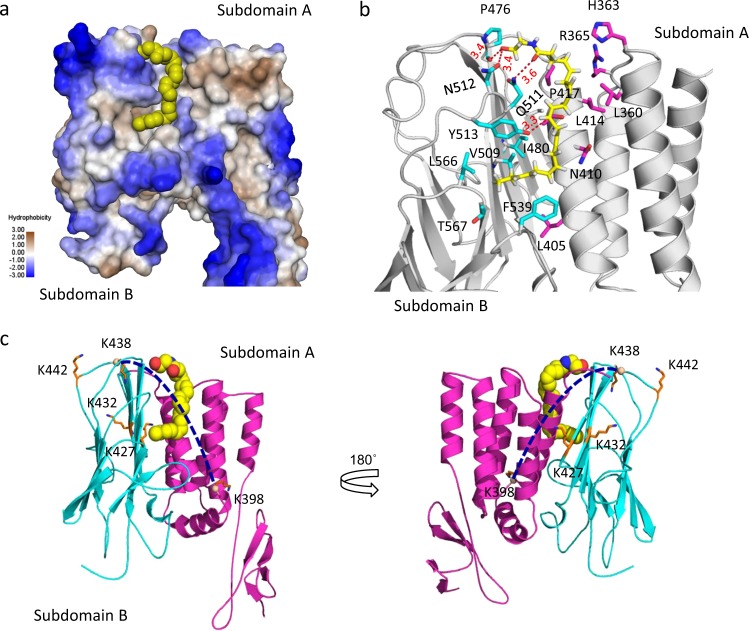
Modeling of the GAIN domain interaction with synaptamide. **a** Synaptamide binding pocket located in the interface of the two subdomains. The protein surface is colored with hydrophobicity (blue, polar; yellow-gray, hydrophobic). **b** Amino acid residues predicted to interact with synaptamide. **c** The inter-subdomain cross-linking of K398-K438 detected by in-cell cross-linking and mass spectrometry. Subdomains A and B are colored with magenta and cyan respectively. Among eight cross-linked lysine pairs observed, only the cross-link of K398-K438 (depicted by dotted line) changed after synaptamide binding. Lysin residues (stick representation) involving in the cross-linking reactions were labeled. The GAIN domain (AA 251–580) was modeled using the crystal structure of BAI3 (pdb 4DLO) as a template. Synaptamide is shown with a space-filling (**a**, **c**) or stick and ball representation (**b**).

The synaptamide-induced changes in the cross-linking profile observed in the GAIN domain prompted us to model the interaction of the GAIN domain with synaptamide. Analysis of the structural model of the GAIN domain revealed that a long channel comprises a polar/exposed region and a hydrophobic core at the interface between GAIN subdomains A and B and is a potential pocket for synaptamide binding. We therefore docked synaptamide into the pocket using a step-wised ensemble docking approach, taking into account protein flexibility and ligand-induced conformational changes^36^. The predicted binding model shows that synaptamide fits well into the pocket, with the ethanolamine headgroup pointing up to a polar region at the top, while the long fatty acid acyl chain extends down to the hydrophobic channel of the pocket (Fig. 4a). The hydroxyl moiety of ethanolamine head is found to preferably bind with N512 located at the beginning of the loop between β-strands 7 and 8 (subdomain B) and P476 by forming two hydrogen-bonding interactions, which further positions the carbonyl oxygen to form an additional H-bond with Q511 (Fig. 4b). The polar hydroxyl end is bound to a solvent-exposed region at the top of the pocket suggesting that a bulky group at this site may be further tolerated. This is consistent with biotinylated- or bodipy-labeled synaptamide analogs, previously shown to retain synaptamide-like bioactivity^20^, also fitting well in the pocket (Supplementary Fig. 6). The model also predicts a hydrogen bonding between Y513 and the C11–C12 double-bond of the fatty acyl chain. In an alternative model, interactions involving R365 and H363 located at the loop between helices 3 and 4 in subdomain A are predicted (Fig. 4b).

According to the model, K398 is located near α-helix 6 of subdomain A and K438 at β-strand 2 of subdomain B and these two residues span the predicted binding pocket (Fig. 4c). It is expected, therefore, that their spatial proximity and inter-subdomain cross-linking would be altered by ligand binding, which matches the experimental observations. Together, the experimental and modeling data provide compelling evidence supporting a synaptamide-binding pocket in the subdomain interface of the GAIN domain.

Because the GAIN domain is conserved in the aGPCR class, we questioned whether synaptamide would also bind to other aGPCRs. To address this issue, we aligned the sequence of GPR110 with other aGPCRs including the closely-related GPR116 (ADGRF5). However, the ligand-binding residues Q511, N512, and Y513 are not conserved in the GAIN domains of other aGPCRs (Supplementary Figs. 7, 8). Modeling of the GAIN domain of GPR116 predicts a markedly weaker binding affinity to synaptamide compared to GPR110 (Supplementary Fig. 9). We also attempted to dock synaptamide to the crystal structure of BAI3 but synaptamide does not fit into that GAIN domain structure. Residue R793 in the hydrophobic groove, in particular, points into the pocket, thus blocking the binding of synaptamide (Supplementary Fig. 9). In addition, the hydrophobic groove is found to be shorter and lacks the hydrophobicity for interacting with fatty acyl chain of DHA, while the head binding area above the groove is much less polar compared to the case with GPR110 (Supplementary Fig. 10). These data indicate that binding of synaptamide by GPR110 is specific despite the overall similarity in the conserved GAIN domain across the entire aGPCR family.

### Identification of ligand interacting residues in GAIN domain

**Fig. 5:**
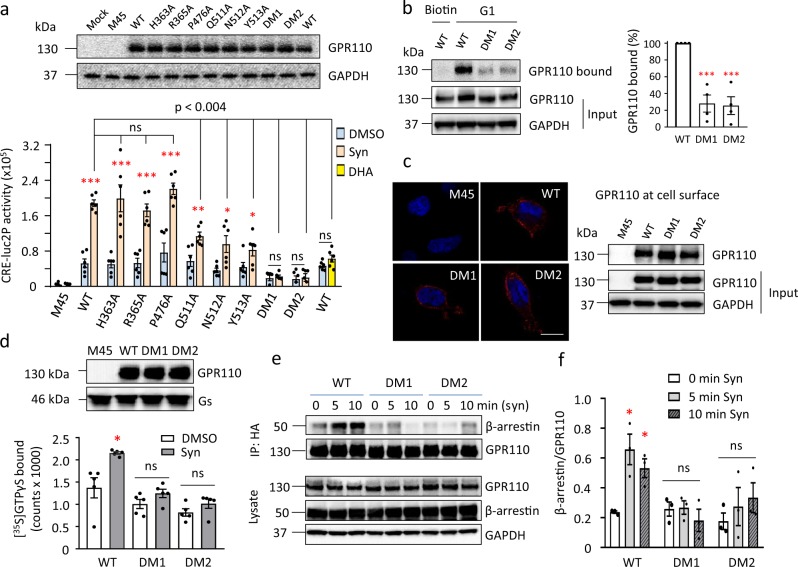
Effect of the GAIN domain mutations on the GPR110 signaling and ligand binding. **a** Synaptamide-induced cAMP production affected by mutations. GPR110 mutants were overexpressed in CRE-luc2P HEK293 cells which contain a luciferase gene (luc2P) as the cAMP sensor. The Western blotting shows comparable expression of mutants. M45, control vector. **b** Binding of GPR110 to biotinylated analogue of synaptamide (G1) impaired by mutations of Q511A/N512A (DM1) and N512A/Y513A (DM2). Lysates from transfected HEK cells were treated with G1 followed by incubation with streptavidin beads. The G1-bound GPR110 was detected by Western blotting using anti-HA antibody, and the quantitative result is shown as the band intensity ratio (bound vs total expression) normalized to that of the WT. **c** Surface expression of GPR110 unaltered by the mutations. Transfected HEK cells were subjected to non-permeable staining or labeled with a membrane-impermeable biotinylated cross-linker. The biotinylated cell surface proteins were enriched with streptavidin beads and subjected to Western blotting. Scale bar: 10 μM. **d** [^35^S]GTPγS binding assay. Western blotting shows a similar level of GPR110 (WT or mutants) and Gs in the membrane fractions used in the assay. **e**, **f** Western blots (**e**) quantitation (**f**) of co-immunoprecipitation experiments in HEK cells overexpressing GPR110 and β-arrestin. Statistical analysis was performed using Student’s *t*-test. *p < 0.05; **p < 0.01; ***p < 0.001. Data are means ± SEM of biologically independent samples; *n* = 6 (**a**), *n* = 4 (**b**), *n* = 5 (**d**), *n* = 3 (**f**). Syn synaptamide.

Based on the model, we performed site-directed mutagenesis of potential binding residues in GPR110 GAIN domain and evaluated synaptamide-induced cAMP production. In addition to residues predicted to participate in hydrogen-bonding interactions, H363 and R365, which potentially interact with the headgroup of synaptamide, were selected for the mutagenesis study. However, neither the mutation of H363A or R365A had an impact on bioactivity. The P476A mutant also had little effect on cAMP activity as expected owing to its weak interaction through the proline backbone oxygen. In contrast, mutations at Q511A, N512A and Y513A significantly impaired synaptamide-induced cAMP production compared to the WT, while double mutations of Q511A/N512A and N512A/Y513A completely abolished any synaptamide effect (Fig. 5a, Supplementary Fig. 1, Supplementary Data 1). Moreover, these mutations significantly impaired the binding of synaptamide to the receptor (Fig. 5b, Supplementary Fig. 1, Supplementary Fig. 11, Supplementary Data 1). Computational alanine scanning indicates that these mutations did not alter the structural arrangement of the receptor, which was further supported by an unaltered expression level of the mutants at the plasma membrane (Fig. 5c, Supplementary Fig. 1, Supplementary Fig. 11). Therefore, the effects of these mutations appear to be due to a loss of hydrogen-bonding with the ligand. These data reveal that specific interactions of residues Q511, N512, and Y513 in the GAIN domain with the polar ethanolamine headgroup, carbonyl group and the hydrophobic DHA chain of synaptamide are important contributors to the binding of synaptamide with GPR110. Each of these three residues in GPR110 seems to be necessary for full activation by synaptamide. Increased cAMP production was not observed with DHA, the parent compound of synaptamide (Fig. 5a)^20^, indicating that fatty acyl chain interaction with Q511 and Y513 are not sufficient and that the ethanolamine headgroup is critical to strengthen and stabilize binding, presumably through interaction with N512. In fact, computational modeling indicates that DHA displays lower binding affinity to the GAIN domain when evaluated using the GBVI/WSA score^37^ (Supplementary Fig. 12). Furthermore, when the DHA chain in synaptamide structure is changed to other fatty acids such as oleic, palmitic or arachidonic acid, a decrease in the predicted binding affinity is also observed (Supplementary Fig. 12), indicating that there is a specific binding between the GAIN domain of GPR110 and the endogenous lipid synaptamide.

To provide direct evidence that the ligand-induced conformational change of the intracellular regions of GPR110 is coupled to G-protein activation, we performed [^35^S]GTPγS binding assay^4,38^ using the membranes from the HEK cells overexpressing GPR110 WT or mutants along with Gs protein (Supplementary Fig. 13). While synaptamide produced activation of Gs protein in WT GPR110, GTP binding was not observed with the double mutants, Q511A/N512A and N512A/Y513A (Fig. 5d, Supplementary Fig. 1, Supplementary Data 1), where the ligand binding was impaired (Fig. 5b). These data strongly support that TM6 and the intracellular region of the receptor where cross-linking changes were observed (Table 1) were indeed involve in G-protein interactions.

Since the intracellular *C*-terminal tail of activated GPCRs is phosphorylated and interacts with β-arrestin^16,39^, we also accessed β-arrestin binding activity. After activation with synaptamide, β-arrestin showed robust binding to WT GPR110. However, co-immnoprecipitatation of β-arrestin was no longer observed for GPR110 double mutants (Fig. 5e, f, Supplementary Fig. 1, Supplementary Data 1). The β-arrestin recruitment data confirmed that GPR110 activation occurred upon binding to synaptamide.

### Modeling the TM and intracellular domains

**Fig. 6:**
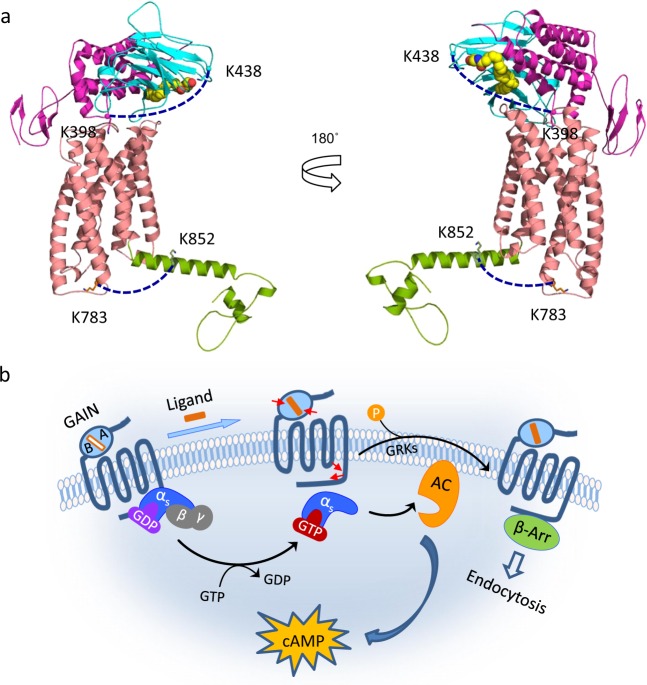
A molecular mechanism of GPR110 activation. **a** Ligand binding to the GAIN domain of the extracellular region induces conformational changes in GPR110. The conformational changes were deduced by monitoring the inter-subdomain cross-linking in the GAIN domain (K398-K438) and the intracellularly cross-linking between K783 of the TM6 and K852 of the *C*-terminal region (green). The K398-K438 and K783-K852 cross-links are indicated by dotted lines. The 7TM and *C*-terminal region of GPR110 were modeled with the structures of corticotropin-releasing factor receptor 1 (pdb 4K5Y) and glucagon receptor (pdb 4L6R). The GAIN domain was modeled using the crystal structure of BAI3 (pdb 4DLO). Synaptamide, the first small-molecule endogenous ligand for an aGPCR, is presented by a space-filling model. **b** A cartoon representation of GPR110 activation. Specific binding of synaptamide to the GAIN domain activates GPR110 through conformational changes (depicted by red arrows) within the GAIN domain and in the intracellular regions involving TM6 and the *C*-terminal tail. The intracellular conformational change results in Gs protein activation and β-arrestin recruitment. The ligand, and the binding pocket which is located at the interface between the subdomain A and subdomain B of the GAIN domain, are depicted with filled (orange) and open rectangles, respectively. AC Adenylyl cyclase, β-arr β-arrestin, GRKs G-protein-coupled receptor kinases.

The 7TM and the intracellular domains of GPR110 were modeled with the crystal structures of two class B (or secretin family) GPCRs, corticotropin-releasing factor receptor 1 (pdb 4K5Y) and glucagon receptor (pdb 4L6R). Despite having only ~20% sequence identity for the TM domains, the model is in agreement with the secondary structure predicted by PSIPRED^28^ although the latter shows shorter helices in general (Supplementary Data 3, Supplementary Fig. 14). The inter-domain cross-link of K783-K852 is depicted in the model shown in Fig. 6a. The predicted C_α_-C_α_ distance of K783 and K852 (24.4 Å) is within the expected distance constraint imposed by DSS. Interestingly, the model reveals that intracellular loop 3 between TM5 and TM6 is very short and that K783 is located at the cytoplasmic end of the helix 6. Of note, these intracellular regions are thought to interact with the G protein for downstream signaling^29,40^. In this regard, the inter-domain conformational change in the intracellular regions detected by K783-K852 cross-linking provides solid evidence at the molecular level that synaptamide binding to the extracellular region of GPR110 induces the conformational changes of the receptor that activate G-protein interactions and downstream signal transduction including cAMP production and β-arrestin recruitment (Fig. 6b).

## Discussion

It has been recognized that GAIN-mediated autoproteolysis plays an important role in aGPCR signaling, which leads to activation via Stachel-dependent mechanism. A Gq-dependent GPR110 activation based on such machinery has been demonstrated in vitro with synthetic peptide ligands containing the Satchel sequence at high μM concentration^4^. Interestingly, the Gs-dependent GPR110 signaling triggered by synaptamide at nanomolar concentrations does not require the self-cleavage of the GAIN domain nor the sequence integrity of the tethered agonist, suggesting a distinctively different mode of activation^20^. In this study, we set out to investigate the molecular mechanism for this ligand-induced activation in living cells by probing in-cell conformational changes of GPR110 using chemical cross-linking and mass spectrometry in combination with computational modeling and mutagenesis. We demonstrate that synaptamide specifically interacts with the GAIN domain and induces conformational changes in the extracellular GAIN domain as well as the intracellular regions where G-protein and β-arrestin interactions presumably occur in living cells. These findings reveal an aGPCR activation mechanism mediated by specific GAIN domain-ligand interaction away from the TM region where the ligand binding often occurs for many GPCRs (Fig. 6b).

Although the GAIN domain is highly conserved in aGPCRs, its function other than autoproteolysis is not clear. Recent studies have revealed that monobodies can interact with the ECR and regulate signaling of aGPCR GPR56^17^. A couple of these synthetic protein ligands have been shown to interact with full-length ECR at the interface of the GAIN (subdomain A) and the Pentraxin/Laminin/neurexin/sex-hormone-binding-globulin-Like (PLL) domains, but not to the isolated GAIN or PLL domain. To date, functional ligands specifically targeting the GAIN domain have not been demonstrated for the aGPCR class. The interaction of GPR110 GAIN domain with its endogenous ligand was deduced from our data of the change in the cross-linking of K398 (subdomain A)-K438 (subdomain B) in the GAIN domain (Table 1) and strongly supported by structure modeling, GAIN domain site-directed mutagenesis, and downstream bioactivity assays (Fig. 5). Our data also reveal that synaptamide interacts with a long channel that contains a polar region followed by a hydrophobic groove at the subdomain interface within the GAIN domain. While the unique hydrophobic groove accommodates the DHA structure of synaptamide, Q511, N512, and Y513 of the polar region interact with the ethanolamine headgroup, carboxy group and fatty acid chain of synaptamide (Fig. 4b), together accounting for the specificity and efficacy of the ligand for binding and downstream signaling. These binding characteristics provide the basis for GPR110-dependent biological effects triggered uniquely by synaptamide, among other ethanolamide analogs with different fatty acyl chains and DHA, which lacks the proper headgroup^20^. The data are also consistent with our previous finding that synaptamide binds to the *N*-terminal fragment of GPR110^20^.

Currently available X-ray crystallographic data suggest that there are significant differences in GAIN domains among aGPCRs, despite similarities of their secondary structure^2^. The GAIN domain of GPR110 differs from that of the BAI3, particularly in the interface between the subdomains. For example, the hydrophobic groove in the interface of BAI3 is shorter with less hydrophobicity making it difficult to accommodate a long-chain polyunsaturated fatty acyl chain such as DHA, and the residue R793 in the groove interferes with synaptamide binding (Supplementary Figs. 9, 10). Even in the closely-related GPR116 structure, the synaptamide binding module consisting of Q511, Y513, and N512 is not conserved (Supplementary Fig. 7). On the other hand, the subdomain interface of BAI3 and other aGPCRs may be tailored to accommodate other endogenous ligands. In this regard, our data provide a basis for modeling potential new ligands that target the GAIN domain and help to ultimately uncovering the physiological functions of aGPCRs, particularly for those remaining orphan receptors.

The structure of aGPCRs exhibits diversity and complexity. In addition to the GAIN domian, a total of 18 different domains are identified in the extracellular region among the 33 members of this class. Unlike other members, a SEA domain (AA ~148–256), which is also present in GPR116, is followed directly by the GAIN domain in GPR110. Although it has been well documented that the SEA domain of adhesive proteins contains a proteolytic cleavage site and assists or regulates binding to glycans, the function of the SEA domain is largely unknown^41^. Solution or crystal structure of the SEA domain for cell surface receptors such as MUC16 (pdb 2E7V) or transmembrane protease has been reported^42^. Unfortunately, the GPR110 SEA domain shares little sequence homology with these proteins, making it impractical to model. Nevertheless, our cross-linking approach reveals several through-space cross-links throughout this domain, including K151-K187, K151-K254, K187-K240, and K240-K254. Of note, the K151-K254 cross-link provides the information regarding the proximity (~24 Å) of the *N*-terminus and *C*-terminus of the SEA domain (Supplementary Fig. 15). The observation that the cross-linking profile in this domain was not altered by synaptamide suggests that the SEA domain may not directly participate in the ligand-binding.

Of note, chemical cross-linking efforts have long been focused on in vitro structural analysis of purified proteins or isolated protein complexes^43–45^. Although chemical cross-linking in living cells has been successfully employed for probing topological information of protein complexes and/or protein-protein interactions^23,24,46,47^, the 3D structural elucidation of native proteins by chemical cross-linking within a cellular context, particularly for GPCRs, has been extremely challenging and thus rarely been reported. To our knowledge, our data demonstrate for the first time the conformational analysis of a GPCR by intramolecular cross-linking in living cells.

We have previously demonstrated that synaptamide-mediated GPR110 signaling involves the activation of Gs protein^20^. The β2- adrenergic receptor (β2-AR, belongs to class A GPCR), one of the best-studied GPCRs, also couples to Gs. The crystal structure of this receptor has indicated that TM5 and TM6 interact with the amino- and carboxyl-terminal α-helices of Gs^29^. The most significant structural change of β_2_-AR was seen for the TM6 that showed a 14 Å outward movement when it was complexed with Gs and activated^29^. The cross-linking data shown in this study indicate that synaptamide binding induces a conformational change in the intracellular regions involving K783 at the cytoplasmic end of TM6 and K852 in the *C*-terminal region. This conformational change seems to be compatible with the activation scenario of β2-AR. To our knowledge, this is the first demonstration of the ligand-induced conformational changes during aGPCR activation. The specific ligand binding to GPR110 GAIN domain influences the 7TM and intracellular domain conformations, thereby transmitting G-protein signaling and downstream actions including β-arrestin recruitment (Fig. 6).

In conclusion, we present here 3D structural changes of GPR110 induced by its endogenous ligand, synaptamide, in living cells by using chemical cross-linking and mass spectrometry. Combined with computational modeling and mutagenesis, we reveal the specific binding site of synaptamide in the GAIN domain. The interaction of synaptamide with the extracellular GAIN domain causes an intracellular conformational change and triggers G-protein activation and downstream signaling. The ligand-induced and GAIN domain-targeted mechanism provides a framework for understanding physiologically-relevant molecular functions of aGPCRs. Our findings may also facilitate the development of synaptamide-like ligands for this emerging class of GPCRs and to develop GAIN domain-targeting agents as a potential therapeutic strategy for aGPCR-related dysfunction.

## Methods

### Materials

Succinimidyl suberate (DSS), HA-peptide, trifluoroacetic acid, protease inhibitor cocktail without EDTA, and ECL western blotting substrates, were obtained from ThermoFisher Scientific. PBS (pH 7.4, 795 mg/L Na_2_HPO_4_, 144 mg/L KH_2_PO_4_, 9000 mg/mL NaCl, without calcium and magnesium), Dynabeads protein G, Dynabeads M-280 streptavidin and MOPS SDS running buffer, were purchased from Invitrogen. Tris-HCl (pH 7.4), goat serum, anti-rabbit or anti-mouse IgG peroxidase secondary antibody, Triton X-100, DTT, and iodoacetamide were purchased from Sigma. Synaptamide, biotinylated- and bodipy-synaptamide were prepared by NIAAA/NIH or NCATS/NIH. β-arrestin 2 (variant 1) DNA was obtain from Origene.

### Cell culture

HEK293 (American Type Culture Collection) or GloResponse^TM^ CRE-luc2P HEK293 reporter cell line (Promega) were cultured in DMEM (Invitrogen) with 10% fetal bovine serum (FBS, Invitrogen) in humidified CO_2_ incubator. Transfection of GPR110-HA (WT or mutants), with or without Gs or β-arrestin, was performed using Lipofectamin 2000 (Invitrogen) according to the manufacturer’s instructions.

### Plasmid constructs

*C*-terminal HA-tagged human GPR110 construct containing full-length open reading frame sequence of human GPR110 (NM_153840.2) (GPR110-HA) and the control empty vector M45 were obtained from GeneCopoeia (Rockville, MD). Point mutations were performed with QuickChange II site-directed mutagenesis kit (Agilent) according to manufacturer’s instructions.

### Chemical cross-linking in living cells

HEK293 cells grown in a 15-cm dish were transfected with human GPR110-HA for 24 h. After removing the medium, cells were washed one time with PBS (pH 7.4) and resuspended in 5 mL of PBS. Cells were stimulated with 10 nM synaptamide or oleoylethanolamine (OEA) control or DMSO vesicle for 10 min followed by incubation with 1 mM DSS (i.e., adding 25 µL of freshly made 200 mM DSS in DMSO, final concentration of DMSO was 0.5%) for 30 min at room temperature (25 °C). The cross-linking reaction was quenched by addition of 150 µL of 1 M Tris-HCl (pH 7.4). The cells were lifted by gently scraping and the cell suspension was transferred to a 15-mL conical tube. Cells were pelleted by centrifugation at 1000 rpm for 10 min at 4 °C, and then lysed in 1.5 mL lysis buffer (Cell Signaling Technology) on ice for 40 min with vortexing at 5-min intervals. Cell debris was removed by centrifugation at 15,000 rcf for 15 min and the supernatants were subjected to immunopurification.

### Immunopurification of GPR110

The cell lysate was incubated with 40 µL of HA antibody (Santa Cruz Biotech., Cat #:7392) overnight followed by additional 4-h incubation with 40 µL Dynabeads protein G beads at 4 °C. After washing of the beads five times with lysis buffer, the immunoprecipitated GPR110 was eluted by incubating with 50 µL of lysis buffer containing 1 mg/mL HA peptide (ThermoFisher Scientific) at 30 °C for 20 min.

### SDS-PAGE and in-gel digestion

Immunopurified proteins were mixed with 4× lithium dodecyl sulfate (LDS) sample buffer (Life Technologies, Cat #: B0007) at 37 °C for 30 min. Samples were loaded onto Bolt 4–12% Bis-Tris gels (Life Technologies). Electrophoresis was carried out at a constant voltage of 100 V using MOPS SDS running buffer for approximate 60 min. Proteins including protein standards (Bio-Rad, Cat:161-0374) were stained with Coomassie blue (SimplyBlue SafeStain, Life Technologies). The monomeric protein band (100–150 kDa) was excised for reduction/alkylation and tryptic digestion^48^. Briefly, the gel was diced into small pieces (1–2 mm), distained with 25 mM NH_4_HCO_3_ in 50% ACN, dried by vacuum centrifugation, and subjected to in-gel reduction and alkylation with 10 mM DTT (56 °C for 60 min) and 50 mM iodoacetamide (25 °C for 45 min in dark), respectively. After sequential washing with 25 mM NH_4_HCO_3_, 25 mM NH_4_HCO_3_/50%ACN (twice), and 100% ACN, gel pieces were dried and rehydrated with 12.5 ng/mL trypsin (Promega) solution in 25 mM NH_4_HCO_3_ on ice for 30 min. The digestion was continued at 37 °C overnight. The tryptic peptides were extracted with 5% formic acid/50% ACN, concentrated by vacuum centrifugation, and desalted using C-18 ziptip (Millipore).

### Nano-HPLC MS/MS analysis

Nano-LC-ESI-MS/MS was performed on an LTQ-Orbitrap XL mass spectrometer (Thermo Scientific) equipped with an Eksigent nanoLC 1D system^48^. The mobile phases consisted of 0.1% formic acid (solvent A) and 0.1% formic acid in 95% ACN (solvent B). Peptide samples were loaded onto a C18 trap column (Sciex, Cat.#: 5016752) and separated by a 15-cm IntegraFrit column (ProteoPep^TM^, New Objective) at a flow rate of 300 nL/min with a gradient from 5–40% solvent B in 150 min. LC eluent was sprayed into the MS instrument with a glass emitter tip (PicoTip, New Objective) using a spray voltage of 2.0 kV in positive-ion mode. Full scan spectra from *m/z* 300–1700 at resolution of 60,000 were acquired in the Orbitrap. Ten data-dependent MS/MS spectra of most intense ions were acquired in the LTQ-XL ion trap using CID with a normalized energy of 35. Dynamic exclusion for the already fragmented precursor ions was used with the following parameters: exclusion time 180 s, repeat count 1, repeat duration 30 s, exclusion mass width 10 ppm, and exclusion size 500. Singly charged species were excluded from fragmentation.

### Protein identification

The acquired MS/MS data were searched against the NCBInr human database with Mascot (v2.3.2, Matrix Science) using Mascot Distiller (2.3.2.0) as the data input filter to generate peak lists. Search parameters were set as follows: enzyme, trypsin; precursor ion mass tolerance, 10 ppm; fragment ion mass tolerance, 0.3 Da; maximum missed cleavages allowed 2; carbamidomethyl of cysteine residues for fixed modification; oxidation of methionine and addition of 156. 07864 Da on lysine or *N*-terminal (end-capping modification) for variable modification. The criteria used to filter results included 1% false positive threshold and expect value of less than 0.05 for significant peptide matches. The expect score was calculated using the homology threshold or the significance threshold as per a standard Mascot protein family report.

### Identification of cross-linked peptides

The cross-linked lysine pairs were identified by xQuest software (http://prottools.ethz.ch/orinner/public/htdocs/xquest/xquest_review.html) followed by manual verification of the MS/MS spectrum^49^. Briefly, the MS/MS data were converted to mgf file by Proteome Discovery (version 1.4) and further formatted in accordance with xQuest’s requirements. The parameters used in the search against a database containing human GPR110 sequence were as follows: enzyme, trypsin kr[^P]; cross-link mass-shift, 138.06808 Da; monolink mass-shift, 156.07864 Da; reactive amino acid, K; Ionization mode, ESI; fixed modification, C:57.02146 Da; MS1 tolerance, 10 ppm; MS2 tolerance [*m/z*], 0.3. Only the cross-links with high quality MS/MS inspected manually were reported in the present study.

### Label-free quantitation

The acquired spectra from biological triplicate were loaded (Thermo raw files) into the Progenesis QI for Proteomics software (version 1.05156.29278) for label-free quantitation. Automatic alignment of chromatograms and automatic peak-picking settings were used to process the data. Features with charge of 1 and charge >7 were filtered out for the analysis. Normalization to all proteins was performed based on the assumption that a significant number of features were unaffected across different sample runs. Peptide and protein identifications were performed using Mascot search engine via Mascot Distiller. A Mascot score corresponding to a *p*-value of 0.05 was set as a threshold for peptide identifications. Proteins were quantitated from nonconflicting features. Results of the peptide and protein measurements were exported as Excel files and the cross-linked peptides of GPR110 were further normalized to the GPR110 protein level determined by the Progenesis software. Significant changes of the cross-linked peptides were based on *p* ≤ 0.05 (unpair Student’s *t*-test) and 1.5 fold-change (synatamide-treated vs control) from biological triplicate.

### Homology modeling and docking

The 3D structure of GPR110 was generated using the I-TASSER program^50,51^. The GAIN domain (251–580) was modeled using the crystal structure of brain angiogenesis inhibitor 3 (BAI3, pdb 4DLO) as a template. The best model generated from I-TASSER (C-score = 1.13 and TM-score = 0.87) was selected for subsequent modeling and docking analysis. The 7TM and the intracellular region of GPR110 were modeled using the templates of corticotropin-releasing factor receptor 1 (pdb 4K5Y) and glucagon receptor (pdb 4L6R). The best model with C-score 0.9 and TM-score 0.84 was selected for the study. A two-domain model of GPR110 was generated by manually placing the models of the GAIN domain and the 7TM/intracellular region together. A short minimization was performed to avoid steric clashes at the protein-protein binding interface. Docking studies of synaptamide and analogs to the GAIN domain of GPR110 were performed using the MOE program^52^. The induced fit protocol was used for ligand docking and the binding affinity was evaluated using the GBVI/WSA score^52^. MD simulations were performed for the predicted GAIN/synaptamide-binding complex using Amber 18^53^.

### Western blot analysis

Samples were electrophoresed in 4–12% Bis-Tris gels at 150 V using MOPS SDS running buffer. Proteins were transferred to a PVDF membrane (Bio-Rad, cat.#: 1704156) at 25 V for 25 min using a Bio-Rad Trans-Blot Turbo transfer system. The membrane was blocked with 5% milk in TBS containing 0.1% Tween 20 (TBS-T) at room temperature for 1 h. Blots were incubated with primary antibody at 4 °C overnight, washed three times with TBS-T, then incubated with peroxidase-conjugated secondary antibody for 1 h at room temperature. After washing three times with TBS-T, blots were incubated with enhanced chemiluminescent (ECL) substrates containing 90% of substrate mix 1 (cat.#: 34080) and 10% substrate mix 2 (cat.#: 34094, ThermoFisher Scientific) for 5 min, and imaged with a Kodak Gel Logic 440 Imaging system^48^. Band intensity was quantitated using Kodak 1D imaging analysis software.

### Binding of biotinylated synaptamide to GPR110

HEK293 cells overexpressing human GPR110-HA WT or mutants were lysed in PBS containing 0.5% Triton X-100 and protease inhibitors. The lysate was treated with 1 µM biotinylated synaptamide (G1) or biotin at 25 °C for 30 min followed by incubation with Dynabeads M-280 streptavidin at 25 °C for 30 min. The beads were washed in the lysis buffer with mild shaking for 10 min followed by three quick washes without shaking at room temperature. The beads were incubated with 2X LDS sample buffer (Life Technologies) at 37 °C for 30 min. The G1-bound GPR110 was then detected by western blotting using anti-HA antibody (Santa Cruz Biotech., Cat.# sc-7392, 1/200 ratio) and anti-mouse peroxidase-conjugated secondary antibody (Sigma, Cat.# A4416, 1/500 ratio). Background level obtained from biotin control sample was subtracted for quantitation purpose. Statistical analysis was performed using Student’s *t*-test.

### Luciferase assay for CRE (cAMP response element) activity

The GloResponse^TM^ CRE-luc2P HEK293 cells containing a luciferase gene (luc2P) (Promega) were seeded in 24-well plates at 2.5 × 10^6^ cells/well, transfected with WT GPR110-HA, GPR110-HA mutants, or empty vector M45 (GeneCopoeia) for 24 h, and stimulated with 10 nM synaptamide (or DHA) in DMEM containing 0.01% BSA and 40 μM vitamin E. Experiments without transfection (Mock) were performed in parallel to subtract the background noise. After 16 h, the cells were measured for the CRE activity using the Dual-Glo® Luciferase assay kit (Promega) following the manufacturer’s instructions. Background level obtained from Mock samples was subtracted. Statistical analysis was performed using unpaired Student’s *t*-test.

### Immunocytochemistry

To visualize GPR110 membrane localization, HEK293 cells were cultured and transfected with GPR110-HA for 48 h on poly-lysine coated glass cover slips. The cells were washed in PBS at room temperature, fixed in 2% paraformaldehyde (PFA) at room temperature for 10 min, and washed three times with PBS at 4 °C. The nonspecific binding was blocked at 4 °C with detergent-free blocking buffer containing 10% goat serum and 1% BSA. The cells were incubated with anti-GPR110 antibody (1:200 dilution, Lifespan Biosciences, Cat.# LS-A2021) overnight at 4 °C, washed three times for 10 min with PBS, and incubated with Alexa 555-conjugated goat anti-rabbit (1:500 dilution, ThermoFisher Scientific) and DAPI for 1 h at room temperature. After washed three times with PBS a cover slip was attached to the cells with fluoromount. Six fields (10–15 cells per field) were randomly sampled per slide. The mounted cells were observed under a LSM700 Confocal microscopy (Zeiss, Germany).

### Cell surface biotinylation

HEK293 cells were transfected with WT GPR110-HA, or GPR110-HA mutants or empty vector (M45) for 24 h. The cells were washed with ice-cold PBS and incubated with 0.5 mg/mL Sulfo-NHS Biotin (ThermoFisher Scientific) in PBS for 1 h in 4 °C^54^. After washing four times with PBS containing 100 mM glycine, the cells were lysed with lysis buffer (Cell Signaling Tech. Cat.# 9823) for 30 min on ice. Cell debris was cleared by centrifugation (12,000 rpm) and soluble cell lysates were incubated with 50 μl Dynabeads M-280 streptavidin beads for 1 h at 4 °C. Beads were washed three times with lysis buffer and then incubated with 50 μl 2× LDS in 4 °C for 30 min. Biotinylated GPR110 were detected by Western blotting using HA-antibody.

### [^35^S]GTPγS binding assay

HEK293 cells grown in 15-cm dishes were transfected with Gs (cDNA Resource Center) and GPR110-HA (WT or mutants) or empty vector M45 for 24 h. Membrane preparation and [^35^S]GTPγS binding were performed according to a protocol published previously^38^, except that no GDP was included in the binding buffer^4,38^. Background values of the membrane obtained from overexpressing empty vector M45 and Gs were subtracted.

### β-Arrestin binding assay

HEK293 cells were transfected with β-arrestin 2 and GPR110-HA (WT or mutants) for 24 h. After replacing the medium with 5 mL PBS, cells were treated with 2.5 μL of 20 μM synaptamide (final concentration of synaptamide was 10 nM) or DMSO for 5 or 10 min. Cells were lysed with lysis buffer containing 1% Triton (Cell Signaling Tech, Cat.# 9803) for 45 min on ice. Cell debris were cleared by centrifugation and soluble cell lysates were subjected to immunoprecipitation with HA antibody as described in the “Immunopurification of GPR110” section. GPR110 and β-arrestin were detected and quantified by western blotting using anti-HA antibody and anti- β-arrestin antibody (Cell Signaling Tech. Cat.# 3857) respectively.

### Multiple sequence alignment

Multiple sequence alignment for GPR110, GPR111, GPR115, GPR116, and GPR113 was performed using CLUSTALW (https://www.genome.jp/tools-bin/clustalw).

### Statistics and Reproducibility

Significance was determined by Two-tailed Student’s *t*-test using Excel or analyzed by one-way ANOVA and Bonferroni post-hoc test using GraphPadPrism 8.0 software. *P*-values < 0.05 were considered significant. Data are presented as mean ± SEM (standard error of the mean) of at least three independent experiments.

### Reporting summary

Further information on research design is available in the Nature Research Reporting Summary linked to this article.

## Supplementary information

Supplementary Information

Description of Additional Supplementary Files

Supplementary Data 1

Supplementary Data 2

Supplementary Data 3

Reporting Summary

## Data Availability

Source data underlying the graphs are available in Supplementary Data 1. Raw mass spectrometric data have been deposited to the ProteomeXchange Consortium via the PRIDE^55^ partner repository with the dataset identifier PXD017128. All other data supporting the findings of this study are available in the main and Supplementary files.
