# Supplementary material for: Synaptamide activates the adhesion GPCR GPR110 (ADGRF1) through GAIN domain binding
Source: Commun Biol. 2020 Mar 6;3:109. doi: 10.1038/s42003-020-0831-6 (PMC7060178; doi:10.1038/s42003-020-0831-6)
Supplement: Supplementary file 1 — Supplementary Information [file 42003_2020_831_MOESM1_ESM.pdf]

Supplementary Figure 1. Uncropped blot/gel images

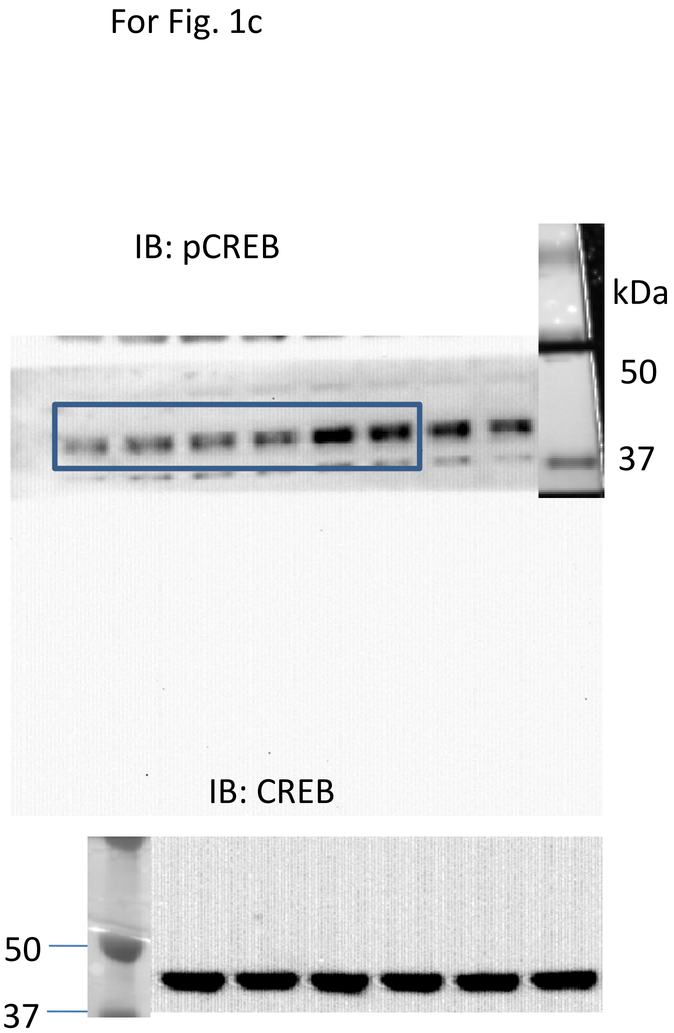

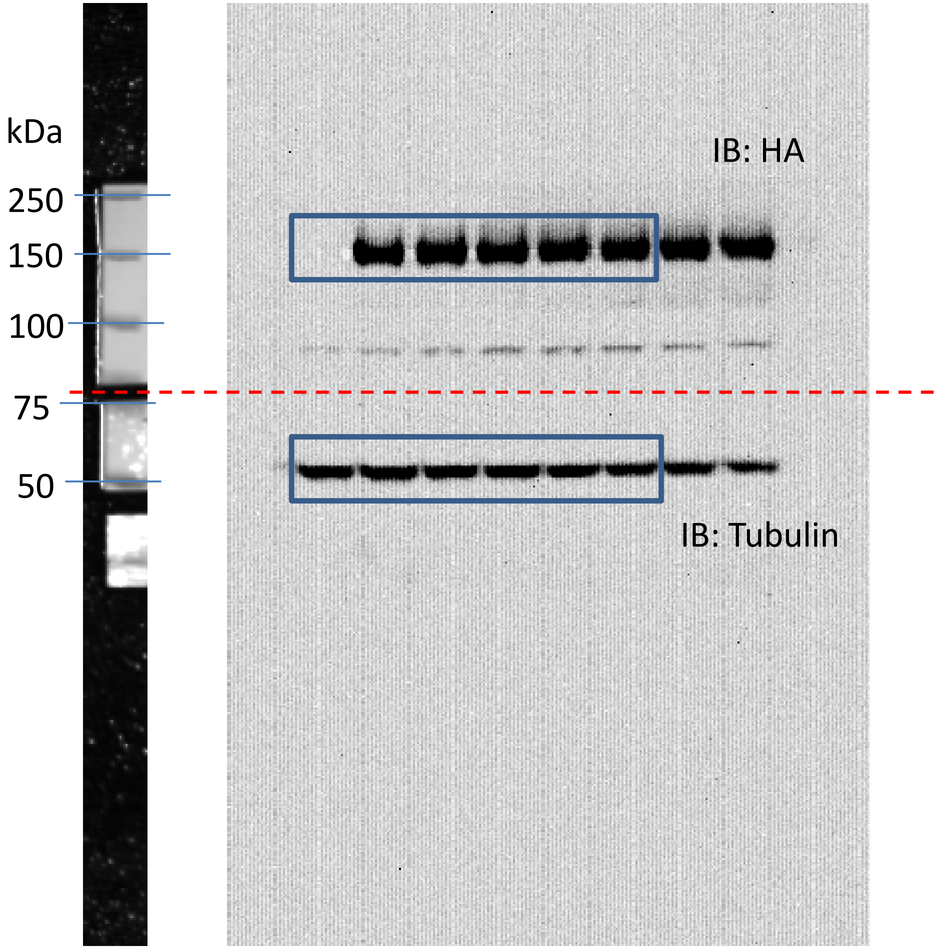

For Fig. 4a

GPR110

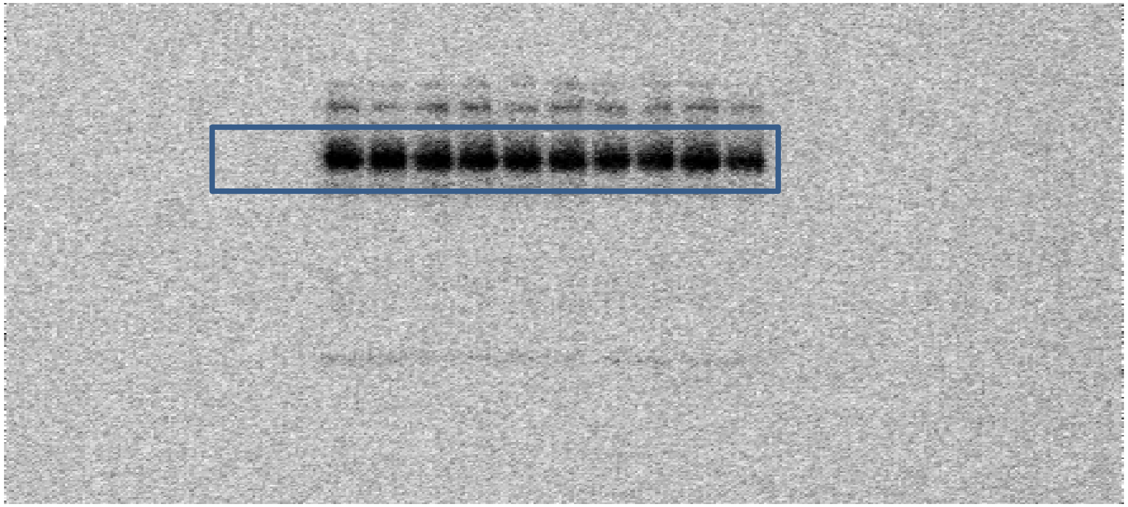

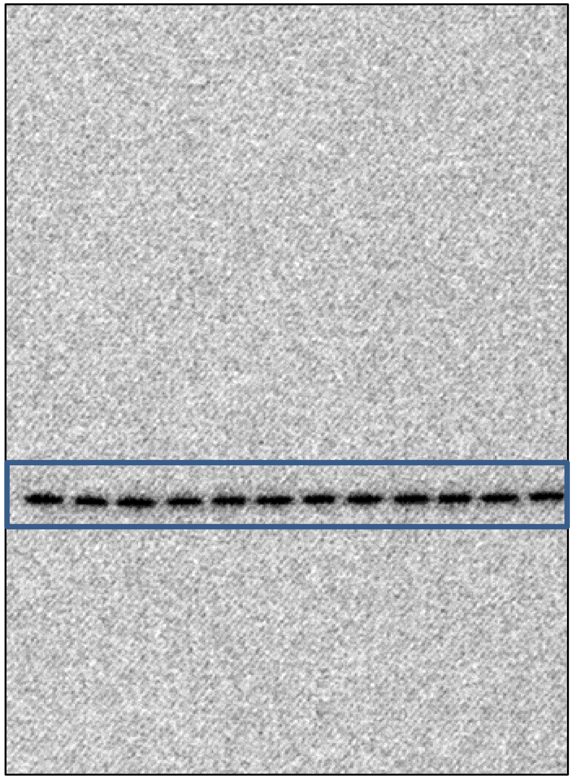

GAPDH

For Fig. 4b

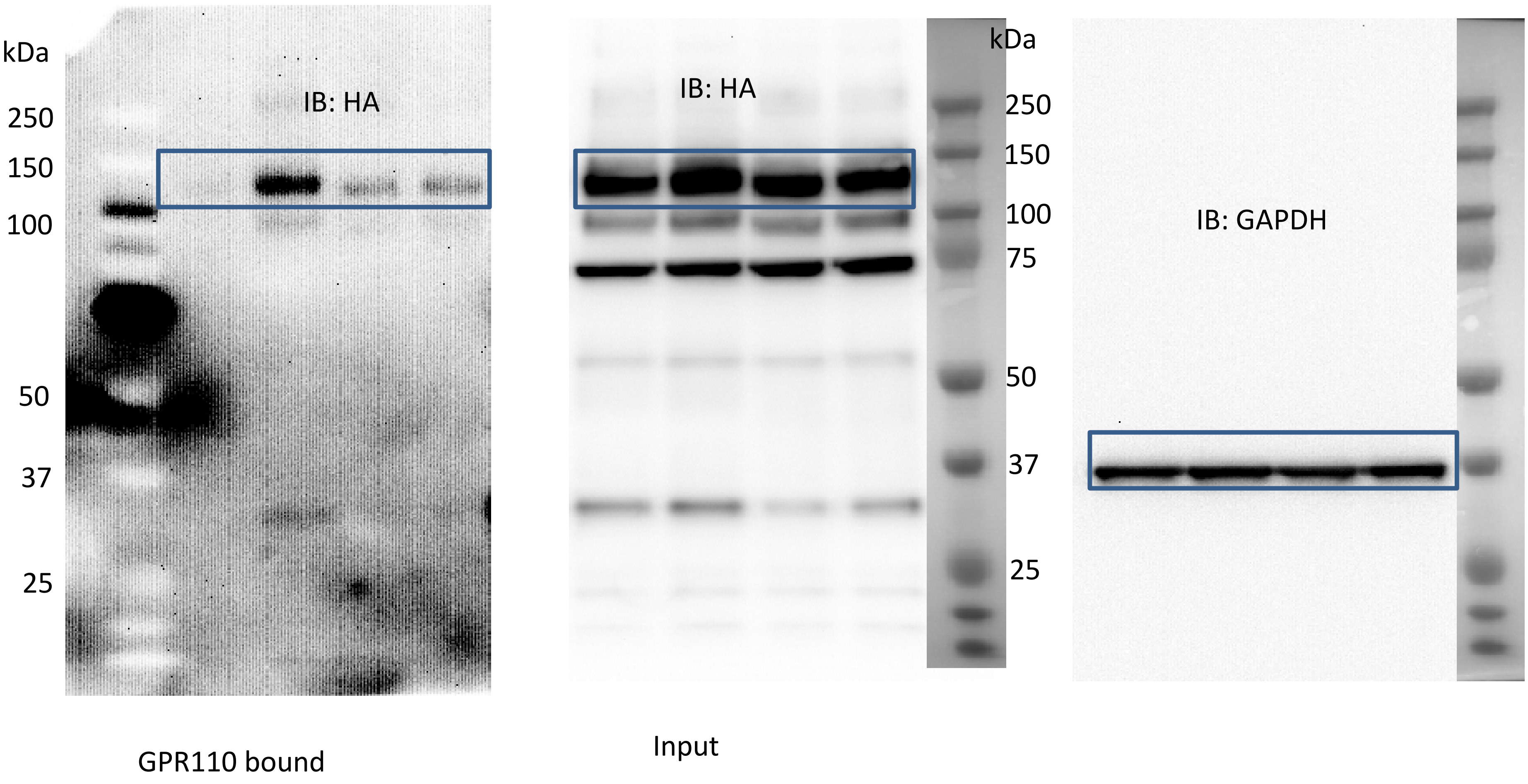
kDa
250
150
100
50
37
25
IB: HA
GPR110 bound
IB: HA
Input
kDa
250
150
100
75
50
37
25
IB: GAPDH

For Fig. 4c

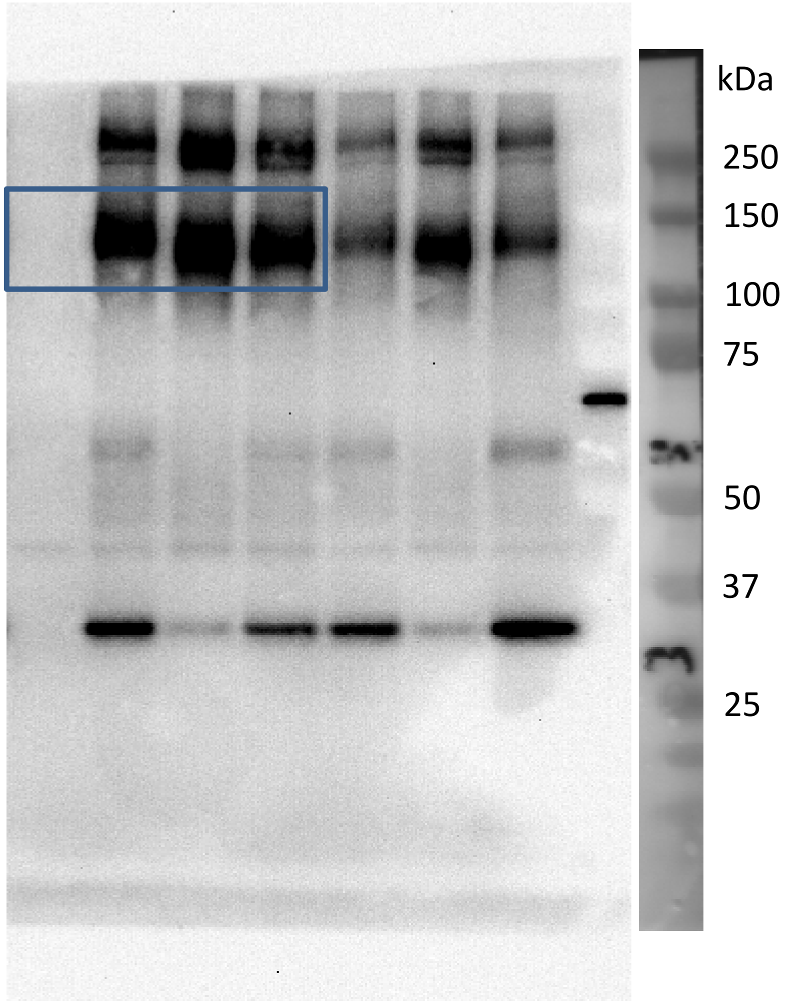

GPR110 at cell surface

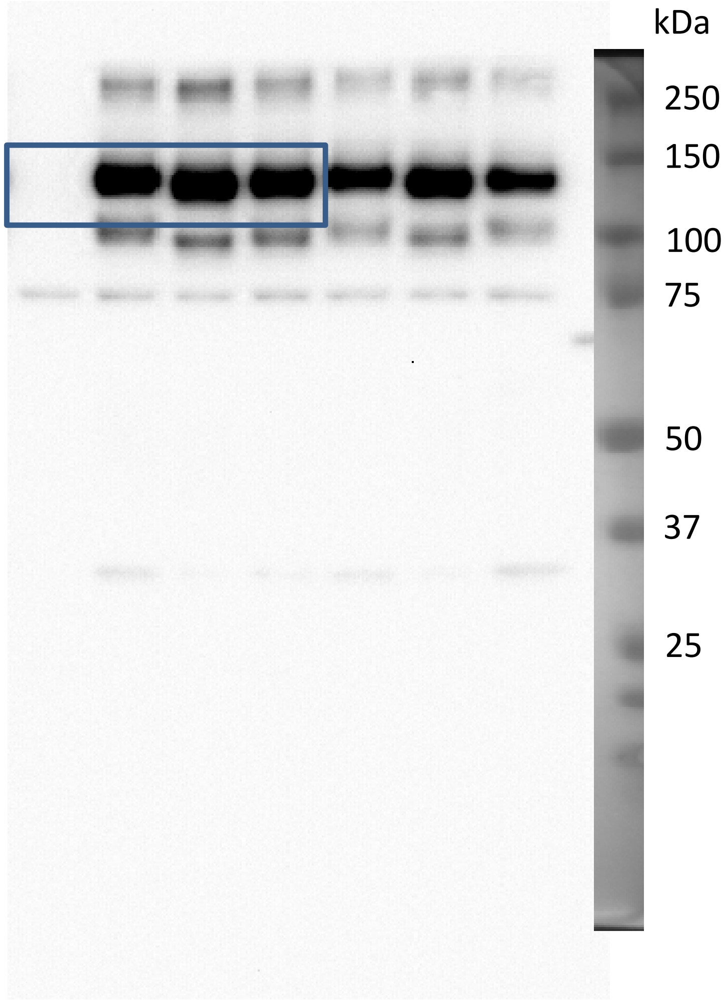

GPR110 input

For Fig. 4c

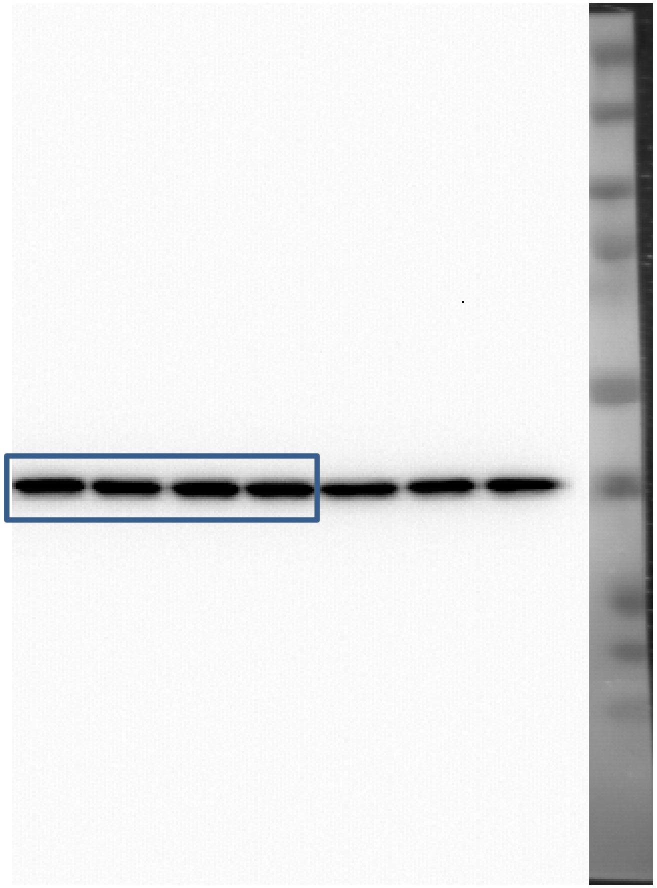

IB: GAPDH input

For Fig. 4d

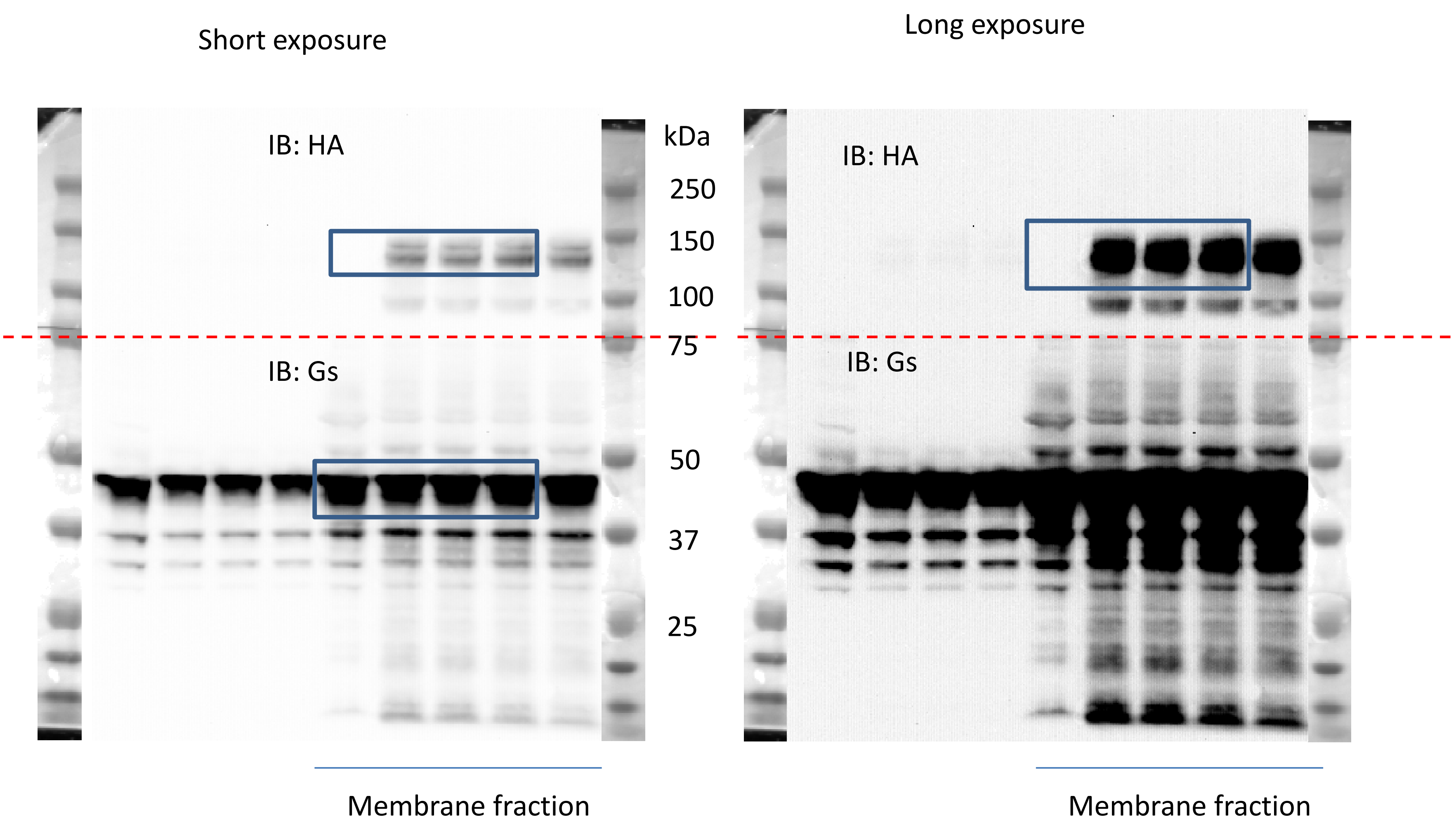
Short exposure
Long exposure
IB: HA
IB: Gs
kDa
250
150
100
75
50
37
25
IB: HA
IB: Gs
Membrane fraction
Membrane fraction

For Fig. 4e

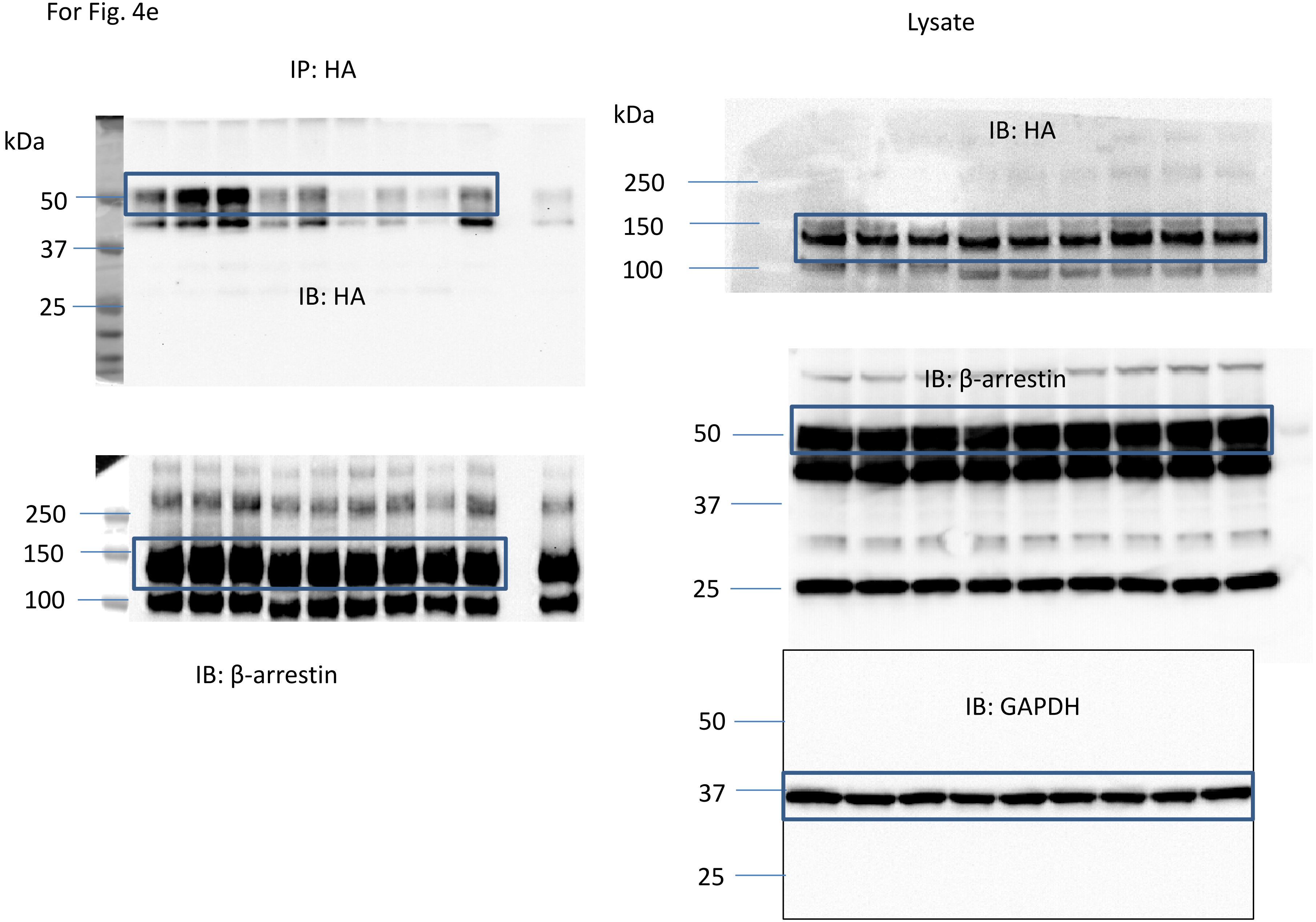
IP: HA
kDa
50
37
25
IB: HA
250
150
100
IB: β-arrestin
Lysate
kDa
250
150
100
IB: HA
IB: β-arrestin
50
37
25
IB: GAPDH
50
37
25

For Supplementary Fig.11a

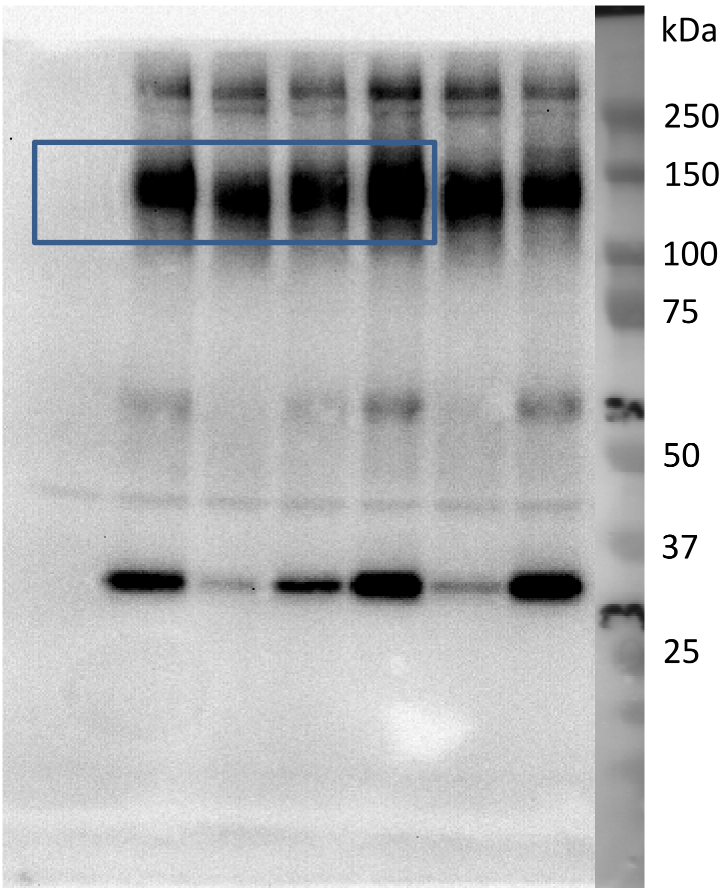

Membrane-expressed GPR110

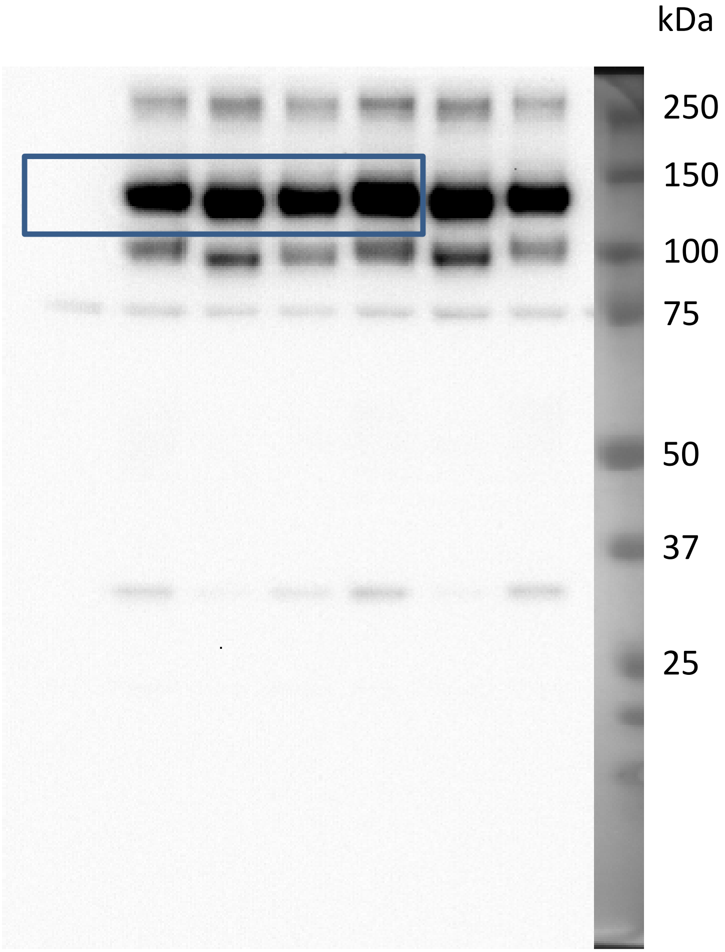

GPR110 input

For Supplementary Fig. 11a

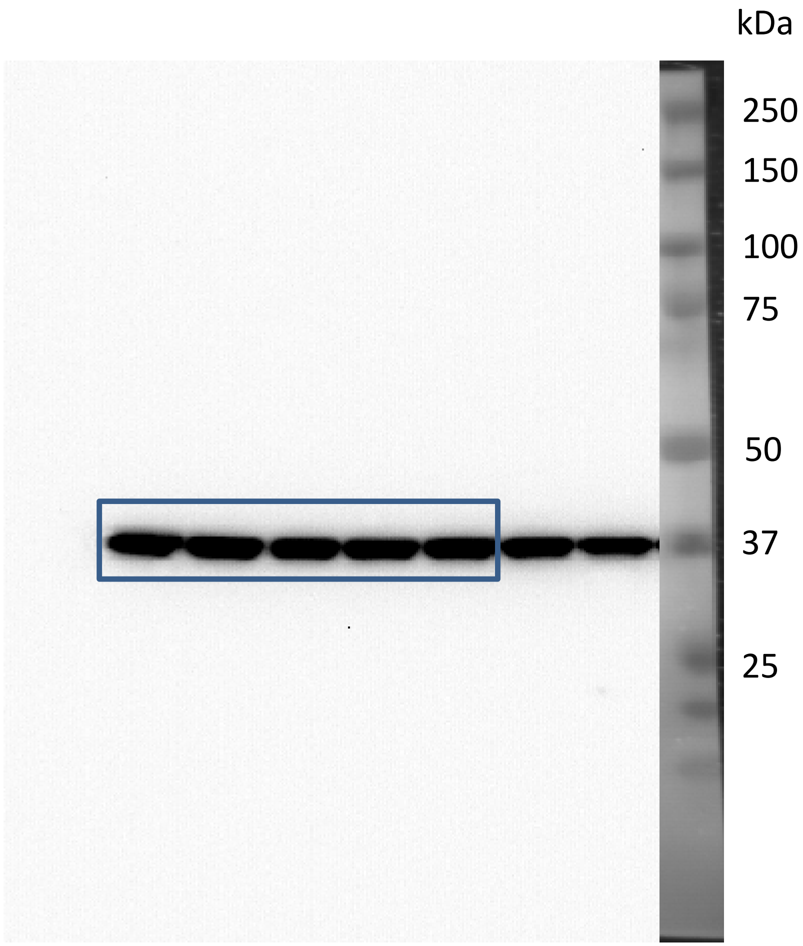

IB: GAPDH input

For Supplementary Fig. 11b

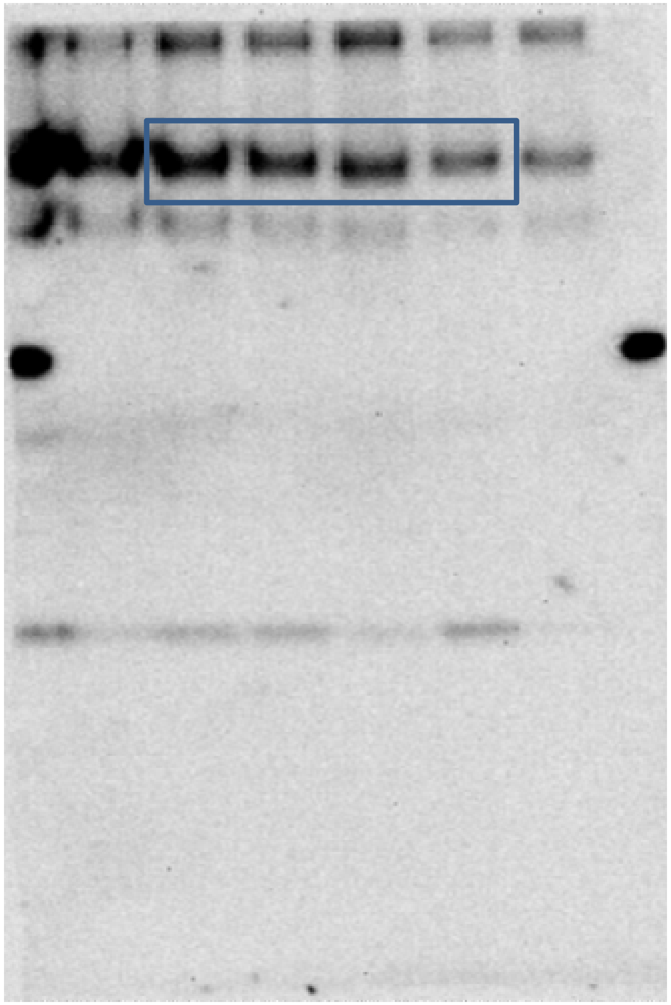

GPR110 bound to biotinylated synaptamide (G1)

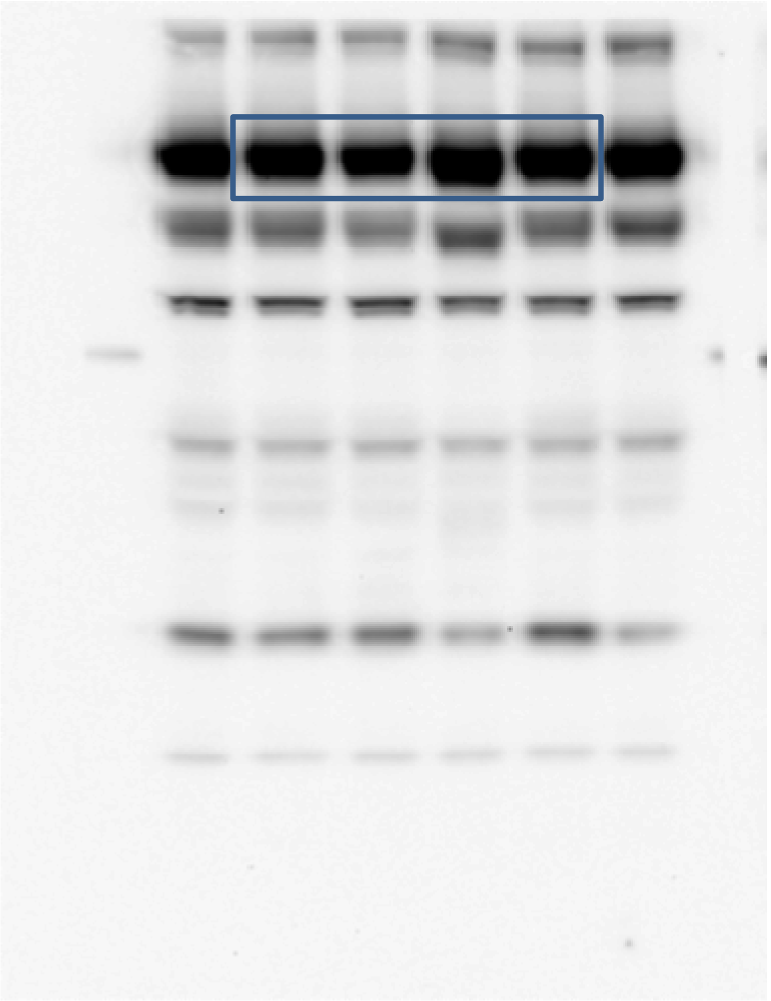

Input

For Supplementary Fig. 13

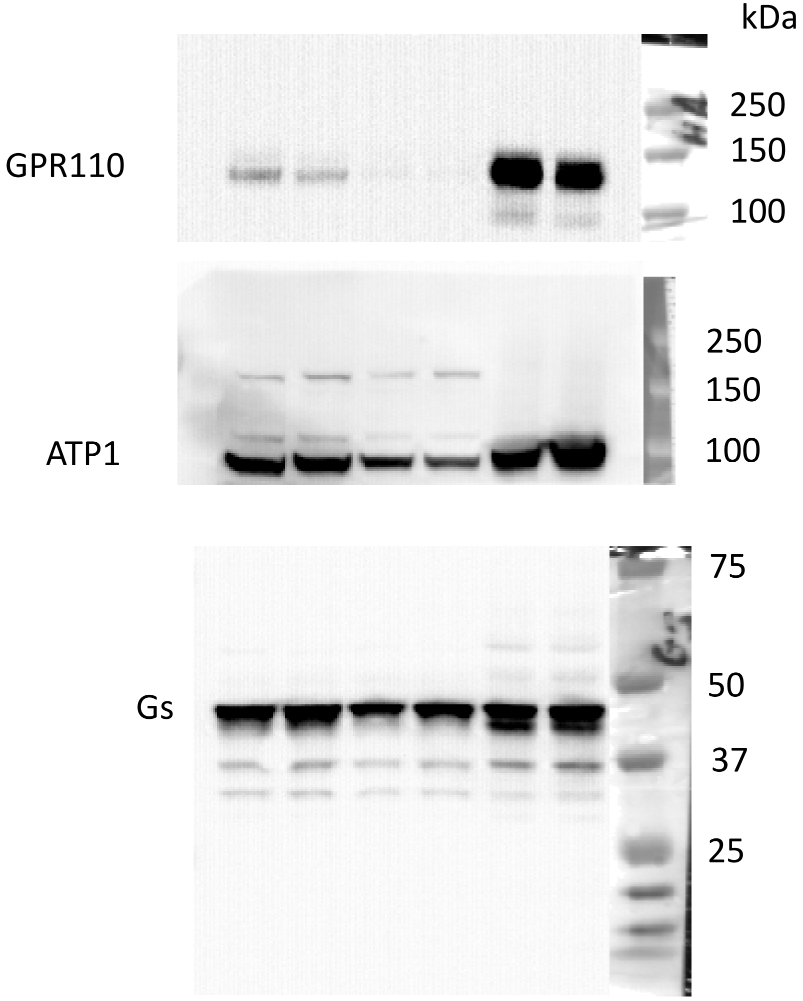

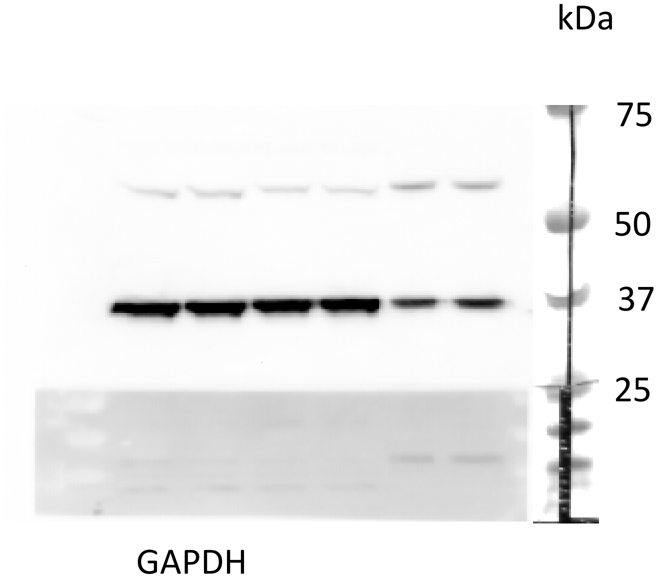

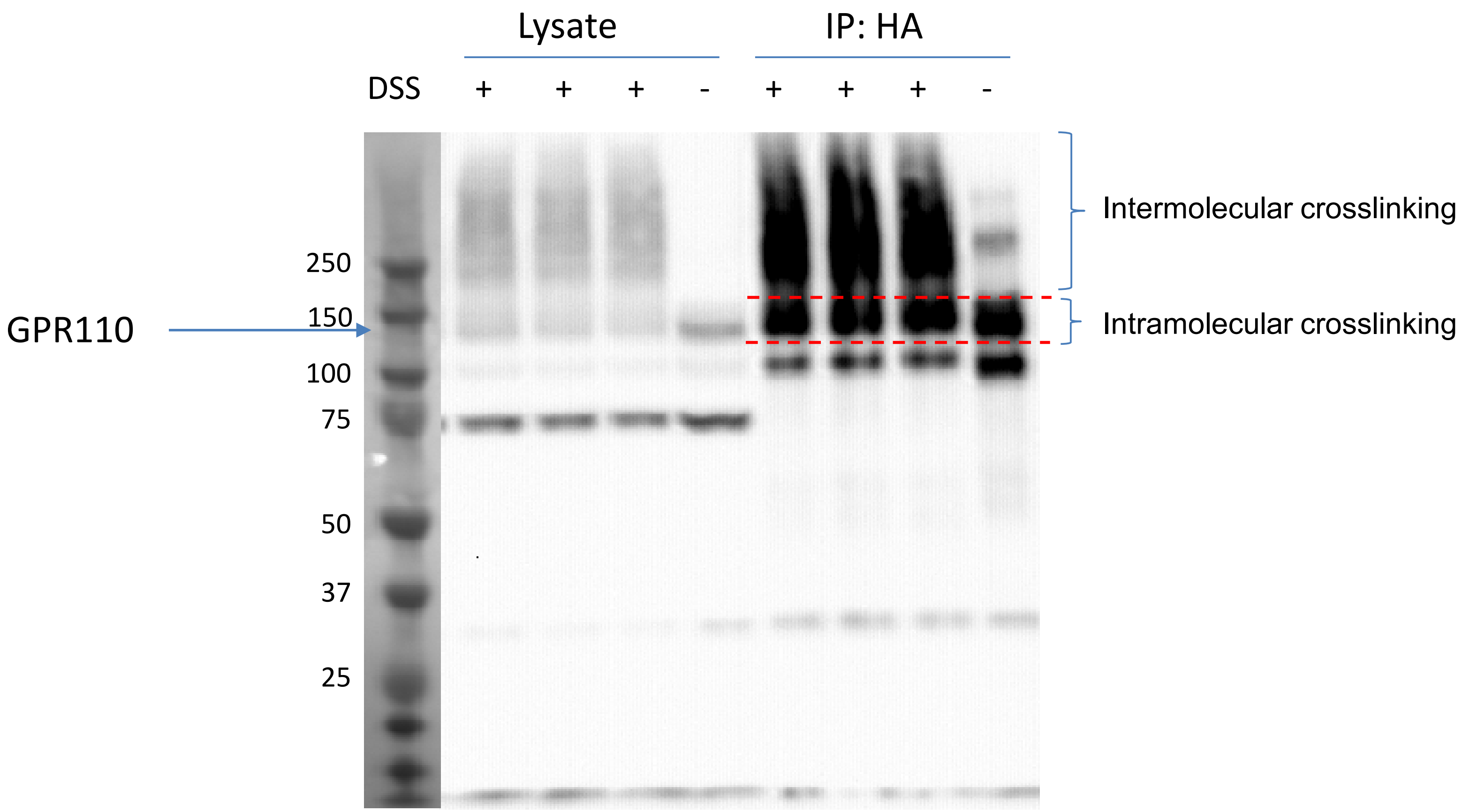

Supplementary Figure 2. Western blot analysis of GPR110-HA with or without in-cell cross-linking by DSS. A major band at ~130 kD is detected for non-cross-linked control, while extensive bands are observed for DSS-modified samples before or after immunopurification. The monomeric band at ~130 kD (marked with red lines) was subjected to mass spectrometric analysis for intramolecular cross-linking of GPR110.

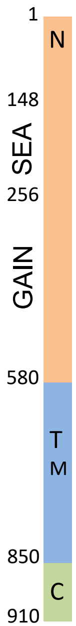

1 MKVGVLWLIS FFTFTDGHGG FLGKNDGIKT KKELIVNKKK HLGPVEEYQL
51 LLQVTYRDSK EKRDLRNFLK LLKPPLLWSH GLIRIIRAKA TTDCNSLNGV
101 LQCTCEDSYT WFPPSCLDPQ NCYLHTAGAL PSCECHLNNL SQSVNFCERT
151 KIWGTFKINE RFTNDLLNSS SAIYSKYANG IEIQLKKAYE RIQGFESVQV
201 TQFRNGSIVA GYEVVGSSSA SELLSAIEHV AEKAKTALHK LFPLEDGSFR
251 VFGKAQCNDI VFGFGSKDDE YTLPCSSGYR GNITAKCESS GWQVIRETCV
301 LSLLEELNKN FSMIVGNATE AAVSSFVQNL SVIIRQNPST TVGNLASVVS
351 ILSNISSLSL ASHFRVSNST MEDVISIADN ILNSASVTNW TVLLREEKYA
401 SSRLLETLEN ISTLVPPTAL PLNFSRKFID WKGIPVNKSQ LKRGYSYQIK
451 MCPQNTSIPI RGRVLIGSDQ FQRSLPETII SMASLTGLNI LPVSKNGNAQ
501 VNGPVISTVI QNYSINEVFL FFSKIESNLS QPHCVFWDFS HLQWNDAGCH
551 LVNETQDIVT CQCTHLTSFS ILMSPFVPST IFPVVKWITY VGLGISIGSL
601 ILCLIIEALF WKQIKKSQTS HTRRICMVNI ALSLLIADVW FIVGATVDTT
651 VNPSGVCTAA VFFTHFFYLS LFFWMLMLGI LLAYRIILVF HHMAQHLMMA
701 VGFCLGYGCP LIISVITIAV TQPSNTYKRK DVCWLNWSNG SKPLLAFVVP
751 ALAIVAVNFV VVLLVLTKLW RPTVGERLSR DDKATIIRVG KSLLILTPLL
801 GLTWGFGIGT IVDSQNLAWH VIFALLNAFQ GFFILCFGIL LDSKLRQLLF
851 NKLSALSSWK QTEKQNSSDL SAKPKFSKPF NPLQNKGHYA FSHTGDSSDN
901 IMLTQFVSNE

Supplementary Figure 3. Mass spectrometric detection of GPR110. After cross-linking by DSS, GPR110-HA transfected HEK cells were lysed and subjected to immunoprecipitation with anti-HA antibody. Proteins were subjected to SDS-PAGE, reduction/alkylation, tryptic digestion and LC/MS/MS analysis. GPR110 was identified for the monomeric band (100-150 kDa). Peptides identified are highlighted in red. SEA, sea urchin sperm protein, enterokinase and agrin domain; GAIN, GPCR-autoproteolysis-inducing domain; TM, 7-transmembrane domain; N, *N*-terminus, C, *C*-terminus.

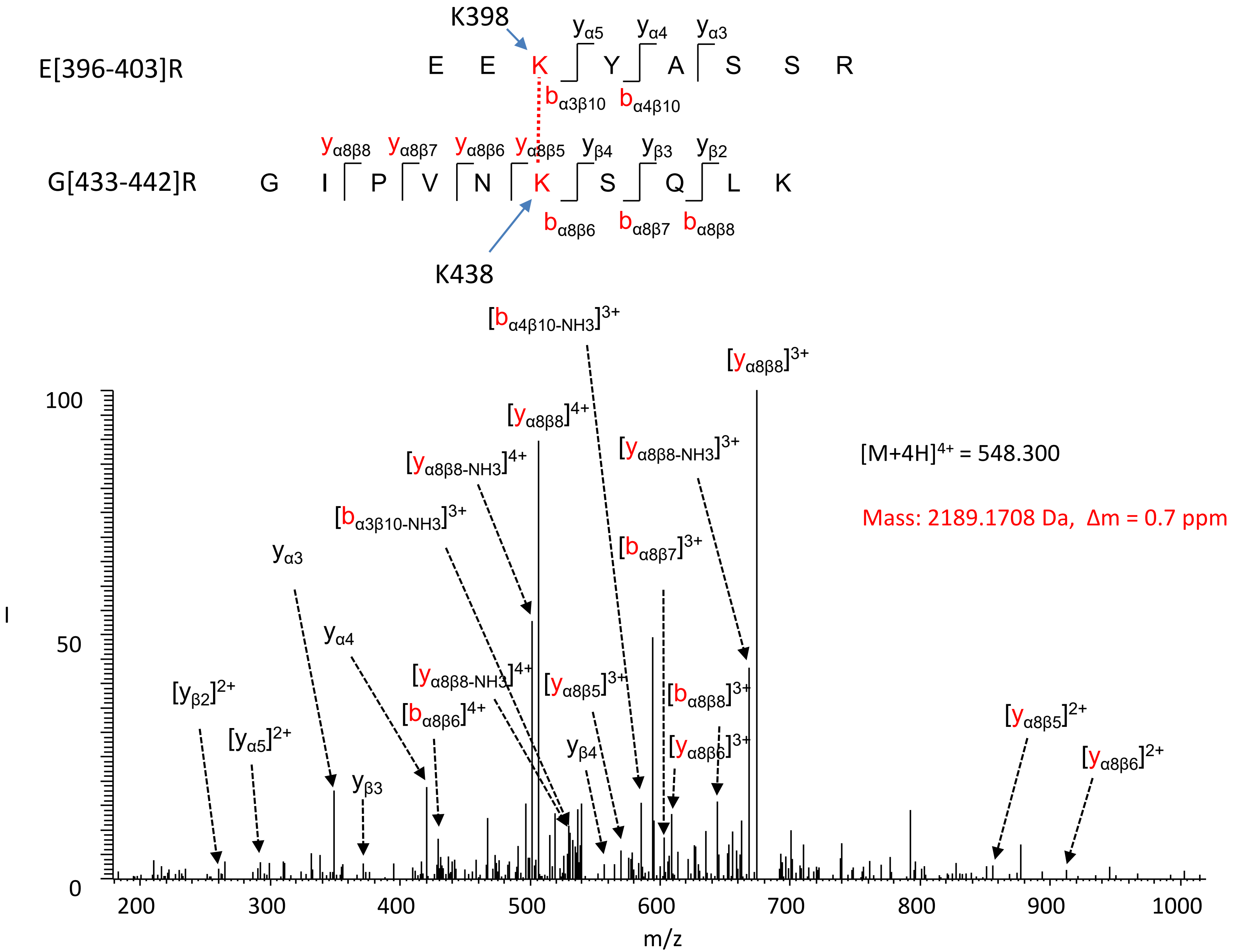

Supplementary Figure 4. MS/MS analysis of a through-space cross-linked peptide in GPR110 GAIN domain. The MS/MS data revealed that the peptide with mass of 2189.1708 Da reconstructed from quadruply charged ion of m/z 548.300, originated from E[396-403]R and G[433-442]R with K398 linked to K438 via DSS. Fragment ions involving both peptide segments (designated as α and β respectively) are marked in red.

Supplementary Figure 5. MS/MS identification of cross-linked peptides by xQuest. The parameters used in the xQuest include cross-link mass shift of 138.06808 Dalton, MS1 tolerance of 10 ppm, and MS2 tolerance of 0.3 m/z. a, K442 linked to K427. b, K398 linked to K427. c, K438 linked to K427 d, K187 linked to K240. e, K438 linked to K442. f, K38 linked to K39. g, K38 linked to K29. h, K427 linked to K432. i, K151 linked to K157. j, K186 linked to K187. k, K151 linked to K157. l, K39 linked to K40. m, K432 linked to K438. n, K442 linked to K151. o, K31 linked to K32. p, K852 linked to K860. q, K875 linked to K878. r, K151 linked to K254. s, K240 linked to K254. t, K398 linked to K442. u, K860 linked to K864. v, K864 linked to K873. w, K151 linked to K187.

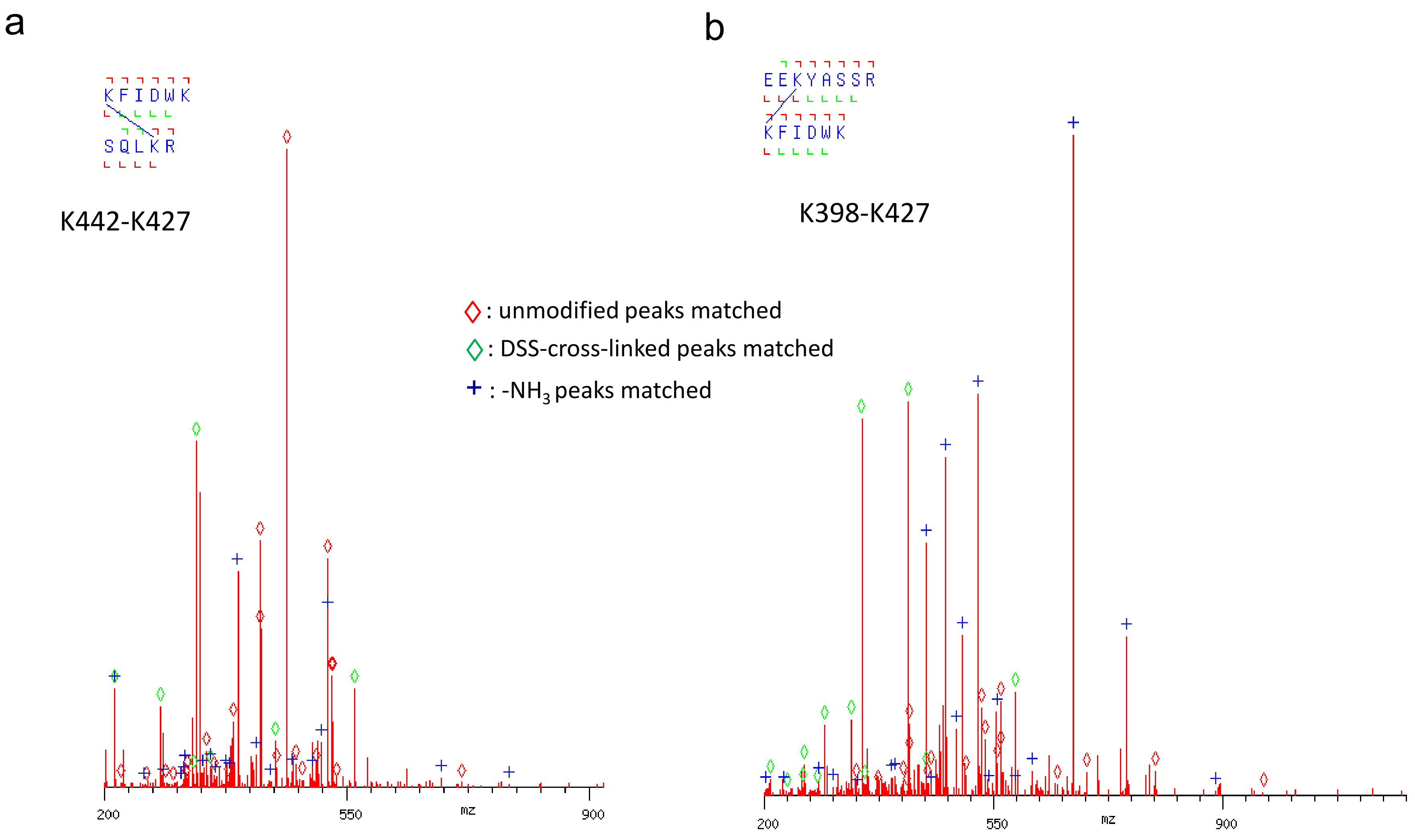

e K438-K442

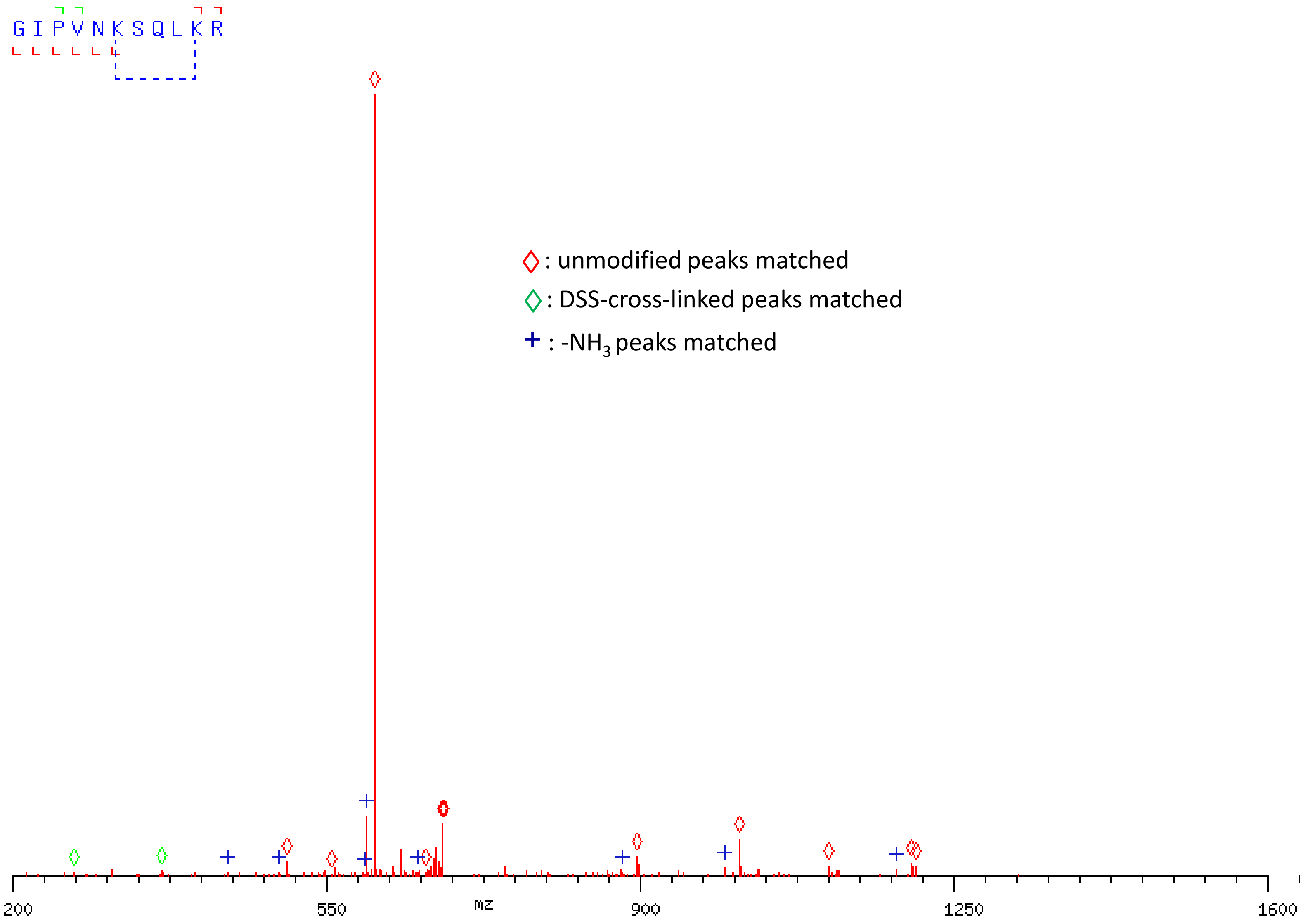

G I P V N K S Q L K R
: unmodified peaks matched
: DSS-cross-linked peaks matched
: -NH3 peaks matched
200
550
mz
900
1250
1600

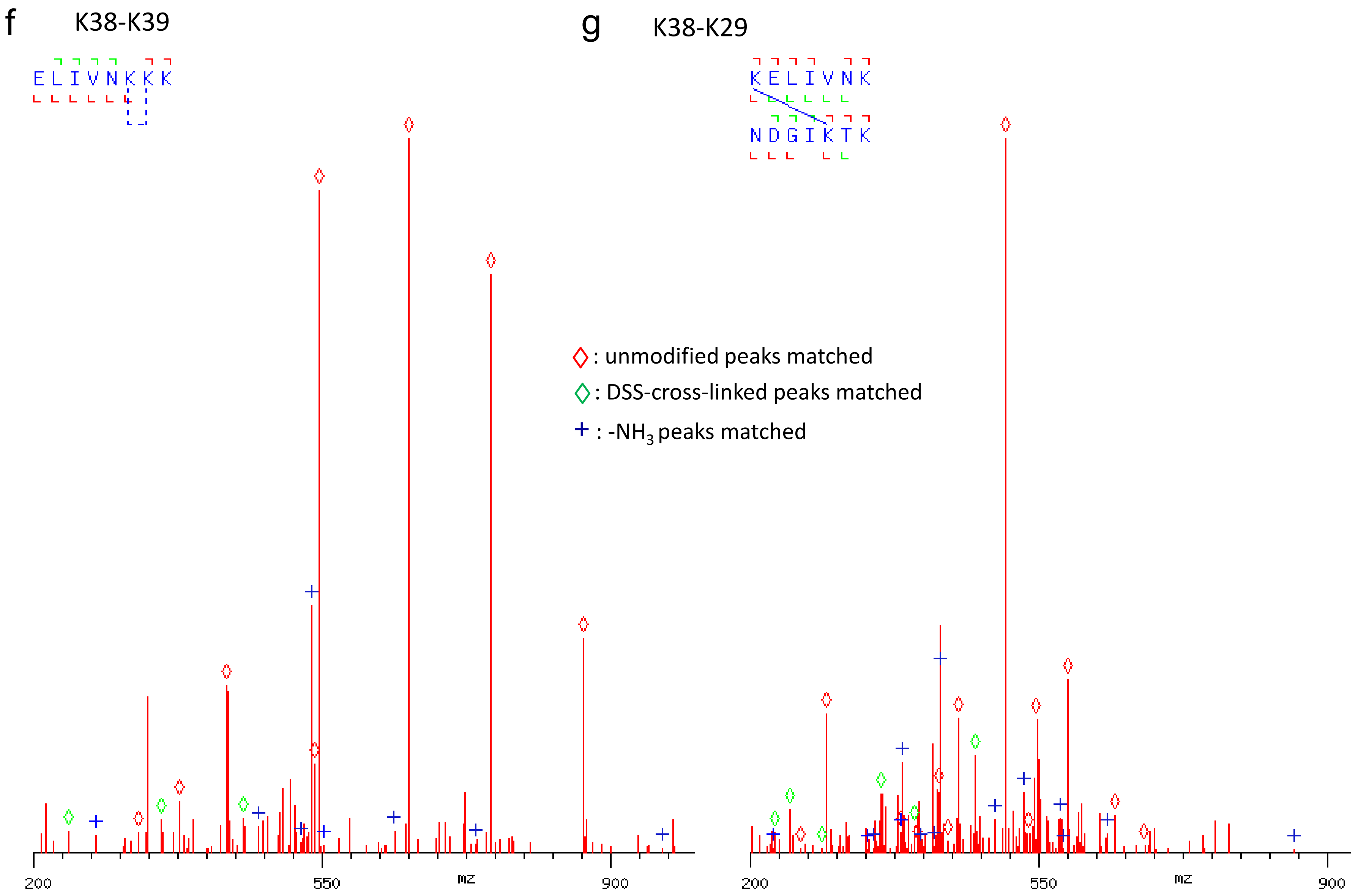

f
K38-K39
E L I V N K K K
g
K38-K29
K E L I V N K
N D G I K T K
: unmodified peaks matched
: DSS-cross-linked peaks matched
: -NH3 peaks matched
200
550
mz
900

h K427-K432

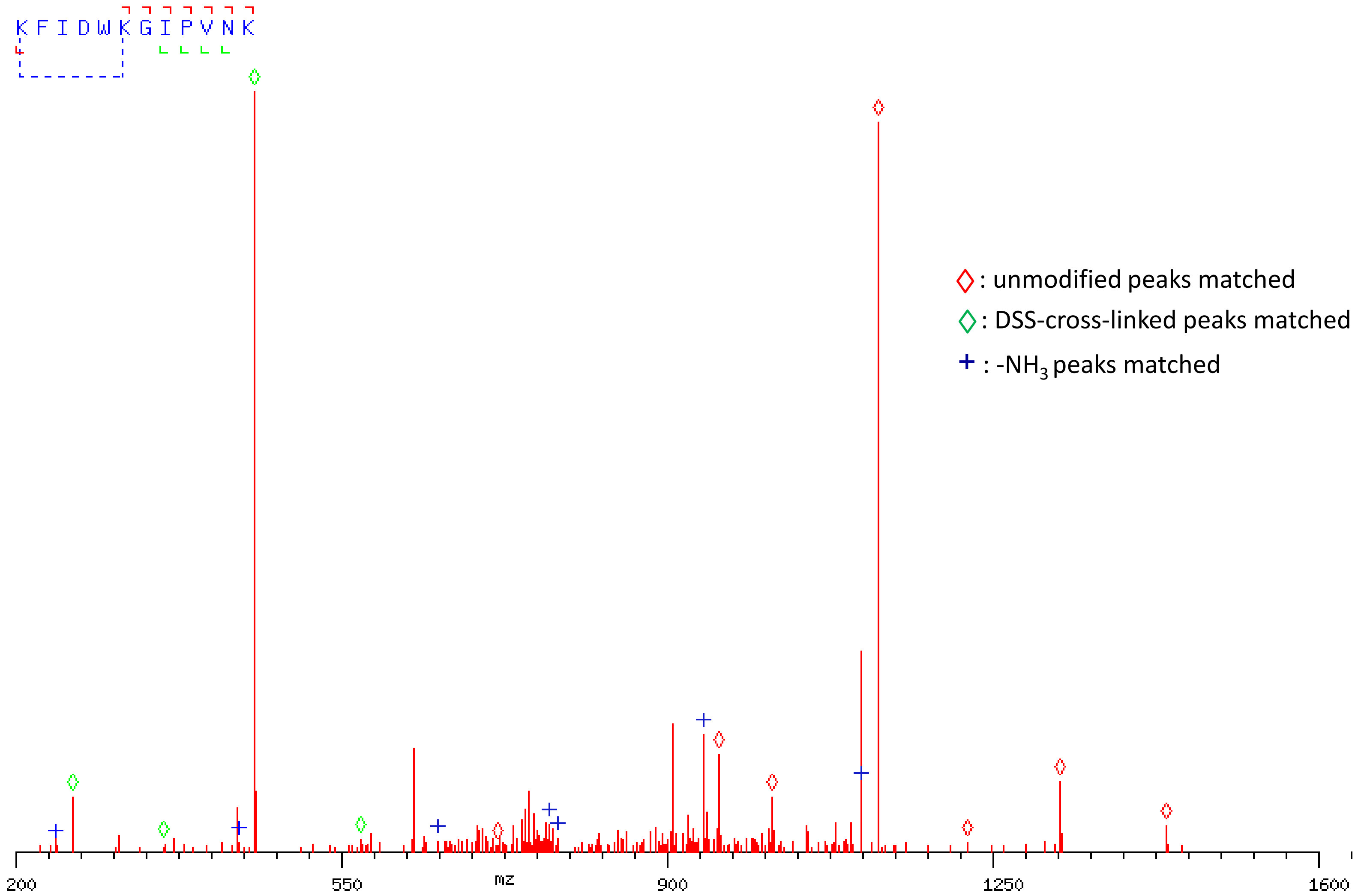
K F I D W K G I P V N K
: unmodified peaks matched
: DSS-cross-linked peaks matched
: -NH_3 peaks matched
200
550
mz
900
1250
1600

c K438-K427

d K187-K240

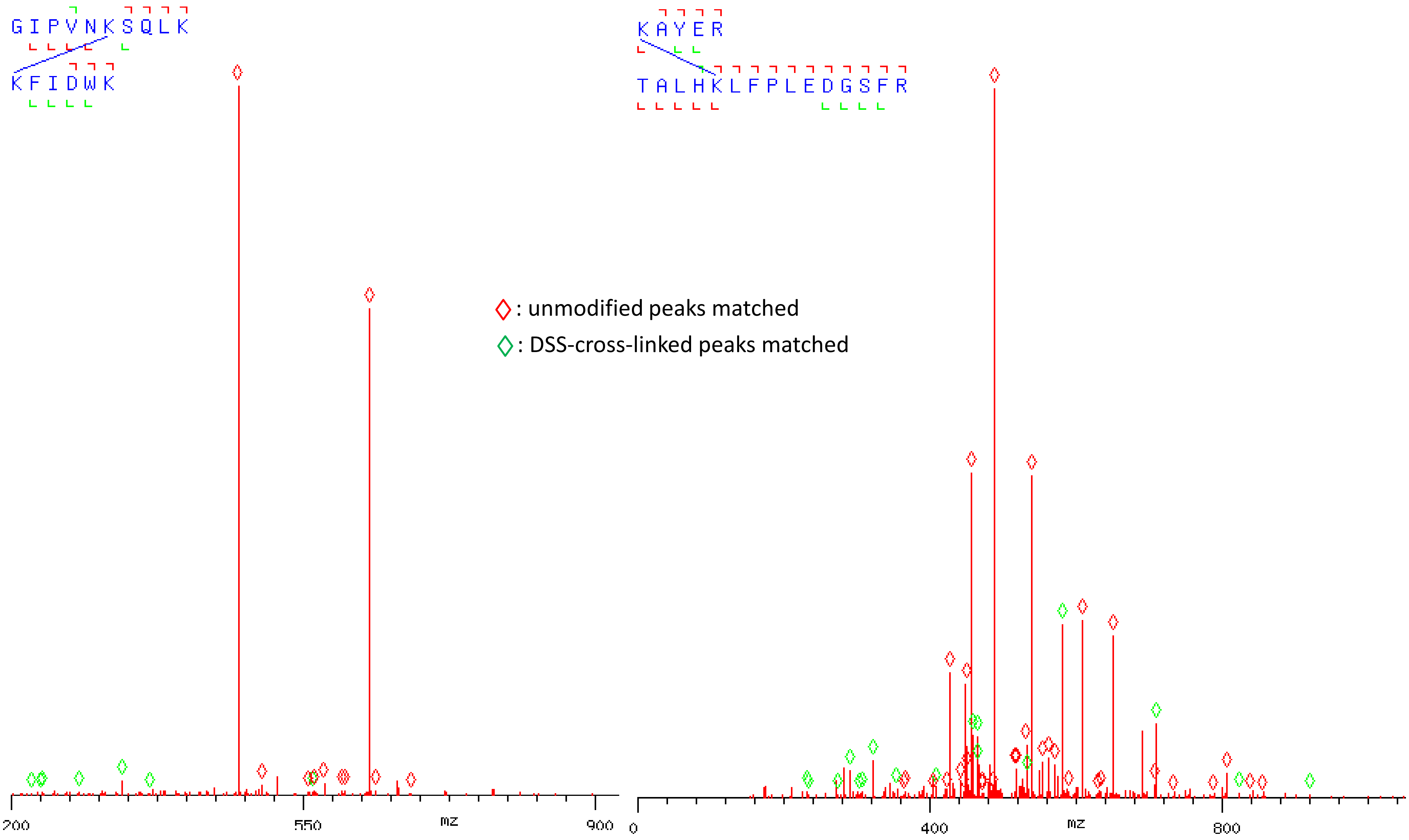

i K235-K240

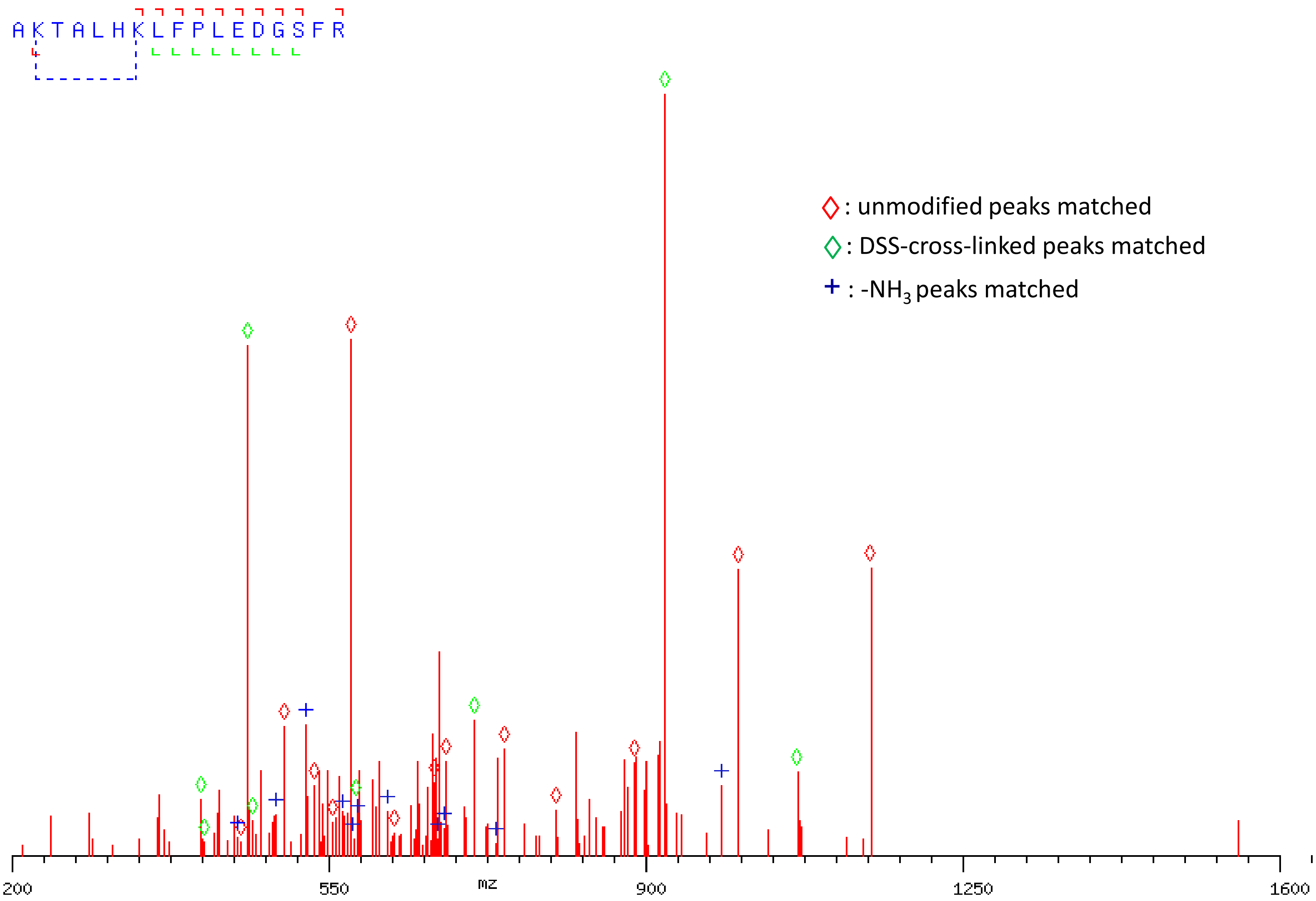

A K T A L H K L F P L E D G S F R
: unmodified peaks matched
: DSS-cross-linked peaks matched
: -NH3 peaks matched
200
550
mz
900
1250
1600

j K186-K187

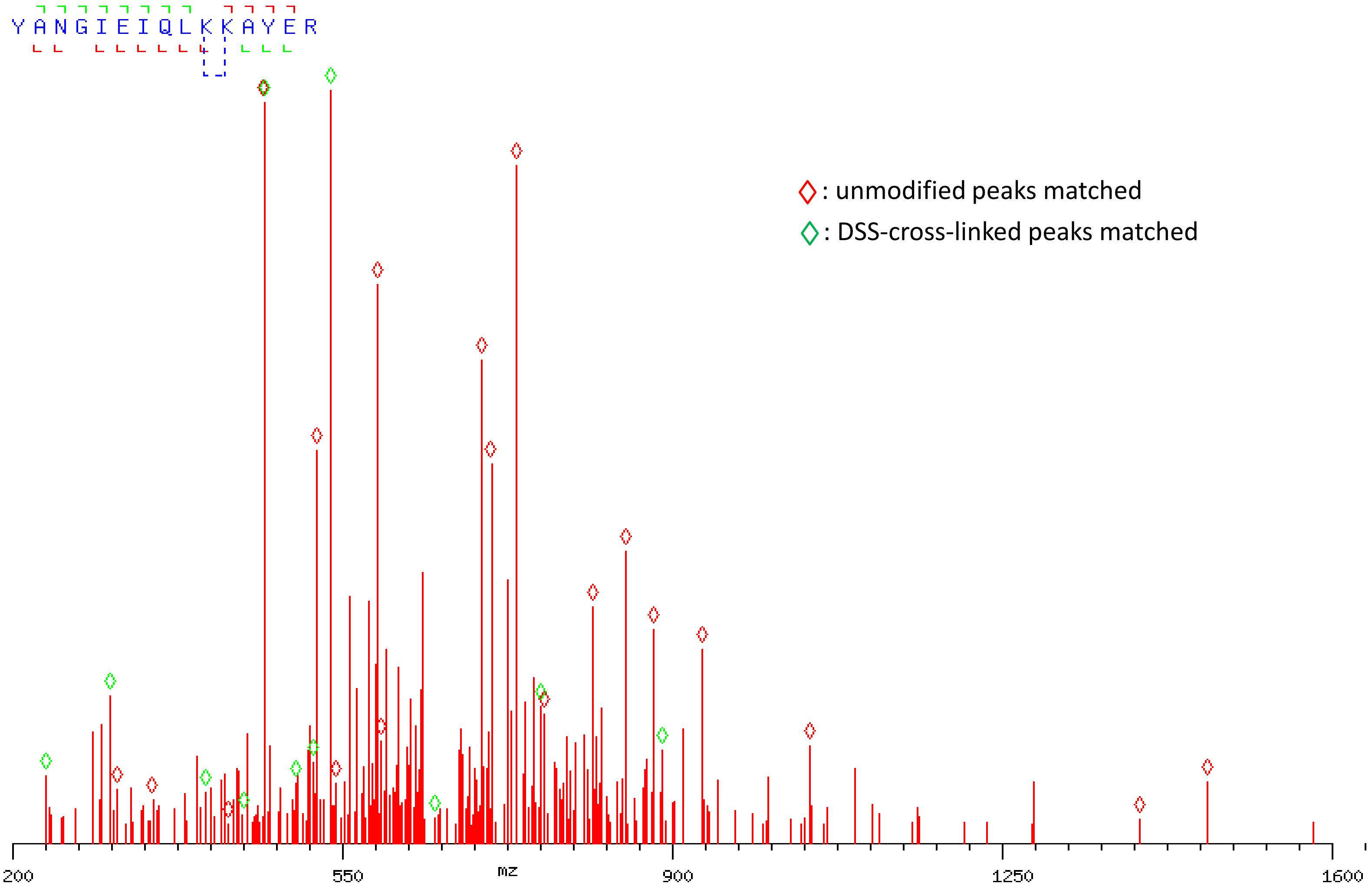

Y A N G I E I Q L K K A Y E R
: unmodified peaks matched
: DSS-cross-linked peaks matched
200
550
mz
900
1250
1600

k K151-K157

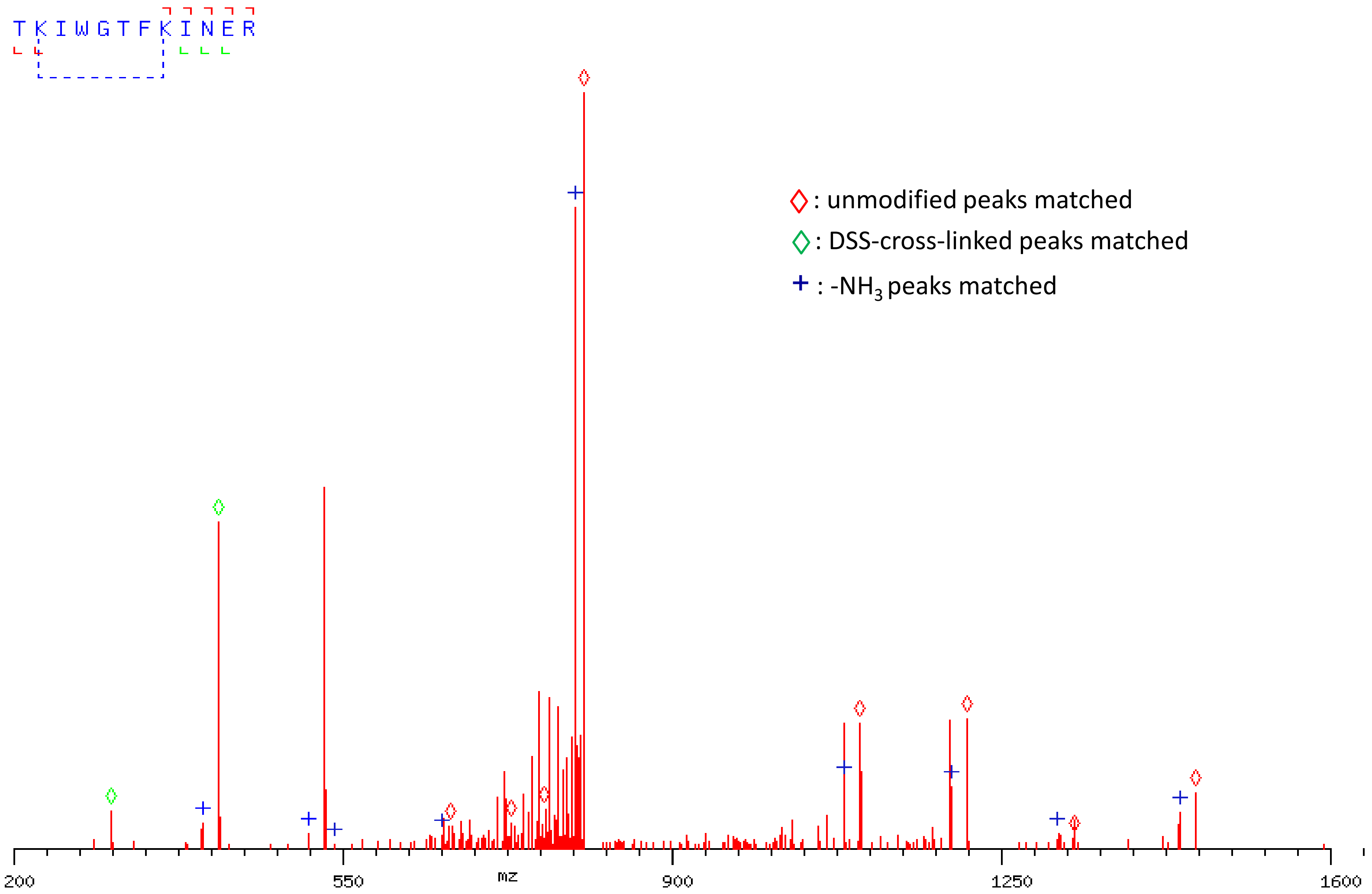

T K I W G T F K I N E R
◊ : unmodified peaks matched
◊ : DSS-cross-linked peaks matched
+ : -NH3 peaks matched
200
550
mz
900
1250
1600

I

K39-K40

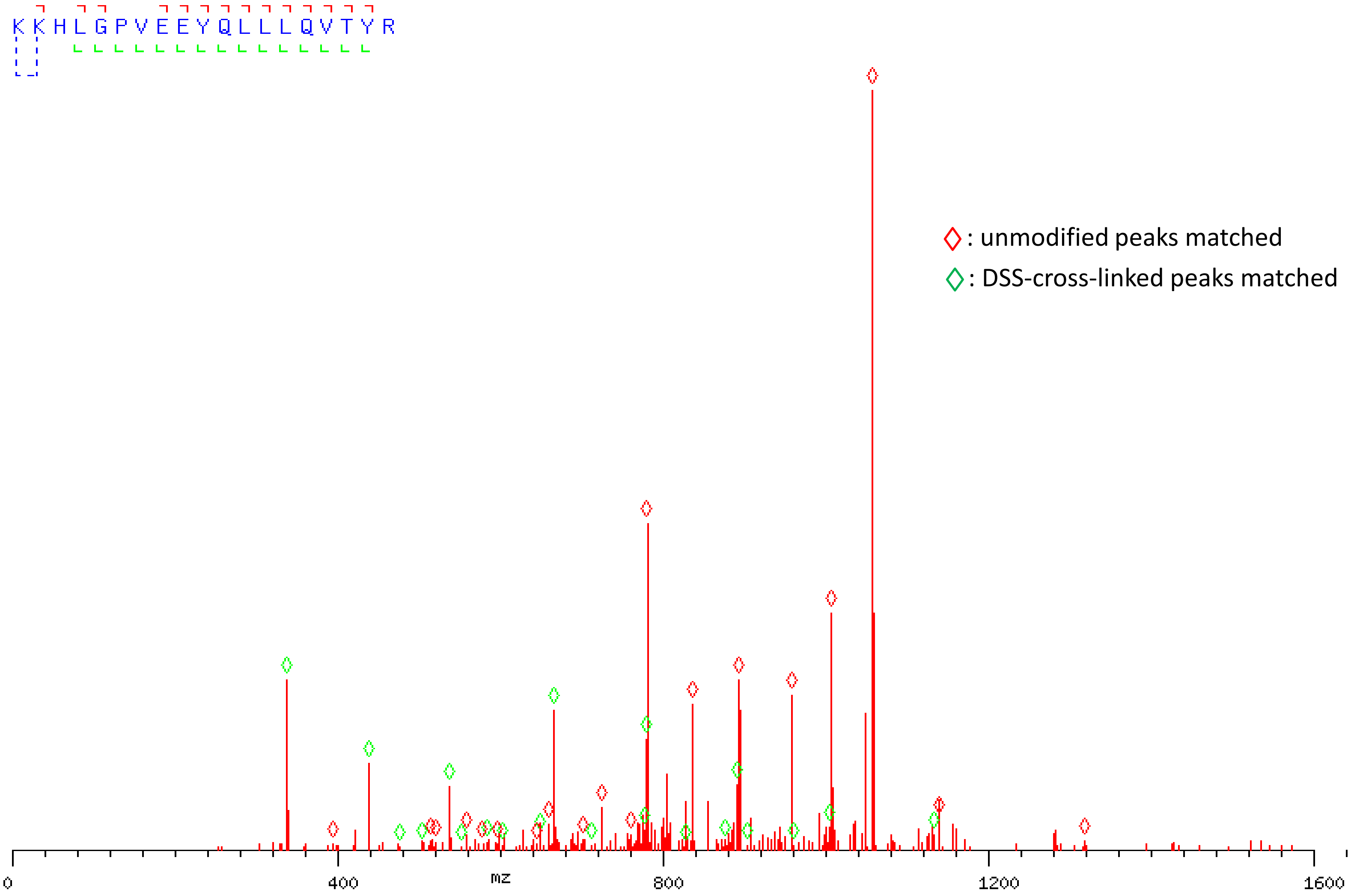

K K H L G P V E E Y Q L L L Q V T Y R
: unmodified peaks matched
: DSS-cross-linked peaks matched
mz
0
400
800
1200
1600

m K432-K438

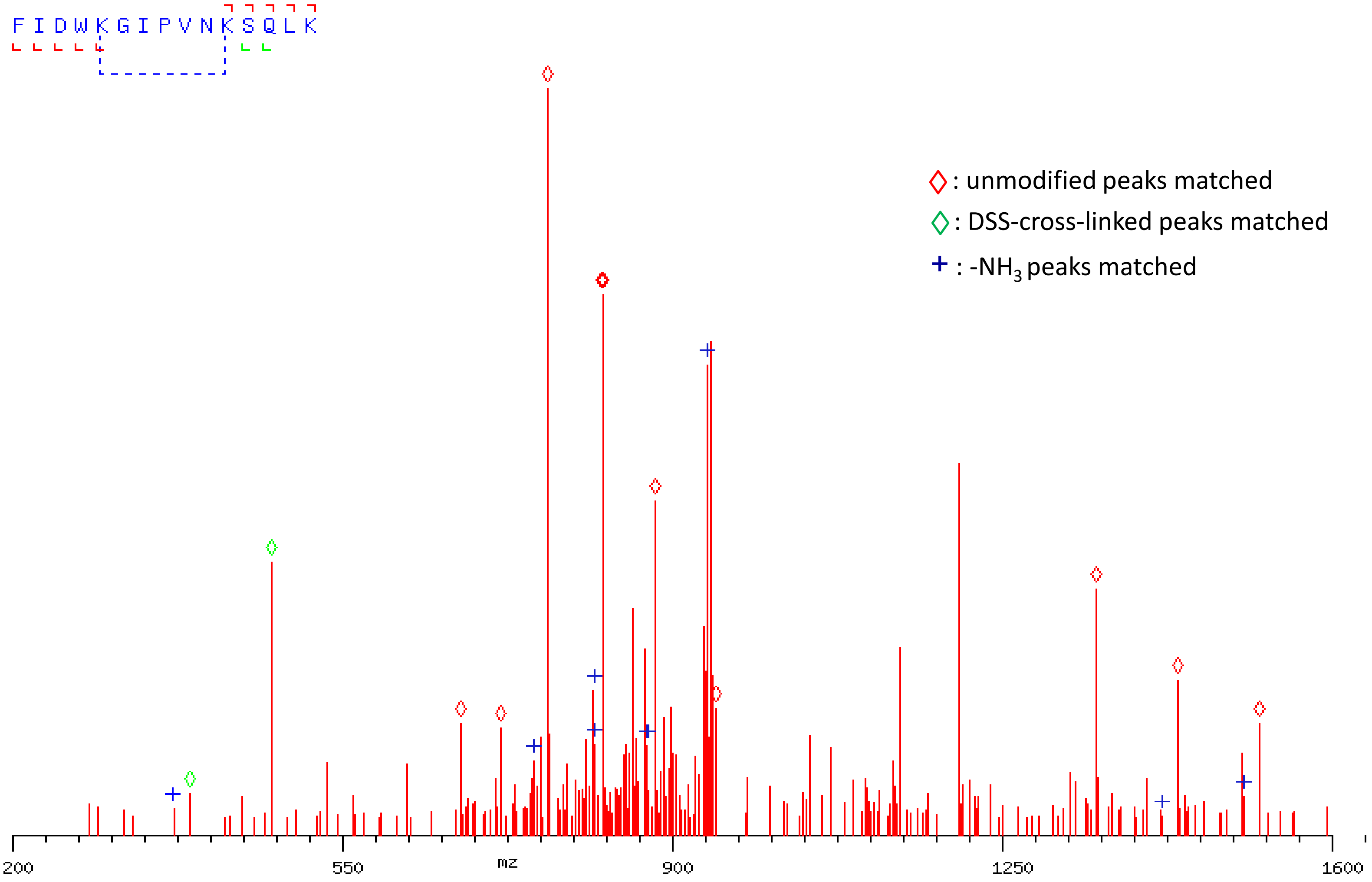
F I D W K G I P V N K S Q L K
: unmodified peaks matched
: DSS-cross-linked peaks matched
: -NH3 peaks matched
200
550
mz
900
1250
1600

n K151-K442

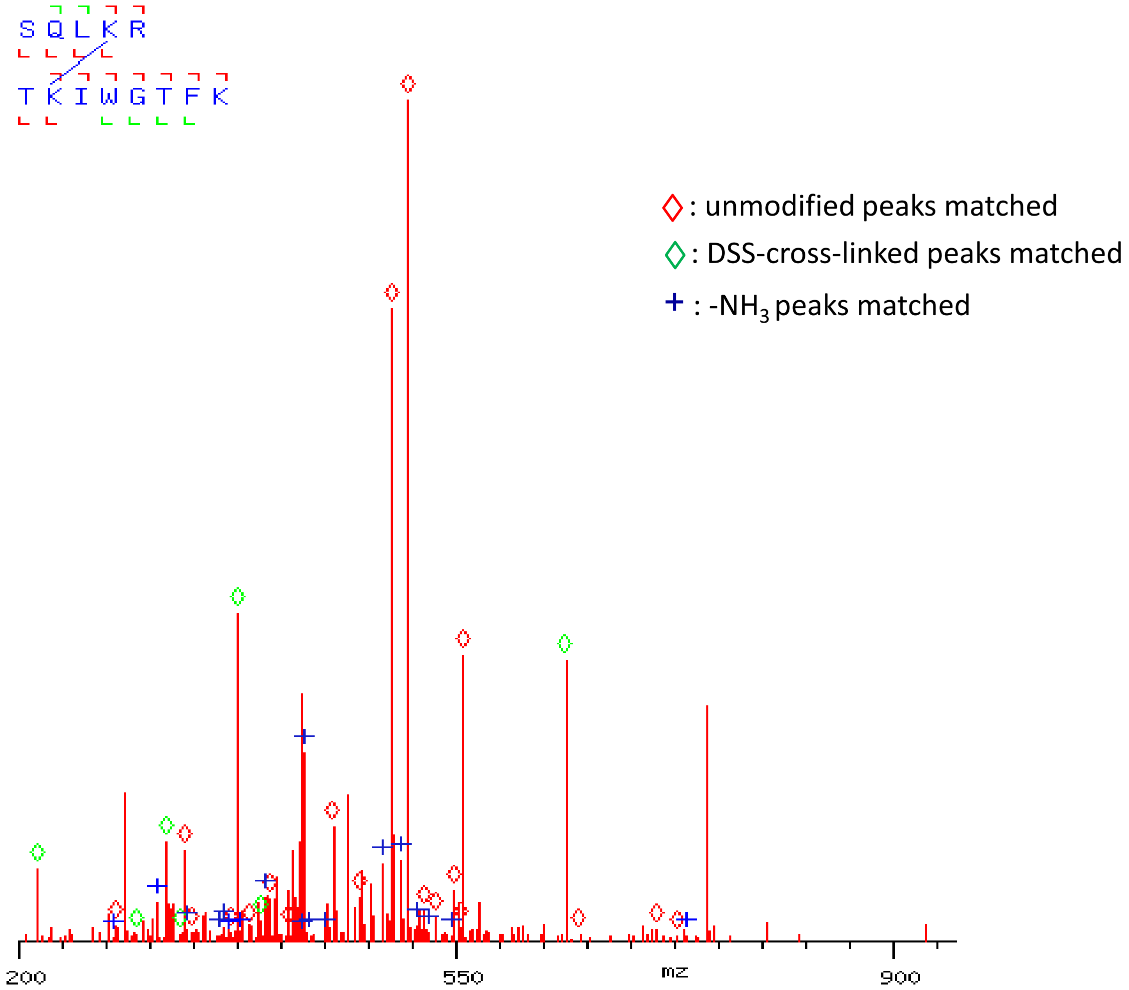

S Q L K R
T K I W G T F K
◇ : unmodified peaks matched
◇ : DSS-cross-linked peaks matched
+ : -NH_3 peaks matched
200
550
mz
900

O K31-K32

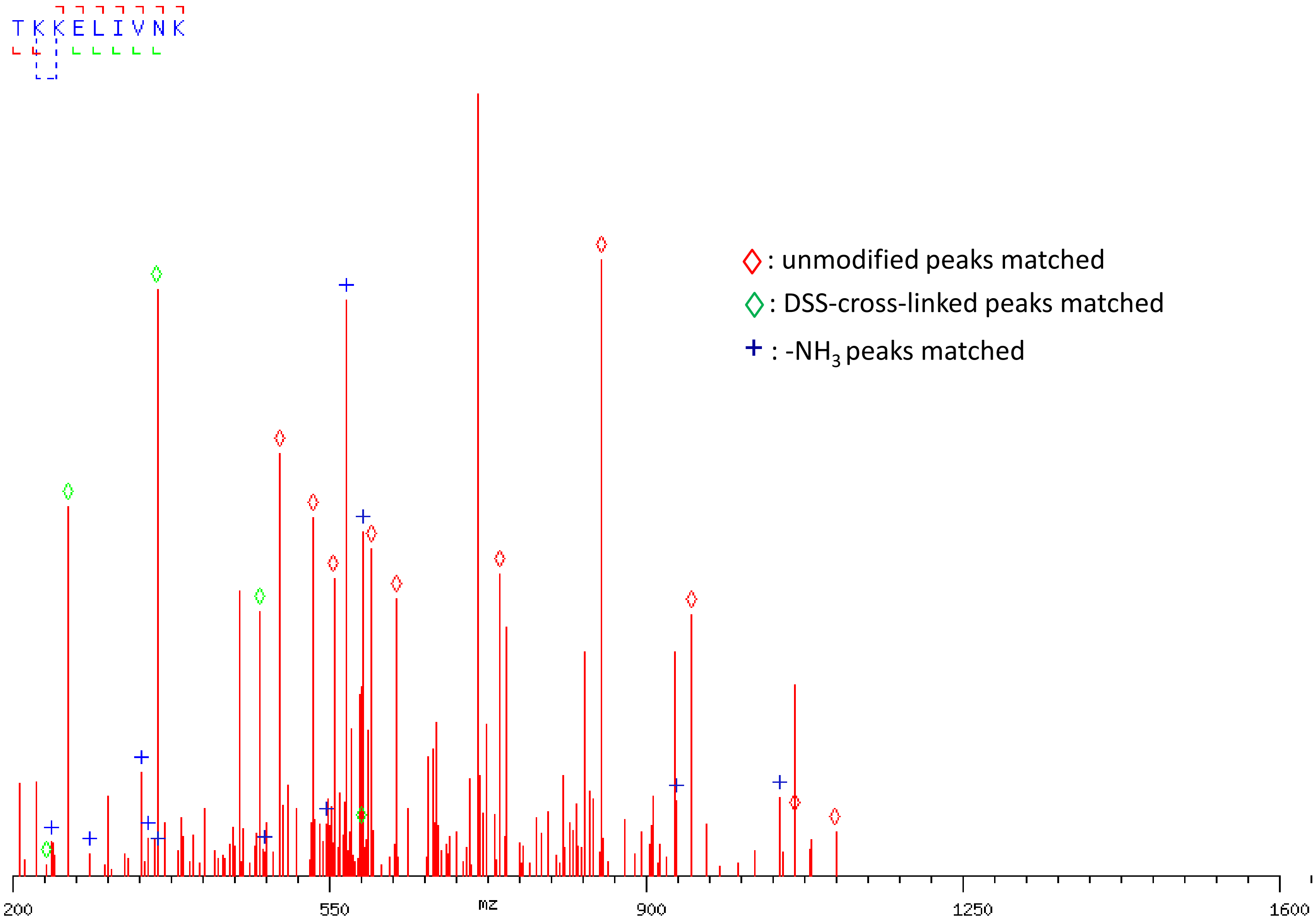

T K K E L I V N K
: unmodified peaks matched
: DSS-cross-linked peaks matched
: -NH3 peaks matched
200
550
mz
900
1250
1600

p K852-K860

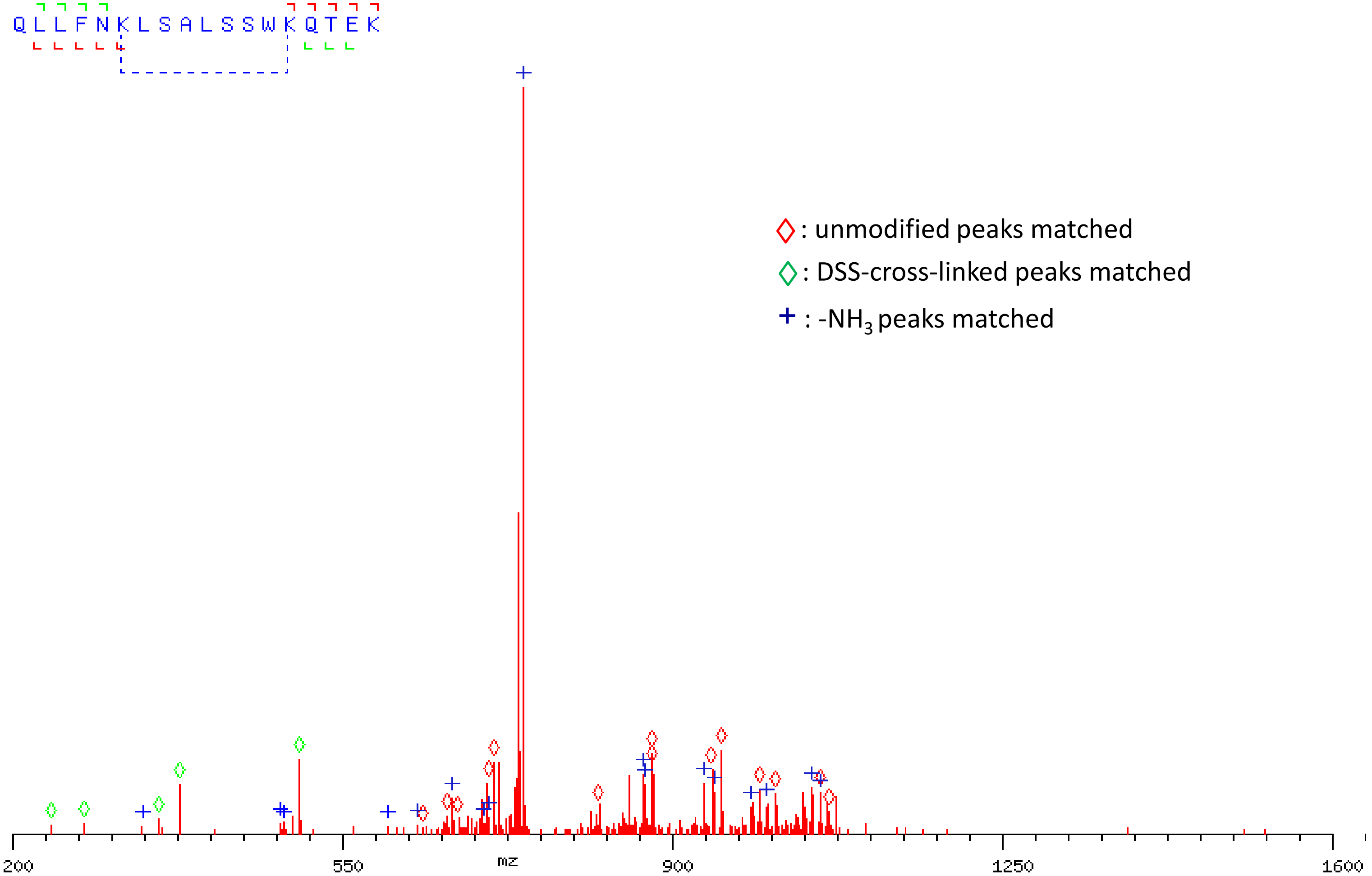
QLLFNKLSALSSWKQTEK
: unmodified peaks matched
: DSS-cross-linked peaks matched
: -NH_3 peaks matched
200
550
mz
900
1250
1600

q K875-K878

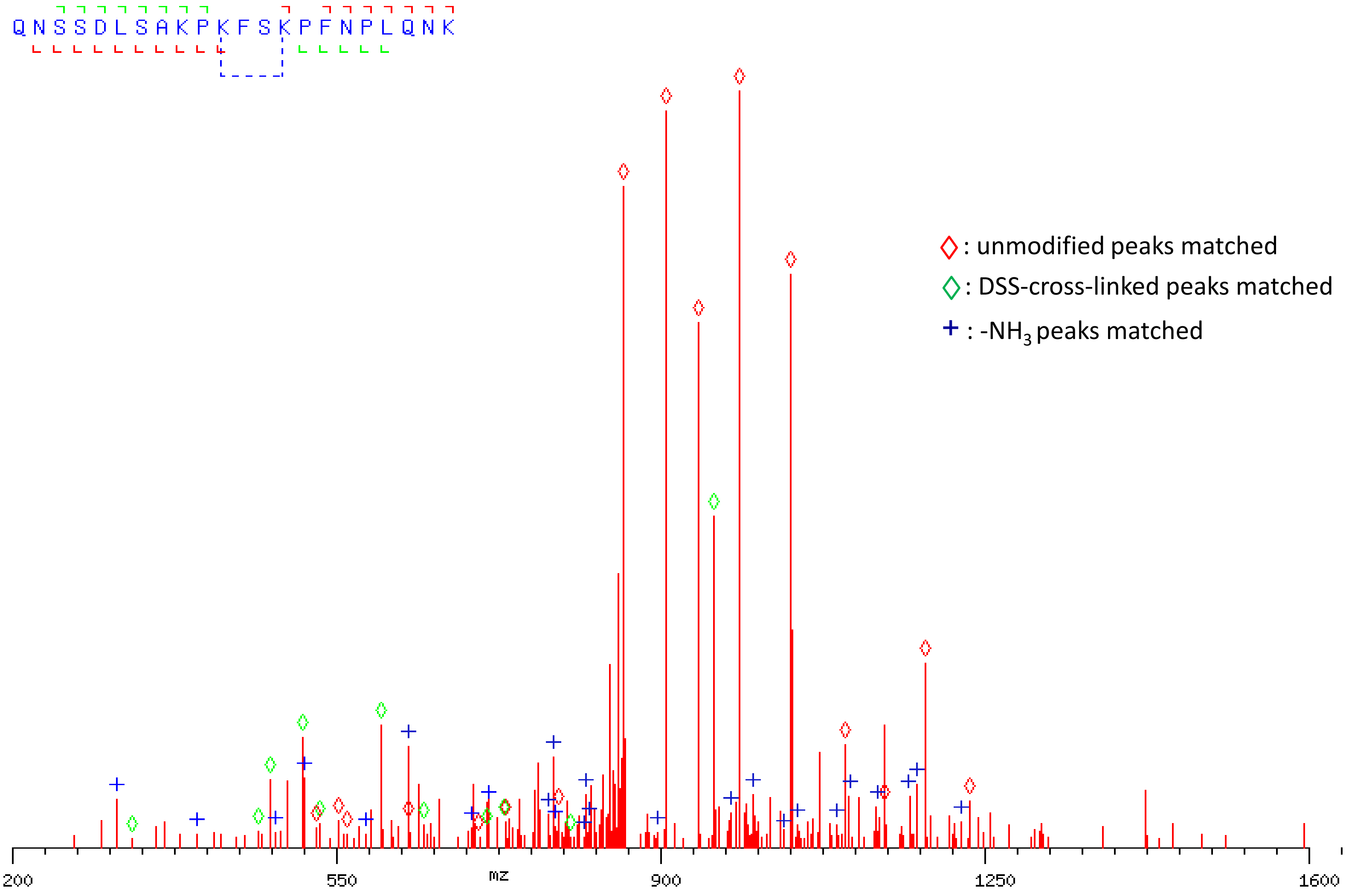
Q N S S D L S A K P K F S K P F N P L Q N K
: unmodified peaks matched
: DSS-cross-linked peaks matched
+ : -NH3 peaks matched
200
550
mz
900
1250
1600

r K151-K254

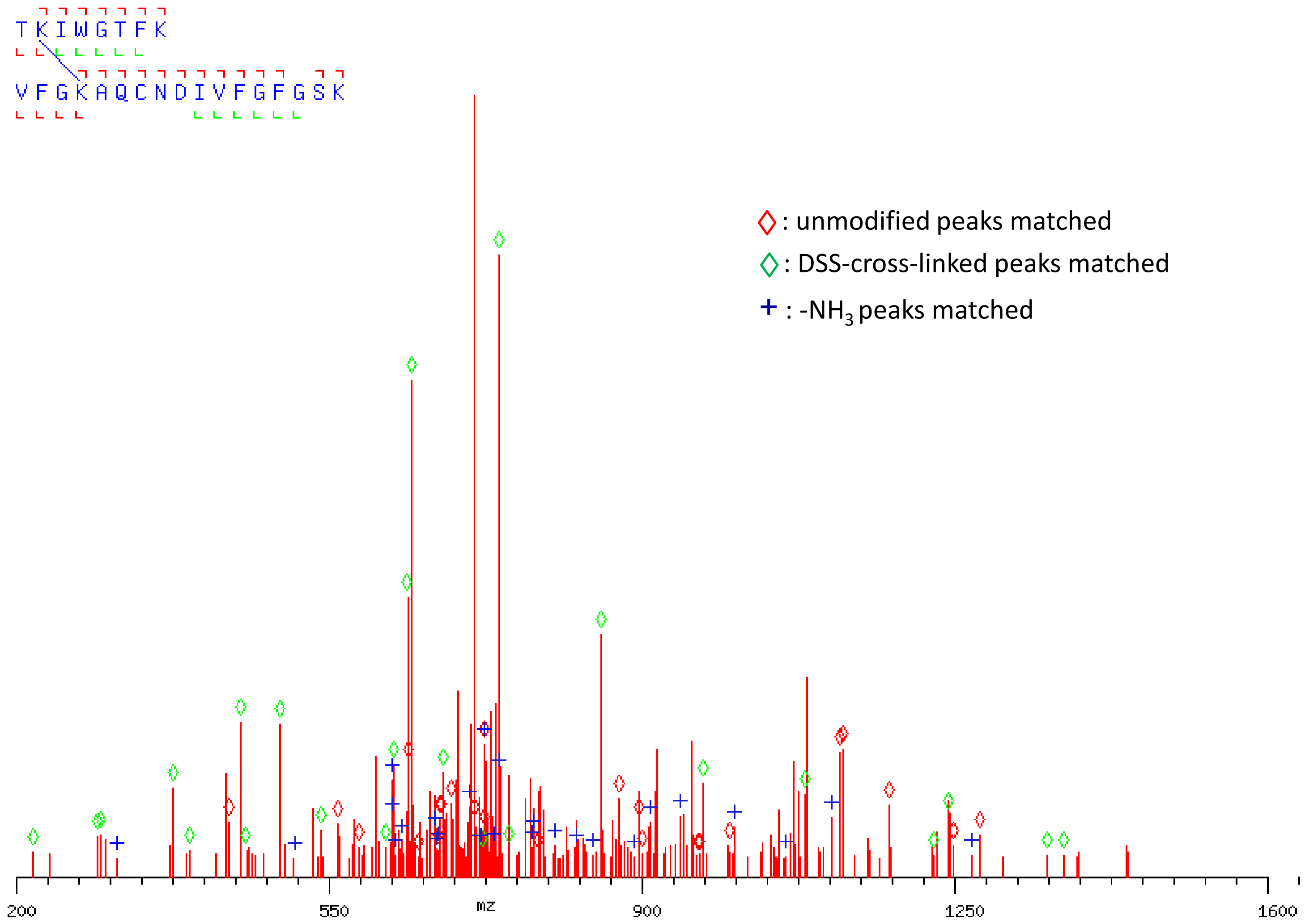

T K I W G T F K
V F G K A Q C N D I V F G F G S K
: unmodified peaks matched
: DSS-cross-linked peaks matched
: -NH$_3$ peaks matched
200
550
mz
900
1250
1600

S

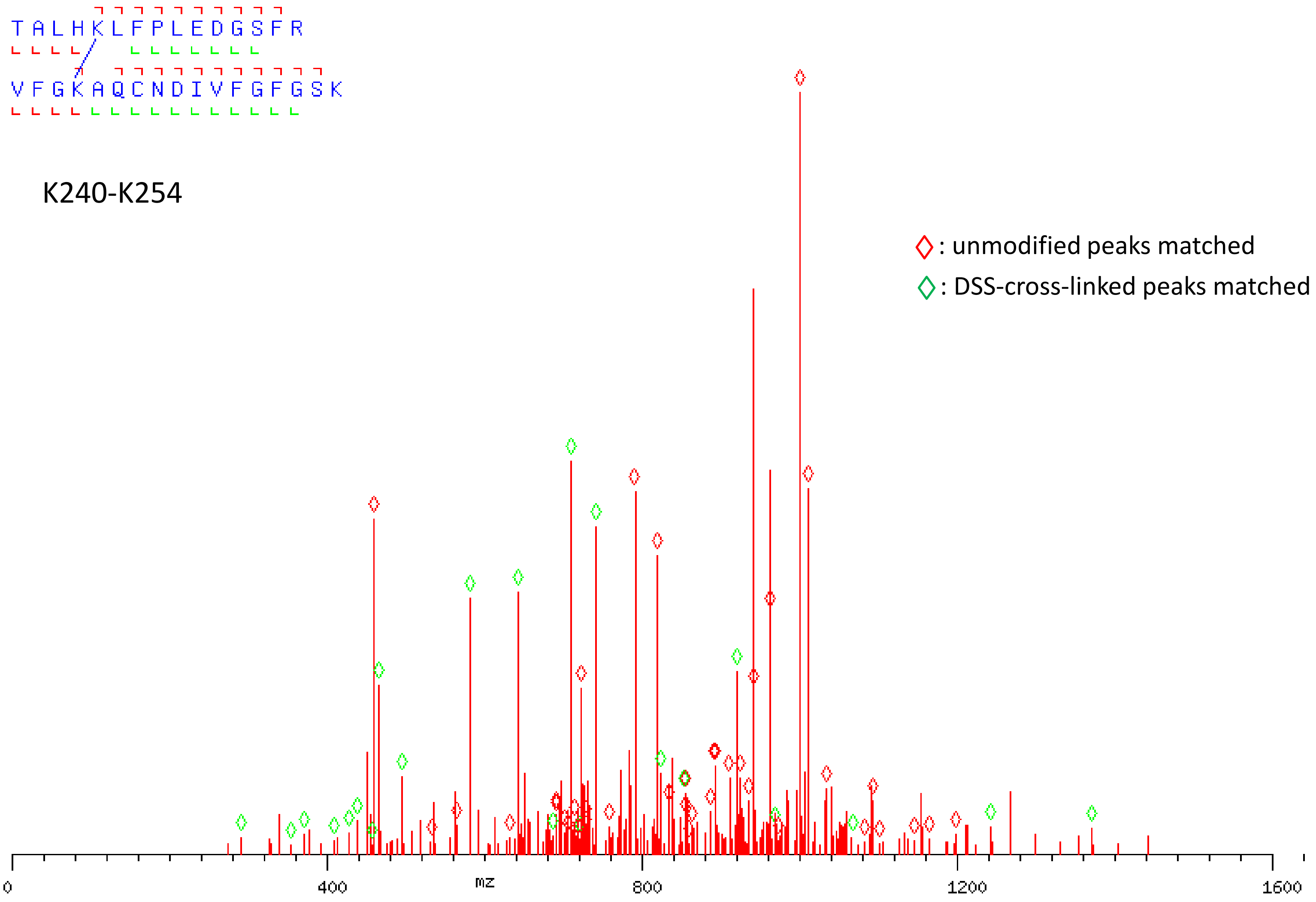

T A L H K L F P L E D G S F R
V F G K A Q C N D I V F G F G S K
K240-K254
: unmodified peaks matched
: DSS-cross-linked peaks matched
0
400
mz
800
1200
1600

t K398-K442

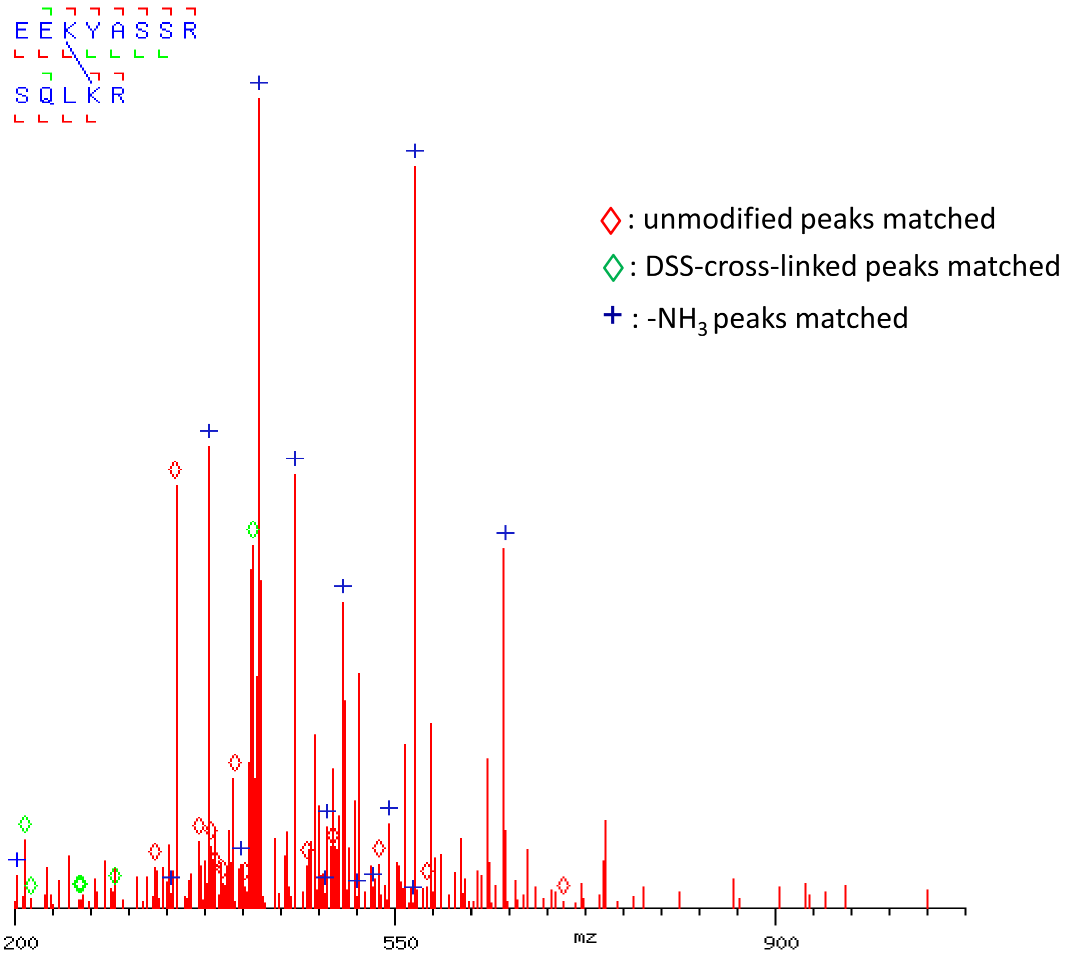

E E K Y A S S R
S Q L K R
◊ : unmodified peaks matched
◊ : DSS-cross-linked peaks matched
+ : -NH3 peaks matched
200
550
mz
900

u K860-K864

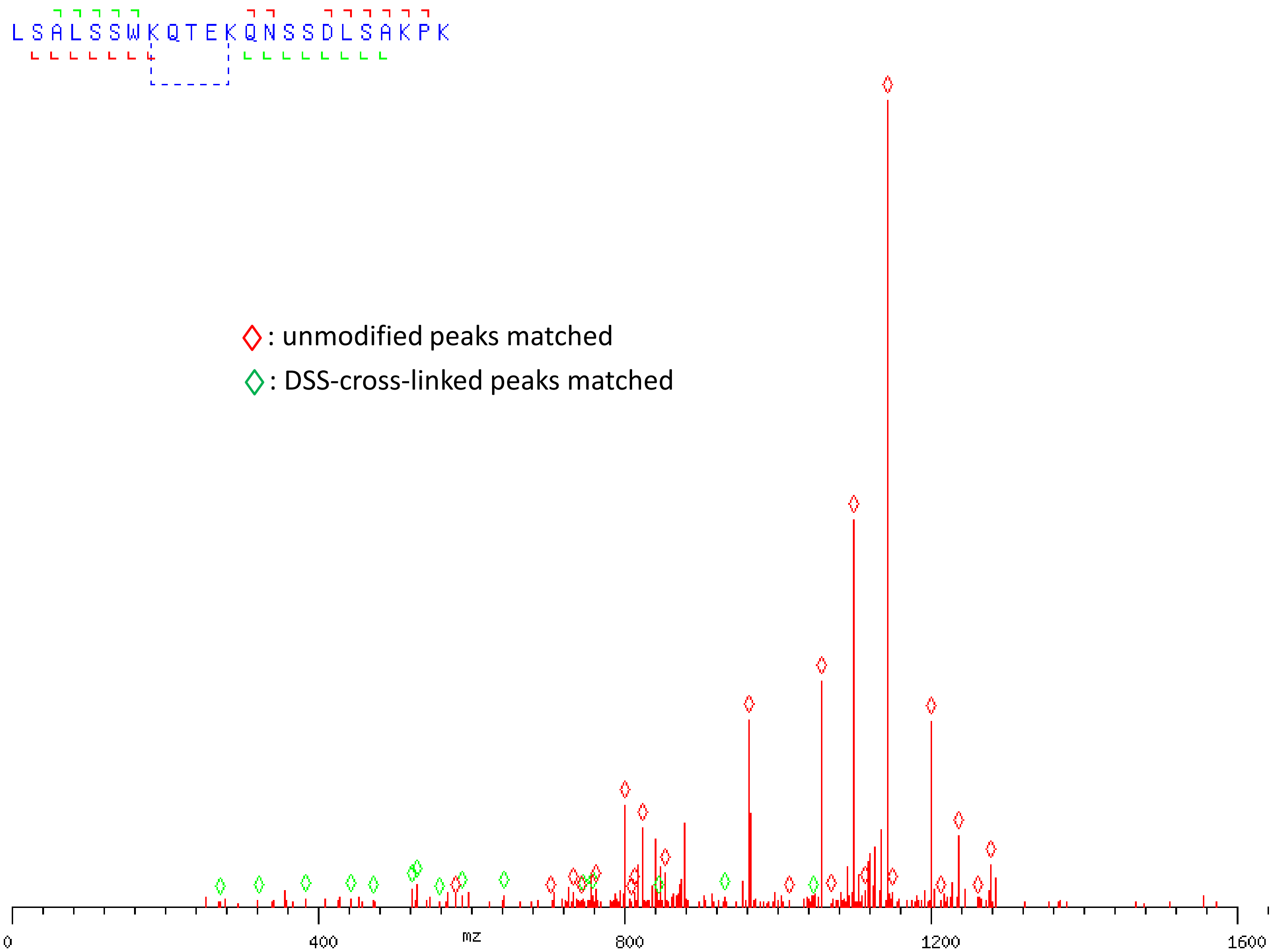

L S A L S S W K Q T E K Q N S S D L S A K P K
: unmodified peaks matched
: DSS-cross-linked peaks matched
0
400
mz
800
1200
1600

V K864-K873

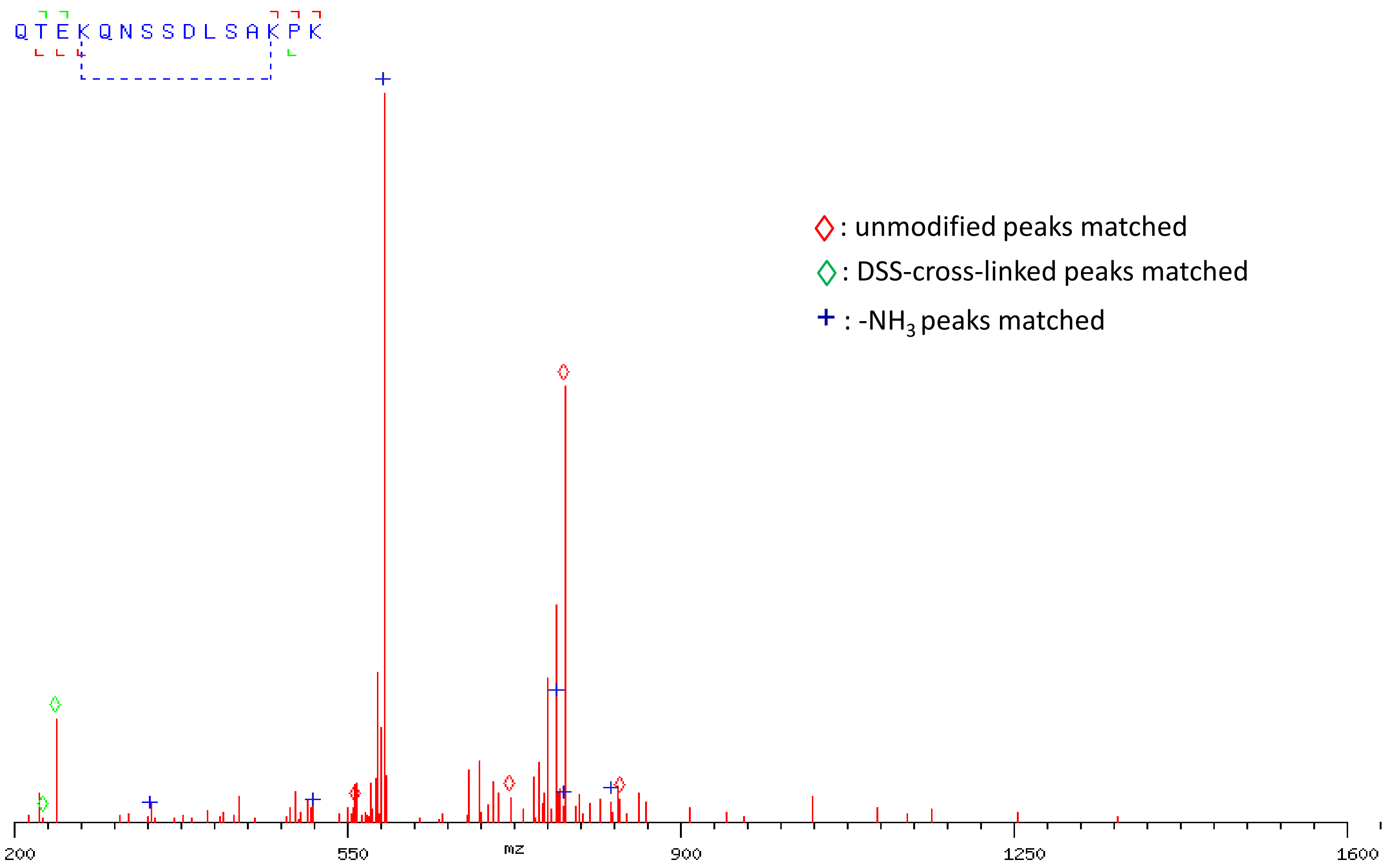

Q T E K Q N S S D L S A K P K
: unmodified peaks matched
: DSS-cross-linked peaks matched
: -NH_3 peaks matched
200
550
mz
900
1250
1600

W

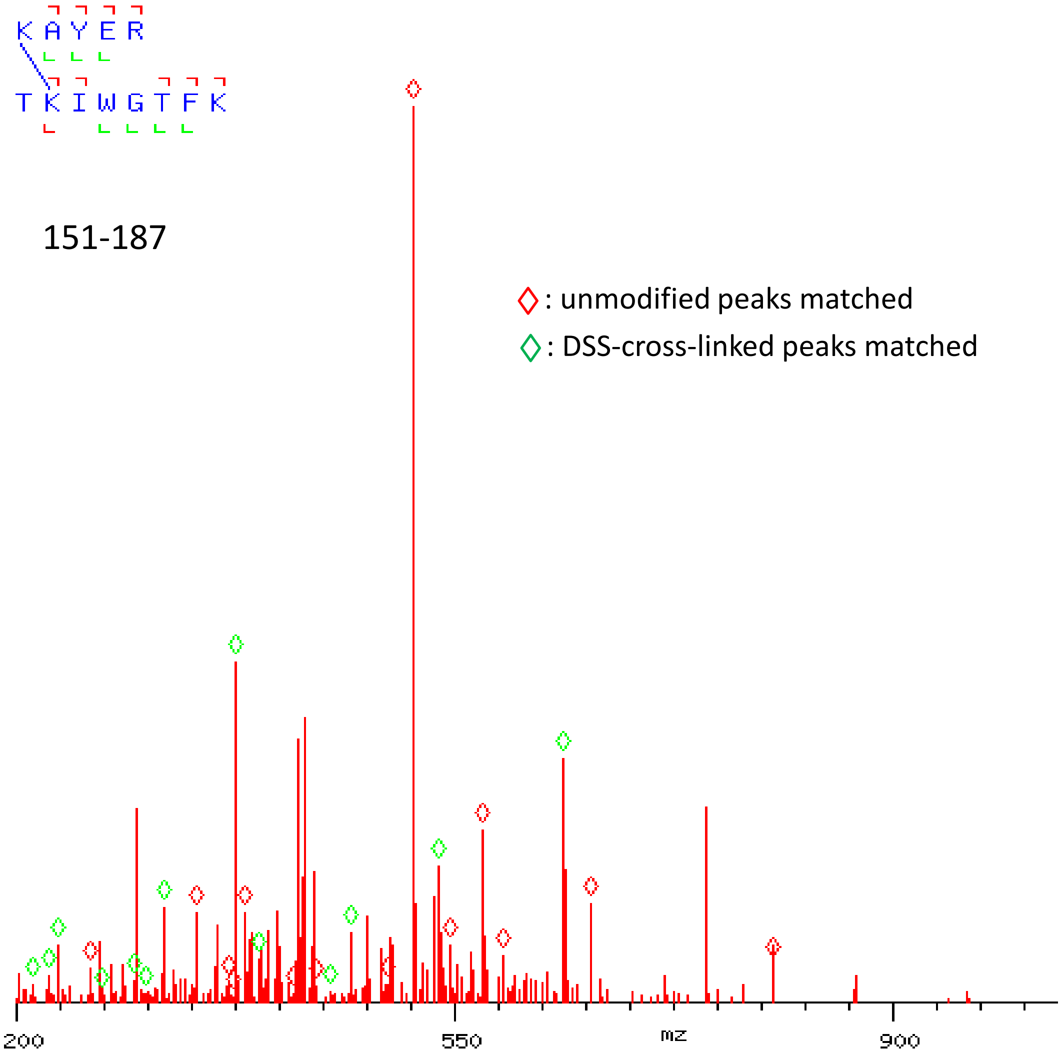
K A Y E R
T K I W G T F K
151-187
: unmodified peaks matched
: DSS-cross-linked peaks matched
200
550
mz
900

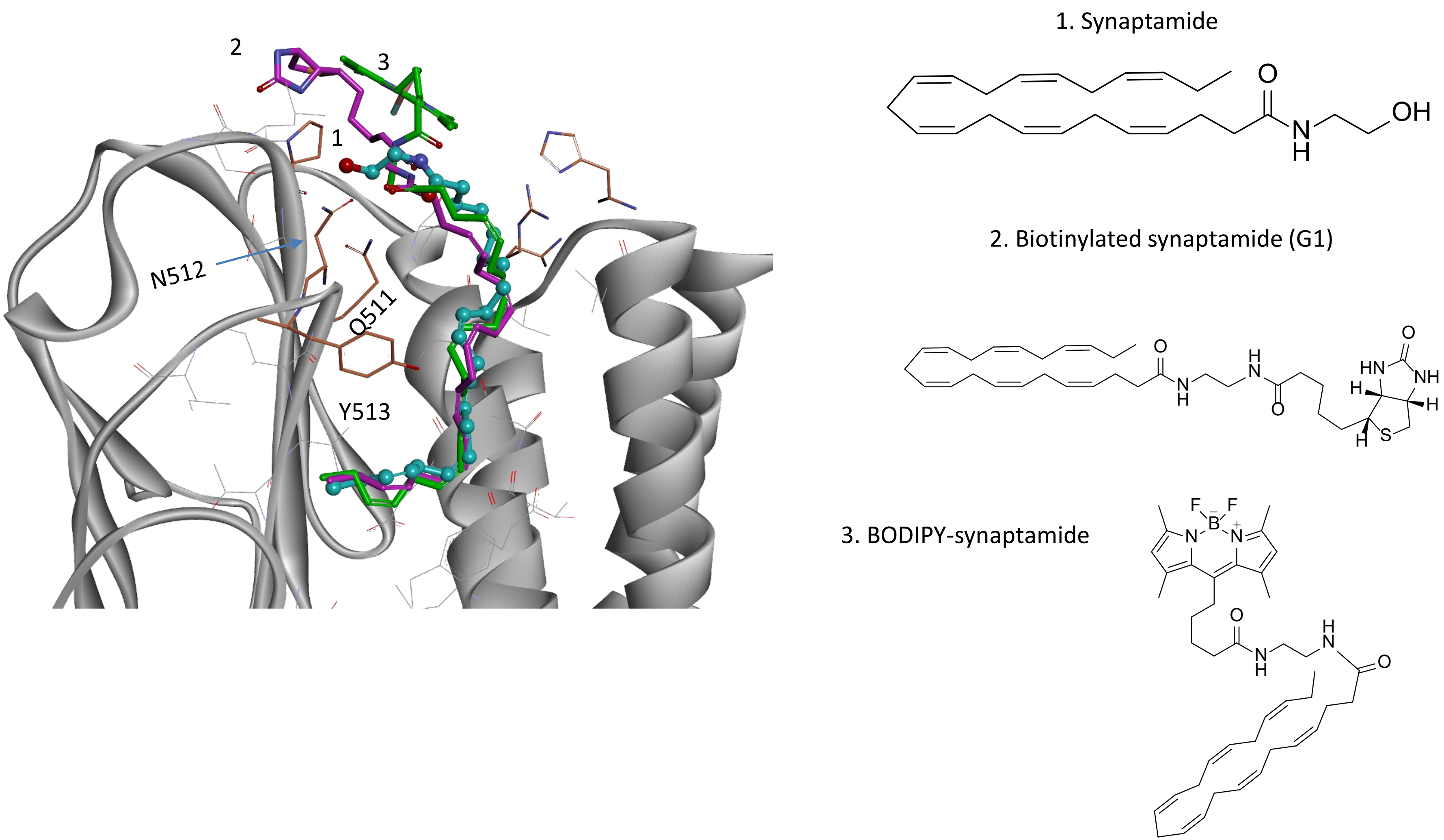

Supplementary Figure 6. Docking of synaptamide and its analogs to the GAIN domain of GPR110. All three analogs that differ in the headgroup fit well into the binding pocket in the GAIN domain. Synaptamide (cyan-colored carbon) is displayed in stick-and-ball, while biotinylated synaptamide (magenta-colored carbon) and bodipy-synaptamide (green-colored carbon) are shown in sticks. Oxygen, nitrogen, sulfur and fluorine atoms are shown in red, blue, deep yellow and light green respectively. Amino acid residues predicted to potentially interact with synaptamide are represented in sticks (organ-colored carbon).

```
GPR110          GNITAKCESS------GWQVIRETCVLSLLEELNKNFSMIVGNATEAAVSSFVQNLSVII
GPR116          GTITYKCVGS------QWEEKRNDCISAPINSLLQMAKALIKSPSQDEMLPTYLKDLSIS
GPR113          GIVRRLCGADG-----VWGPVHSSCTDARLLALFTRTKLLQAGQGSPAEEVPQILAQLPG
ADGRL1          GIASFQCLPALG-LWNPRGPDLSNCTSPWVNQVAQKIKSGENAANIASELARHTRGSIYA
BAI3            GTTSRRCSLSLHGVAFWEQPSFARCISNEYRHLQHSIKEHLAKGQRMLAGDGMSQVTKTL

GPR110          RQNPSTTVGNLASVVSILSNISSLSLASH-------------FRVSNSTMEDVISIADNI
GPR116          IDKAEHEISSSPGSLGAIINILDLLSTVP-------------TQVNSEMMTHVLSTVNVI
GPR113          QAAEASSPSDLLTLLSTMKYVAKVVAEAR-------------IQLDRRALKNLLIATDKV
ADGRL1          GDVSSSVKLMEQLLDILDAQLQALRPIERESAGKNYNKMHKRERTCKDYIKAVVETVDNL
BAI3            LDLTQRKNFYAGDLLMSVEILRNVTDTFKR----------ASYIPASDGVQNFFQIVSNL

GPR110          LNSASVTNWTVLLREEKYASSRLLETLENISTLVPP----TALPLNFSRKFIDWKGIPVN
GPR116          LGKPVLNTWKVLQQQWTNQSSQLLHSVERFSQALQSG---DSPPLSFSQTNVQMSSMVIK
GPR113          LDMDTRSLWTLAQARKPWAGSTLLLAVETLACSLCP----QDHPFAFSLPNVLLQSQLFG
ADGRL1          LRPEALESWKDMNATEQVHTATMLLDVLEEGAFLLADNVREPARFLAAKENVVLEVTVLN
BAI3            LDEENKEKWEDAQQIYPGSIELMQVIEDFIHIVGMGMMDFQNSYLMTGNVVASIQKLPAA

GPR110          KSQLKRGYSYQIKMCPQNTSIPIRGRVLIGSDQFQRSLPETIISMASLTLGNILPVSKNG
GPR116          SSHPETYQQRFVFPYFDLWGNVVIDKSYLENLQSD----SSIVTMAFPTLQAILAQDIQE
GPR113          PTFPADYSISFPTRPPLQAQIPRHSLAPLVRNGTEISITSLVLRKLDHLLPSNYGQGLGD
ADGRL1          TEGQVQELVFPQEEYPRKNSIQLSAKTIKQNSRNGVVKVVFILYNNLGLFLSTENATVKL
BAI3            SVLTDINFPMKGRKGMVDWARNSEDRVVIPKSIFTPVSSKELDESSVFVLGAVLYKNLDL

GPR110          N------------AQVNGPVISTVIQNYSIN--EVFLFFSKIESNLSQ----PHCVFWDF
GPR116          N------------NFAESLVMTTTVSHNTTMPFRISMTFKNNSPSGGE----TKCVFWNF
GPR113          S------------LYATPGLVLVISIMAGDRAFSQGEVIMDFGNTDGS----PHCVFWDH
ADGRL1          AGEAGPGGPGGASLVVNSQVIAASINKESSRVFLMDPVIFTVAHLEDKNHFNANCSFWNY
BAI3            ILPTLRN-----YTVINSKIIVVTIRPEPKTTDSFLEIELAHLANGTLN---PYCVLWDD

GPR110          S-HLQ----WNDAGCHLVNETQD-IVTCQCTHLTSFSILMSPFVP---STIFPVVKWITY
GPR116          R-LANNTGGWDSSGCYVEEGDGD-NVTCICDHLTSFSILMSPDSPDPSSLLGILLDIISY
GPR113          S-LFQGRGGWSKEGCQAQVASASPTAQCLCQHLTAFSVLMSPHTVP----EEPALALLTQ
ADGRL1          S-ERSMLGYWSTQGCRLVESNKT-HTTCACSHLTNFAVLMAHREIYQGRINELLLSVITW
BAI3            SKTNESLGTWSTQGCKTVLTDAS-HTKCLCDRLSTFAILAQ-QPREIIMESSGTPSVTLI
```

Supplementary Figure 7. Sequence alignment of GPR110(Q5T601-1), GPR116(Q8IZF2-1), GPR113(Q8IZF5-1), ADGRL1(O94910-1) and BAI3 (O60242-1). UniProt protein IDs are indicated in parentheses. Q511, N512 and Y513 of GPR110 (green-highlighted) are not conserved in the GAIN domains (~320 residues underlined). GPR113, GPR116 and GPR110 are members of group VI of aGPCR, while ADGRL1 (Latrophilin) and BAI3 belongs to groups I and VII respectively. Yellow-highlighted leucine residues (L566, L990, L752, L838, L856 in GPR110, GPR116, GPR113, ADGRL1 and BAI3, respectively) are the GPCR proteolysis site. Multiple Sequence Alignment is performed using CLUSTALW (https://www.genome.jp/tools-bin/clustalw).

```
GPR115          FRAPET-------------------------------------------------IESVA
GPR111          S-------------------------------------------------------ETIT
GPR110          LFPLEDGSFRVFGKAQCNDIVFGFGSKDDEYTLPCSSGYRGNITAKCESSGWQVIRETCV

GPR115          QGIRKNCPFDYACITD-----------------MVKSSETTSGNIAFIVELLKNIS---T
GPR111          DMLLQKCPTDLSCVIR-----------------NIQQSPWIPGNIAVIVQLLHNIS---T
GPR110          LSLLEELNKNFSMIVGNATEAAVSSFVQNLSVIIRQNPSTTVGNLASVVSILSNISSLSL

GPR115          DLSDNVTREKMKSYSEVANHILDTAAISNWAFIP--NKNASSDLLQSVNLFARQLHIHNN
GPR111          AIWTGVDEAKMQSYSTIANHILNSKSISNWTFIP--DRNSSYILLHSVNSFARRLFIDKH
GPR110          ASHFRVSNSTMEDVISIADNILNSASVTNWTVLLREEKYASSRLLETLENISTLVPPTAL


GPR115          SENIVNELFIQTKGFHINHNTSEKSLNFSMSMN--NTTEDILGMVQIPRQELRKLWPNAS
GPR111          PVDIS-DVFIHTMGTTISGDNIGKNFTFSMRIN--DTSNEVTGRVLISRDELRKVPS-PS
GPR110          PLNFS-RKFIDWKGIPVNKSQLKRGYSYQIKMCPQNTSIPIRGRVLIGSDQFQRSLP--E

GPR115          QAISIAFPTLGAILREAHLQNVSLPRQVNGLVLSVVLPERLQEIILTFEKINKTRNARAQ
GPR111          QVISIAFPTIGAILEASLLENVTVN----GLVLSAILPKELKRISLIFEKISKSEERRTQ
GPR110          TIISMASLTLGNILPVSKNGNAQVN----GPVISTVIQNYSINEVFLFFSKIESNLSQPH

GPR115          CVGWHSKKRRWDEKACQMMLDIRNEVKCRCNYTSVVMSFSILMSSKS--MTDKVLDYITC
GPR111          CVGWHSVENRWDQQACKMIQENSQQAVCKCRPSKLFTSFSILMSPHI--LESLILTYITY
GPR110          CVFWDFSHLQWNDAGCHLVNETQDIVTCQCT---HLTSFSILMSPFVPSTIFPVVKWITY
```

Supplementary Figure 8. Sequence alignment of GPR110(Q5T601-1), GPR115(Q8IZF3-1) and GPR111(Q8IZF7-1). UniProt protein IDs are indicated in parentheses. Q511, N512 and Y513 of GPR110 (green-heighted) are not conserved in the GAIN domains (~320 residues underlined). Yellow-highlighted L566 is the GPCR proteolysis site of GPR110. Multiple Sequence Alignment is performed using CLUSTALW (https://www.genome.jp/tools-bin/clustalw).

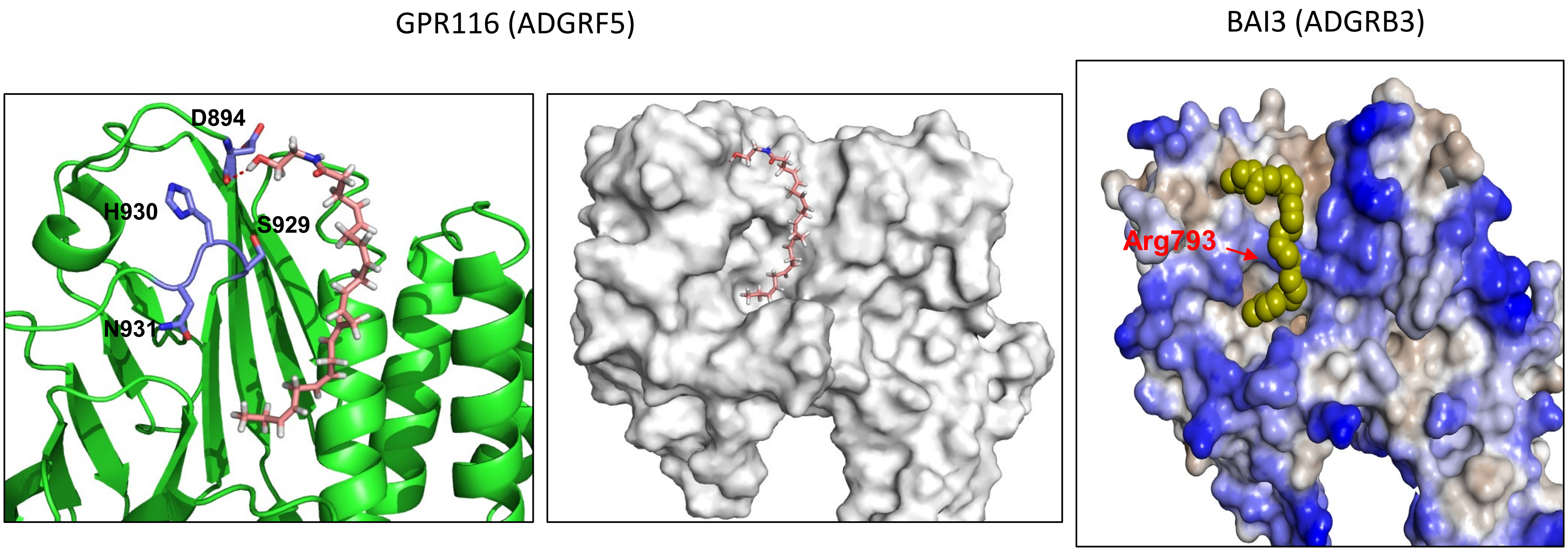

Supplementary Figure 9. Docking of synaptamide to the GAIN domain of GPR116 (ADGRF5) and BAI3 (ADGRB3). Residues Q511, N512, and Y513 are not conserved thus their interaction with synaptamide in GPR110 molecule do not exist in GPR116 and BAI3 (pdb 4DLO). Only one H-bond (D894 to the headgroup of synaptamide) is formed in the model of GPR116. The predicted binding affinity of GPR116 to synaptamide is significantly weaker compared to GPR110 (-5.16 kcal/mol vs -6.71 kcal/mol). S929, H930, and N931 of GPR116 are the corresponding residues to Q511, N512, and Y513 of GPR110. Synaptamide does not fit into the hydrophobic groove in the GAIN of BAI3 where residue Arg793 blocks the binding. Synaptamide is displayed in sticks or space-filling. Protein surface of BAI3 is colored with hydrophobicity (blue, polar; yellow-gray, hydrophobic).

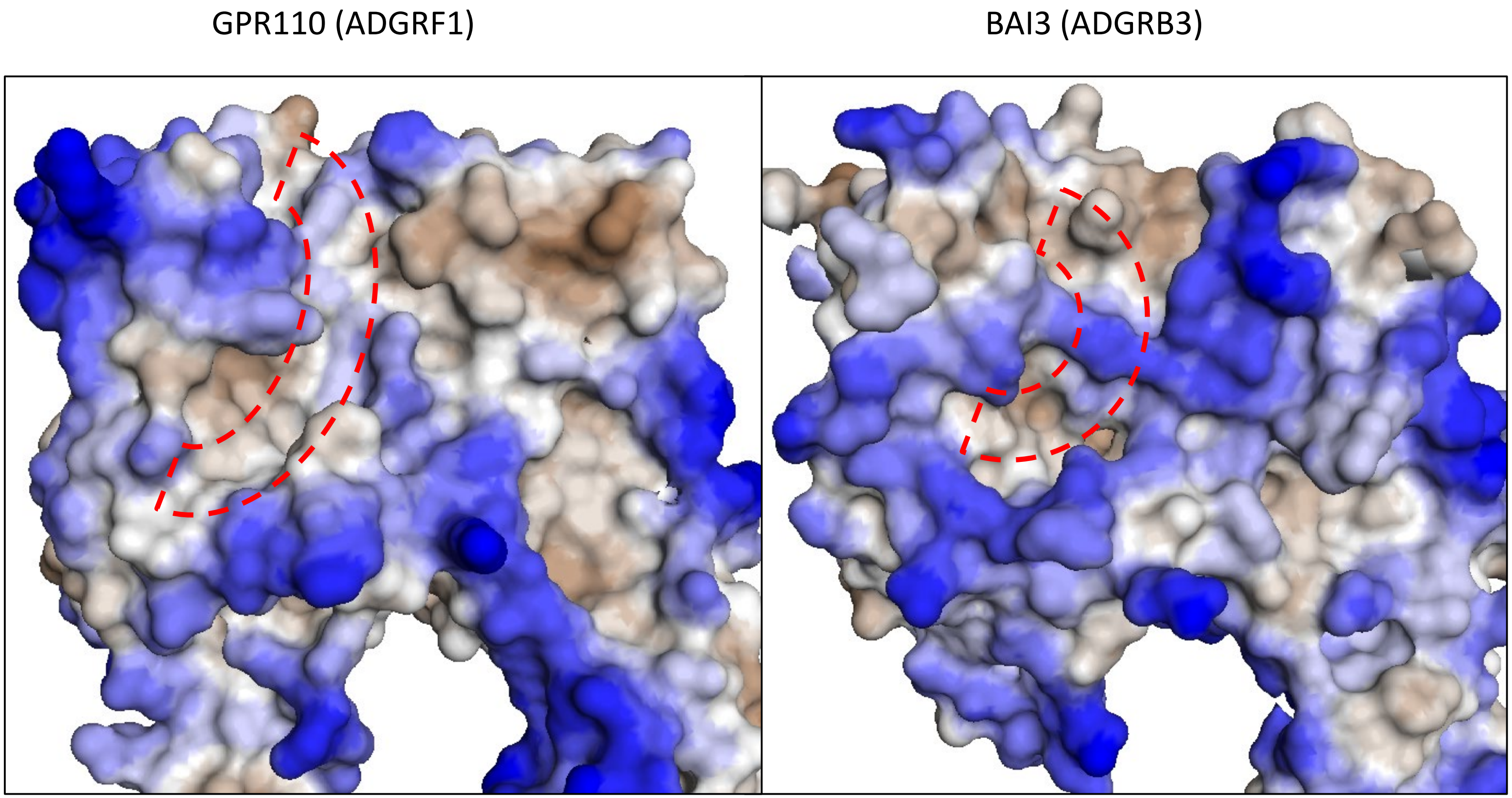

Supplementary Figure 10. Comparison of the hydrophobic groove between GPR110 and BAI3 (ADGRB3). Protein surface is colored with hydrophobicity (blue, polar; yellow-gray, hydrophobic). The hydrophobic grooves are illustrated by red dashed lines.

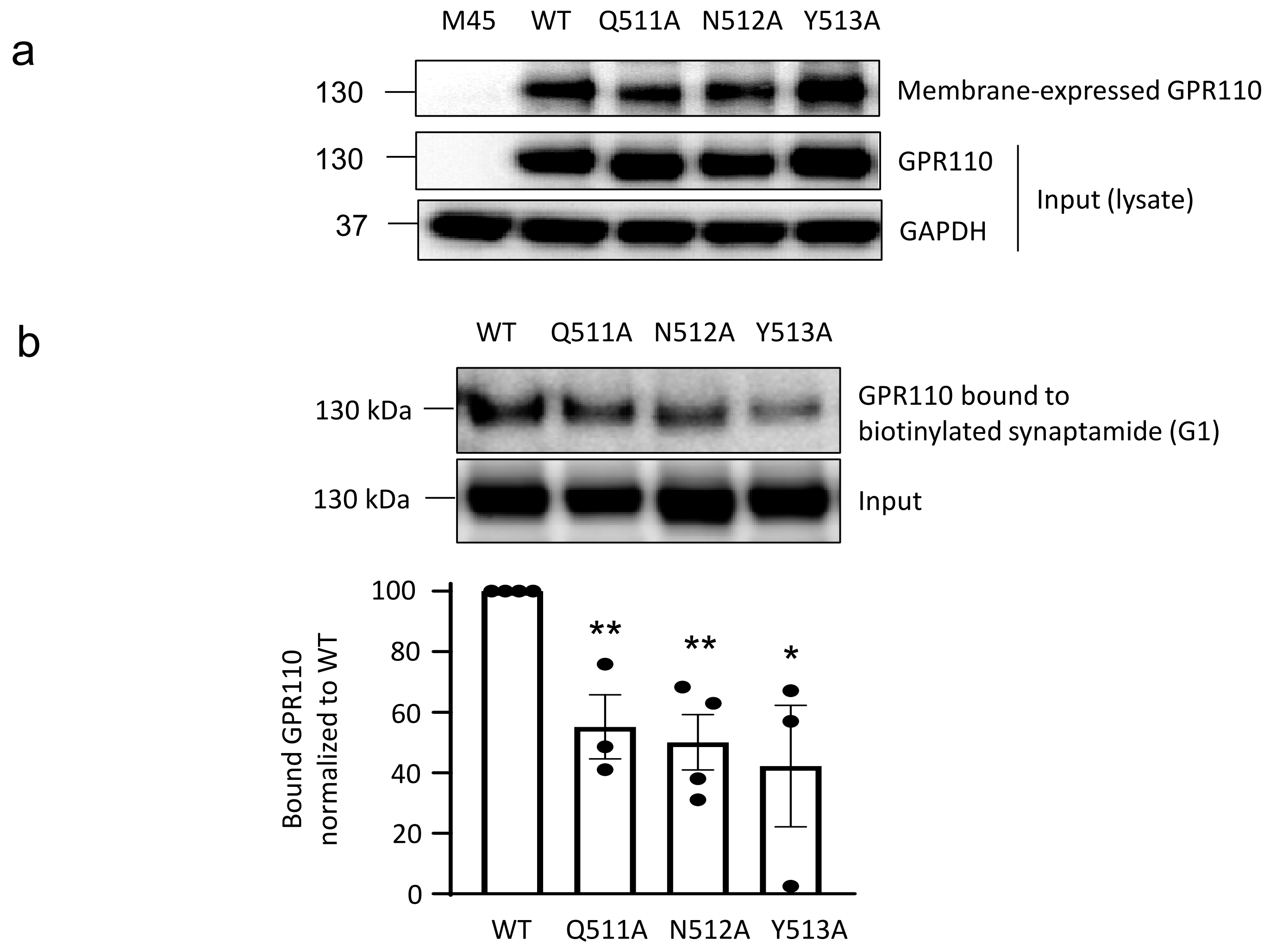

Supplementary Figure 11. Effect of mutations on the ligand binding to GPR110. a, Mutations did not affect cell surface expression of GPR110. Transfected HEK cells were labeled with a membrane-impermeable biotinylated crosslinker. The biotinylated cell surface proteins were enriched with streptavidin beads. b, Mutations impaired the ligand binding to GPR110. Transfected HEK cells were lysed in PBS containing 0.5% Triton X-100 and protease inhibitors. The lysate was treated with biotinylated synaptamide (G1) followed by incubation with streptavidin beads. Membrane-expressed GPR110 (a) or G1-bound GPR110 (b) was detected by Western blotting using anti HA antibody. The vertical axis in B represents the band intensity ratio of G1-bound GPR110 over total expression level (input). Statistical analysis was performed using unpaired Student's t-test (n=3 or 4). *$p < 0.05$, **$p < 0.01$.

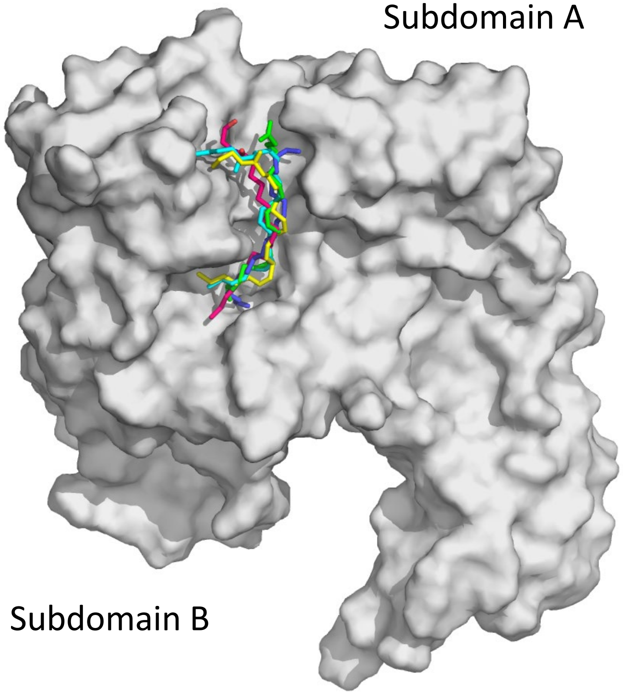

Fatty acids products	Predicted binding affinity (Kcal/mol)
Synaptamide (cyan)	-6.71
DHA (Docosahexaenoic acid)(green)	-5.79
OEA (oleoylethanolamine) (red)	-6.23
AEA (arachidonoylethanolamine, or anandamide) (yellow)	-5.76
PEA (Palmitoylethanolamide) (blue)	-6.08

Supplementary Figure 12. Docking of synaptamide and other structural analogs to the GAIN domain of GPR110. Synaptamide fits well into a pocket at the interface of subdomains A and B. Synaptamide shows higher binding affinity than any other analogs. The model of GAIN (~256-580 aa) was constructed based on the crystal structure of BAI3 (ADGRB3, pdb 4DLO). The binding models were predicted by the MOE Dock and the binding affinity was evaluated using the GBVI/WSA score. GPR110 GAIN domain is depicted in surface representations. The analogs are shown in sticks (synaptamide in cyan, DHA in green, OEA in red, AEA in yellow, PEA in blue).

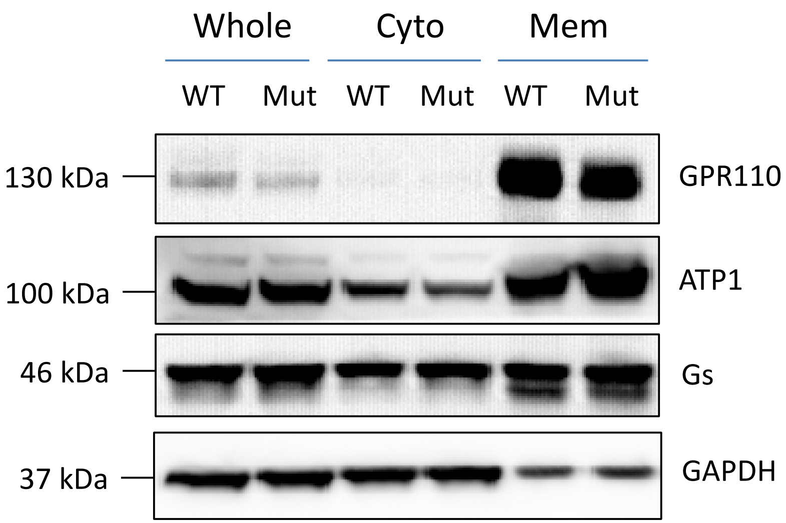

Supplementary Figure 13. Membrane separated by cellular fractionation. HEK cells overexpressing GPR110 WT (WT) or GPR110 mutants along with Gs protein were subjected to fractionation by differential centrifugation. The representative western blotting shows that membrane proteins (.i.e., Na^+/K^+ ATPase or ATP1, GPR110 and Gs) were isolated and enriched in the membrane fraction suitable for [^{35}S]GTPγS-binding assay. Whole, whole cell; Cyto, cytoplasm; Mem, membrane. GAPDH, Glyceraldehyde 3-phosphate dehydrogenase. Mut, N512A/Y513A.

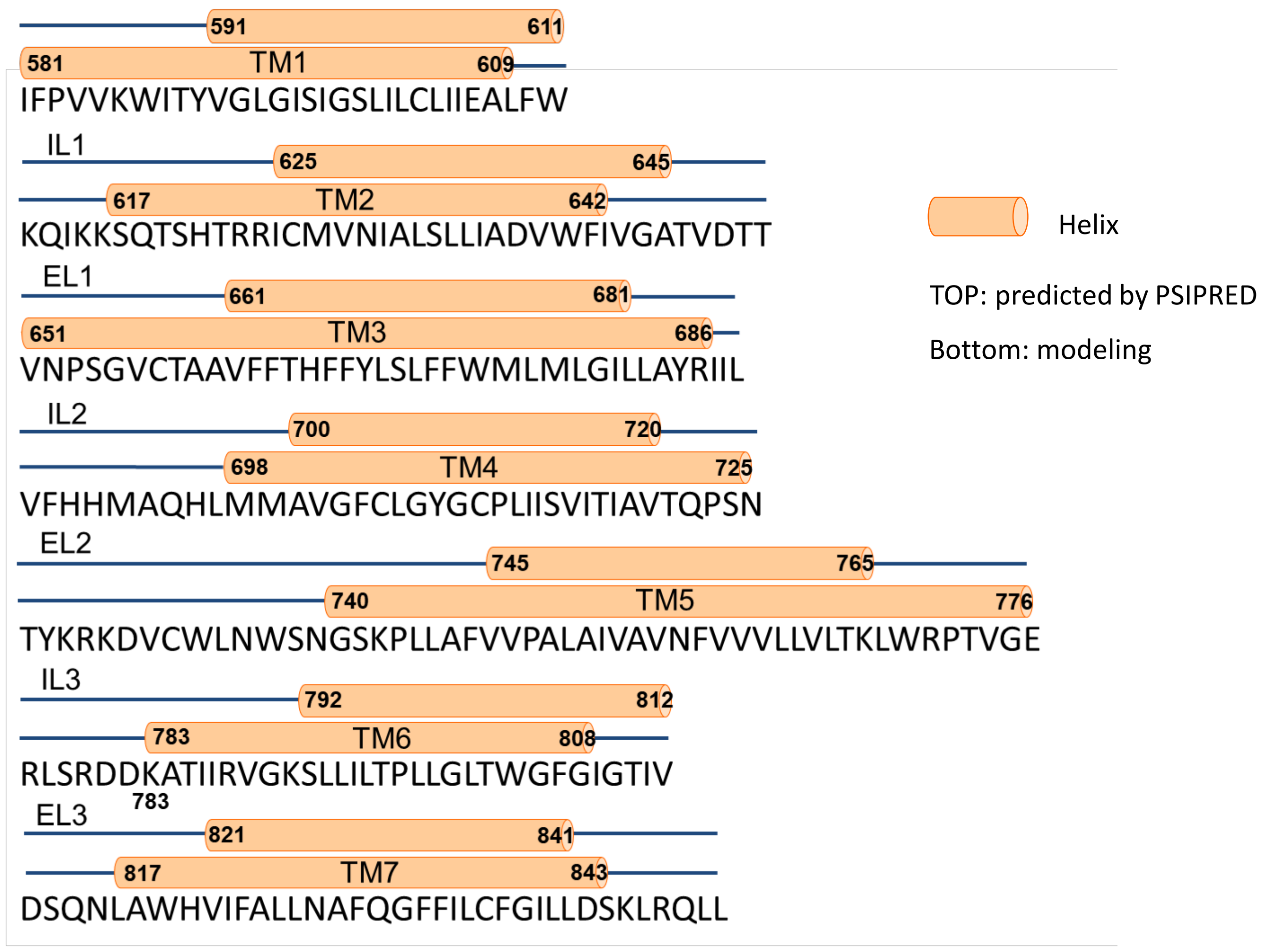

Supplementary Figure 14. TM and intracellular domains of GPR110 predicted by PSIPRED or modeling. The model was constructed using the structures of corticotropin-releasing factor receptor 1 (pdb 4K5Y) and Glucagon receptor (pdb 4L6R) as templates.

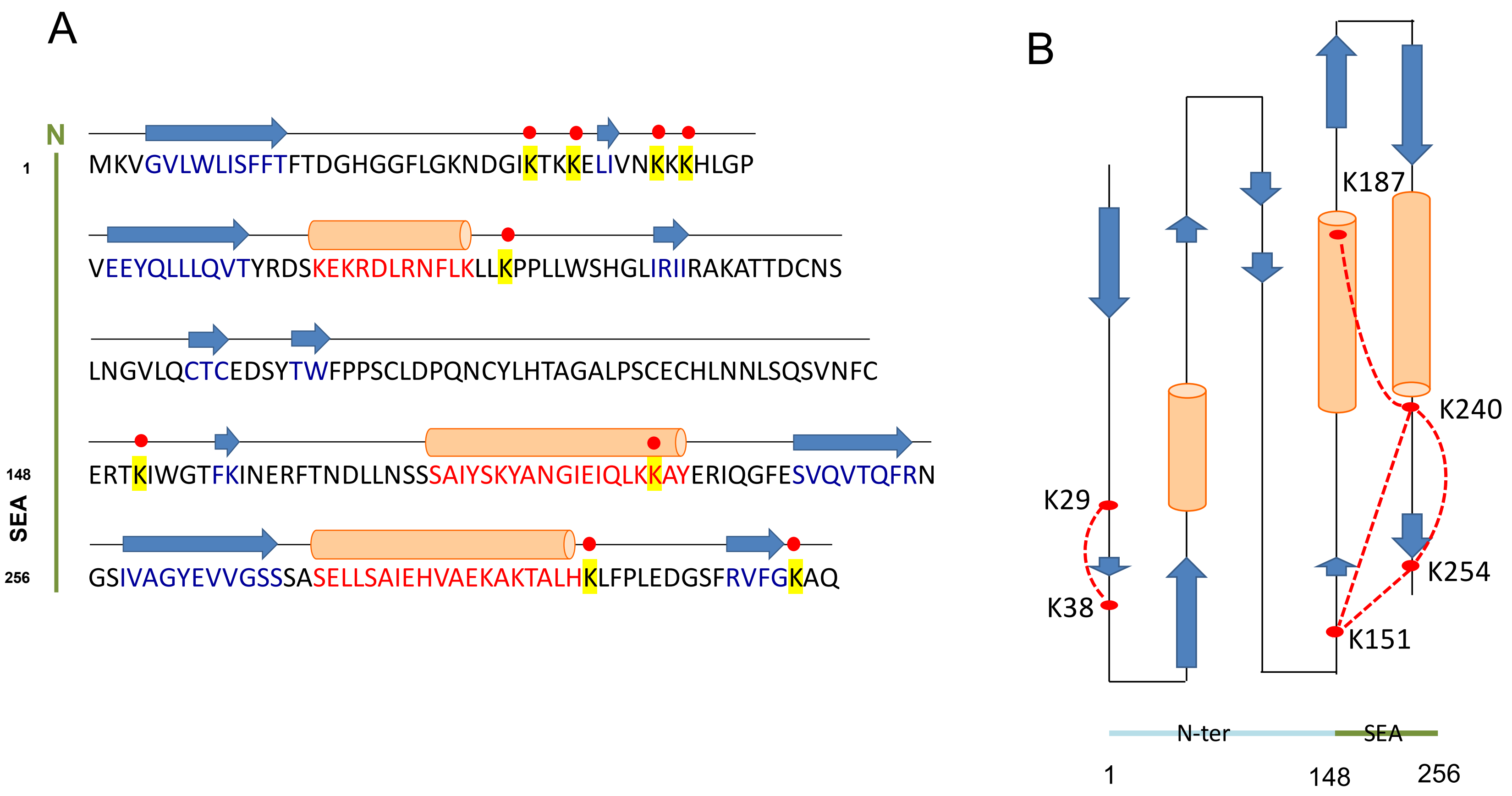

Supplementary Figure 15. Secondary structure and the cross-linking profile of the *N*-terminal and SEA domains of GPR110. A, The secondary structure predicted by PSIPRED. B, Through-space cross-linking profile. Cross-linked lysine residues are red-marked in the secondary structure or yellow-highlighted in the primary sequence.

Supplementary Table 1. Cross-linked lysine pairs identified in GPR110

Crosslinked residues	Crosslinked peptides	Mass accuracy (ppm)	MS/MS Validated	Cross-linking type	Domain located
K29-K38	N[25-31]K-E[33-39]K	0.2	√	Through-space	Nter
K31-K32	T[30-38]K	0.8	√	Loop	N-ter
K38-K39	E[33-40]K	0.5	√	Loop	N-ter
K39-K40	K[39-57]R	0.6	√	Loop	N-ter
K151-K157	T[150-161]R	0	√	Loop	SEA
K151-K187	T[150-157]K-K[187-191]R	1.0	√	Through-space	SEA
K151-K254	T[150-157]K-V[251-267]K	0.4	√	Through-space	SEA
K151-K442	T[150-157]K-S[439-443]R	0.3	√	Through-space	SEA-GAIN
K186-K187	Y[177-191]R	0.5	√	Loop	SEA
K187-K240	K[187-191]R-T[236-250]R	0.2	√	Through-space	SEA
K235-K240	A[234-250]R	0.4	√	Loop	SEA
K240-K254	T[236-250]R-V[251-267]K	0.4	√	Through-space	SEA-GAIN
K398-K427	E[396-403]R-K[427-432]K	0.8	√	Through-space	GAIN
K398-K438	E[396-403]R- G[433-442]R	0.7	√	Through-space	GAIN
K398-K442	E[396-403]R-S[439-443]R	0.1	√	Through-space	GAIN
K427-K432	K[427-438]K	0.8	√	Loop	GAIN
K427-K438	K[427-432]K-E[396-403]R	0.5	√	Through-space	GAIN
K427-K442	K[427-432]K-S[439-443]R	0.6	√	Through-space	GAIN
K432-K438	F[428-442]K	0.5	√	Loop	GAIN
K438-K442	G[433-443]R	0.9	√	Loop	GAIN
K783-K852	D[781-788]R-Q[847-860]K	0.5	√	Through-space	TM6-Cter
K852-K860	Q[847-864]K	0.4	√	Long loop*	Cter
K860-K864	L[853-875]K	0.6	√	Loop	Cter
K864-K873	Q[861-875]K	1.4	√	Long loop	Cter
K875-K878	Q[865-886]K	0.7	√	Loop	C-ter

Supplementary Table 2. Label-free quantitation of cross-linked peptides*

Domains	Crosslinked lysine pairs	Intensity ratio OEA/DMSO	P (Student's T test, n=3)
	K29-K38	0.89	0.23
	K31-K32	0.87	0.17
	K38-K39	0.85	0.12
N-terminus	K39-K40	0.74	0.09
	K151-K157	0.73	0.06
	K151-K187	0.90	0.31
	K151-K254	0.72	0.12
	K186-K187	0.82	0.21
	K187-K240	0.82	0.01
	K235-K240	1.00	0.46
SEA	K240-K254	0.69	0.18
SEA-GAIN	K151-K442	0.61	0.11
	K398-K427	0.89	0.08
	K398-K438	0.83	0.02
	K398-K442	1.04	0.30
	K427-K432	0.91	0.25
	K427-K438	0.97	0.33
	K427-K442	0.77	0.10
	K432-K438	0.93	0.40
GAIN	K438-K442	1.24	0.24
TM6-C-terminus	K783-K852	1.04	0.46
	K852-K860	0.72	0.04
	K860-K864	0.77	0.09
	K864-K873	0.88	0.23
C-terminus	K875-K878	1.12	0.30

* No significant changes (>1.5 fold-change and p <0.05) were observed for cross-linked peptides regardless of treatment with oleoylethanolamine (OEA), or vehicle DMSO.

Supplementary Table 3. Label-free quantitation of mono-linked peptides*

			Normalized Intensity			Intensity ratio			P (Student's T test, n=3)		
Domain located	Monolink detected	m/z Observed	DMSO	OEA	Syn	Syn/OEA	Syn/DMSO	OEA/DMSO	Syn vs OEA	Syn vs DMSO	OEA vs DMSO
N-ter	K32	500.3077	6960.609	6521.118	5857.408	0.898221	0.841508	0.93686	0.152627	0.031536	0.267429
N-ter	K40	781.4289	1757.977	1599.069	1825.12	1.141364	1.038193	0.909608	0.291014	0.8029	0.450679
N-ter	K73	600.3693	9174.365	7863.353	6991.973	0.889185	0.762121	0.8571	0.422078	0.019971	0.184477
SEA	K151	568.8207	3095.594	3551.221	3363.728	0.947203	1.086618	1.147186	0.714652	0.645141	0.267908
SEA	K157	710.3848	4591.791	0.991214	0.583413	0.588584	0.000127	0.000216	0.316645	0.00021	0.00021
SEA	K240	629.6724	9938.931	10148.32	9694.262	0.955258	0.975383	1.021067	0.75004	0.824088	0.884885
SEA	K254	1015.508	5150.789	7260.336	6335.822	0.872662	1.230068	1.409558	0.459409	0.077504	0.161254
GAIN	K398	563.2746	788.776	745.1134	648.9671	0.870964	0.822752	0.944645	0.712889	0.693536	0.869902
GAIN	K432	736.9113	863.4107	812.9239	722.2311	0.888436	0.836486	0.941526	0.190866	0.102379	0.246563
GAIN	K438	620.3689	5916.136	5727.947	5645.894	0.985675	0.954321	0.968191	0.667638	0.350069	0.474966
TM-6	K783	544.3033	4890.754	4331.06	4553.559	1.051373	0.931055	0.885561	0.281265	0.152847	0.04055
C-ter	K864	908.9685	96.12957	84.62568	117.2051	1.384982	1.21924	0.880329	0.178828	0.500867	0.64346
C-ter	K873	738.3994	21094.95	22716.89	21447.85	0.944137	1.016729	1.076888	0.641519	0.907213	0.631075

* No significant changes (>1.5 fold-change and p <0.05) were observed for mono-linked peptides regardless of treatment with synaptamide (Syn), or oleoylethanolamine (OEA), or vehicle DMSO.

Supplementary Table 4. The C_α-C_α distance of the cross-linked lysine residues predicted in the GPR110 GAIN model.

**Cross-linked lysine pairs**	**C_α-C_α (Å)**
K398-K427	28.2
K398-K438	32.1
K398-K442	40.4
K427-K432	6.9
K427-438	24.2
K427-K442	24.6
K432-K438	18.0
K438-K442	10.1
