# Supplementary material for: Synaptamide activates the adhesion GPCR GPR110 (ADGRF1) through GAIN domain binding
Source: Commun Biol. 2020 Mar 6;3:109. doi: 10.1038/s42003-020-0831-6 (PMC7060178; doi:10.1038/s42003-020-0831-6)
Supplement: Supplementary file 2 — Description of Additional Supplementary Files [file 42003_2020_831_MOESM2_ESM.pdf]

Description of additional supplementary items

1. Supplementary Data 1
   This file contains all source data underlying the graphs presented in the main figures and the exact p values in Excel format.

2. Supplementary Data 2
   This file contains the model of the GPR110 GAIN domain in pdb format.

3. Supplementary Data 3.
   This file contains the model of the 7TM and C-terminal regions in pdb format.
